# Novel insights into Notum and glypicans regulation in colorectal cancer

**DOI:** 10.18632/oncotarget.5652

**Published:** 2015-10-20

**Authors:** Mariangela De Robertis, Maddalena Arigoni, Luisa Loiacono, Federica Riccardo, Raffaele Adolfo Calogero, Yana Feodorova, Dessislava Tashkova, Vesselin Belovejdov, Victoria Sarafian, Federica Cavallo, Emanuela Signori

**Affiliations:** ^1^ Laboratory of Molecular Medicine and Biotechnology, Center of Integrated Research, Campus Bio-Medico University of Rome, 00128 Rome, Italy; ^2^ Laboratory of Molecular Pathology and Experimental Oncology, Institute of Translational Pharmacology, National Research Council (CNR), 00133 Rome, Italy; ^3^ Department of Molecular Biotechnology and Health Sciences, Molecular Biotechnology Center, University of Torino, 10126 Torino, Italy; ^4^ Laboratory of Oncology, IRCCS Casa Sollievo della Sofferenza, 71013-San Giovanni Rotondo (FG), Italy; ^5^ Department of Medical and Surgical Sciences, University of Foggia, 71100 Foggia, Italy; ^6^ Department of Medical Biology, Medical University of Plovdiv, 4000 Plovdiv, Bulgaria; ^7^ Technological Centre of Emergency Medicine, 4000 Plovdiv, Bulgaria; ^8^ Department of General and Clinical Pathology, Medical University of Plovdiv, 4000 Plovdiv, Bulgaria

**Keywords:** Notum, glypicans, colorectal carcinogenesis, predictable animal models, WNT-pathway

## Abstract

The connection between colorectal cancer (CRC) and Wnt signaling pathway activation is well known, but full elucidation of the underlying regulation of the Wnt/β-catenin pathway and its biological functions in CRC pathogenesis is still needed. Here, the azoxymethane/dextran sulfate sodium salt (AOM/DSS) murine model has been used as an experimental platform able to mimic human sporadic CRC development with predictable timing. We performed genome-wide expression profiling of AOM/DSS-induced tumors and normal colon mucosa to identify potential novel CRC biomarkers. Remarkably, the enhanced expression of Notum, a conserved feedback antagonist of Wnt, was observed in tumors along with alterations in Glypican-1 and Glypican-3 levels. These findings were confirmed in a set of human CRC samples. Here, we provide the first demonstration of significant changes in Notum and glypicans gene expression during CRC development and present evidence to suggest them as potential new biomarkers of CRC pathogenesis.

## INTRODUCTION

Colorectal cancer (CRC) is the third most common neoplastic disease worldwide and is the second leading cause of cancer death in the Western world [1]. It develops via a multistage process that involves the accumulation of genetic and epigenetic alterations. Wnt signaling pathway activation and mutations in the KRAS and APC genes in the majority of colorectal cancers have been demonstrated [2, 3]. However, little is known regarding the fine regulation of the Wnt/β-catenin pathway or its biological functions that might be involved in the pathogenesis of CRC. Recent studies have focused on the activity of Wnt signals, showing that they are tightly controlled by various extracellular molecules. Kakugawa and colleagues recently added greatly to our understanding of Wnt signaling and the central role of Notum in the regulation of this pathway [4]. Notum is a negative feedback regulator of Wnt. Initial studies in fruit flies suggested that it could act as a phospholipase enzyme able to cleave the link between the membrane and glypicans (GPCs), a family of heparan sulfate proteoglycans that are linked to the outer leaflet of the plasma membrane by a glycosylphosphatidylinositol (GPI) anchor and that complex with Wnt [5]. However, Kakugawa et al. recently unveiled that Notum might be considered as a hydrolase enzyme which removes the acyl group from Wnt, thereby rendering the protein inactive. The acquisition or loss of the acyl group from palmitoleic acid can ably control the activation of Wnt signals [4]. Although Hedgehog is another signaling molecule whose activity is modified by lipids, it has been demonstrated that, unlike Wnt, Hedgehog is not a substrate for Notum and that, in flies, Notum does not interact with Hedgehog signaling [4]. It has been also shown that Notum contains binding sites for polysaccharides such as GPCs sugar chains, inviting speculation that GPCs bring together Notum and Wnt — thus modulating the enzymatic interaction of Notum with Wnt, rather than acting as a substrate for Notum to cleave GPI anchors [4].

In humans, colon carcinogenesis is a long, chronic process that is thought to occur over 10 to 20 years [6]. Experimental models that mimic the disease in rodents by chemical induction in less than a few months provide a means to understand the molecular alterations that arise in human CRC [7]. The well-established azoxymethane/dextran sodium sulfate (AOM/DSS) mouse model is a suitable platform to study the most dramatic molecular and signaling changes that occur during different phases of CRC development. Although the model does not progress to metastasis, as shown by studies extending until 20–30 weeks from tumor induction, it mimics quite well many of the steps found in human sporadic CRC progression [8]. Indeed, AOM/DSS-induced tumors share many histopathological and genetic characteristics with sporadic human colon tumors [9]. Furthermore, a number of studies have shown that the AOM/DSS model is a reliable tool for the investigation of not only the most advanced phases of colorectal carcinogenesis but also the earliest events underlying CRC initiation. At an early stage, single aberrant crypts appear in the colonic mucosa, which are hard to evaluate in corresponding human pathological specimens [10].

It could be an optimal experimental platform for the investigation of new biomarkers, also through advanced approaches. In this view, the analysis of the genomic instability resulting in copy number aberrations through massive parallel sequencing, as already demonstrated for prostate cancer [11] or the detection of tumor-derived circulating cell-free DNA in plasma samples [12] could represent new possibilities for the detection and monitoring of CRC to investigate in the AOM/DSS murine model.

Here, we performed a genome-wide expression profiling of colon mucosa to identify novel potential biomarkers that could be related to tumor initiation and progression after colon cancer induction via AOM and DSS in mice.

Interestingly, in addition to the preeminent activation of the Wnt/β-catenin pathway, we observed the enhanced expression of genes that encode Wnt antagonists, which might set up a negative feedback response to activated Wnt/β-catenin signaling in CRC. In particular, we found that the *NOTUM* gene was significantly up-regulated and that two heparan sulfate proteoglycans, Glypican-1 (*GPC1*) and Glypican-3 (*GPC3*), which are under Notum control and act as competitive inhibitors of Wnt [13], were up- and down-regulated, respectively. Our preclinical results as well as the recent study of Kakugawa et al. [4] and the comments of Nusse [14], describing the mechanism by which Wnt signals can be down-regulated by the extracellular enzyme Notum in Drosophila, prompted us to investigate Notum and glypican levels in a set of human CRC samples. Strikingly, we found a significant alteration of these Wnt pathway molecular mediators in human colorectal adenocarcinomas with respect to normal mucosa.

Taken together, our results provide the first demonstration of a perturbed expression in the *NOTUM*, *GPC1* and *GPC3* genes in the context of colorectal carcinogenesis. Additionally, we show a significant correlation between the expression levels of these molecules and that of β-catenin, suggesting their role as novel biomarkers in advanced CRC.

## RESULTS

### AOM and DSS combination is effective in inducing colon cancer

**Figure 1:**
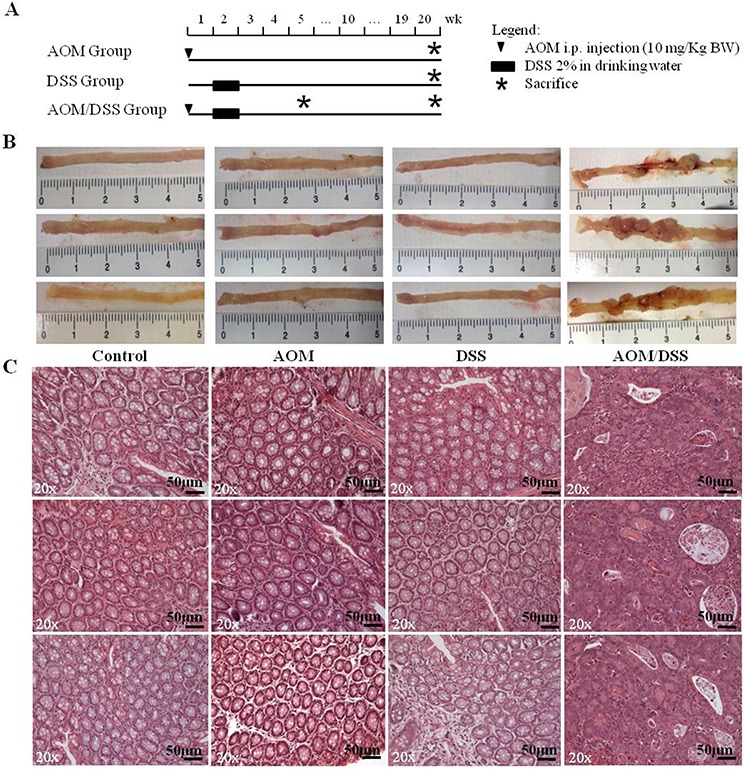
Experimental procedure and macroscopic and histological observation of the AOM/DSS murine model **A.** Schematic experimental procedure for groups treated with AOM-alone and/or DSS. Control group (untreated littermate controls) not represented. **B.** Macroscopic observation of the distal regions of colons from control, AOM-, DSS- and AOM/DSS-treated mice at the end of the 20^th^ week (only 3 of 6 animals per group are shown). Evident macroscopic lesions detectable only in AOM/DSS-treated colons. **C.** Hematoxylin/eosin staining of tumors and normal colons. Colon mucosae of AOM-only and DSS-only treated mice show the same histological characteristics of the control group. Adenocarcinomas with a high degree of dysplasia are detectable in AOM/DSS-treated mice. 20x original magnification. Scale bar, 50 μm.

Twenty weeks after AOM/DSS treatment (Figure 1A), the development of adenocarcinomas in the distal colon of mice was visually evident. Mice treated with AOM/DSS showed engrossed intestines with polypoid tumors, whereas mice that received AOM or DSS alone displayed macroscopically normal colons that were indistinguishable from those of untreated mice (Figure 1B).

All AOM/DSS-treated mice developed tumors (100% incidence) and the mean number of total tumors/mouse was 6.8 ± 2.7 (SD, standard deviation). After being isolated from the AOM/DSS treated mice, all lesions were analysed and confirmed to be adenocarcinomas (Figure 1C). These mouse lesions were histopathologically equivalent to human colorectal adenocarcinomas. They corresponded to well-differentiated enteroid adenocarcinomas and presented large numbers of flat and polypoid malignant tumors that were characterized by irregular, complex glands, an increased nucleus-to-cytoplasm ratio and marked losses of polarity and desmoplasia. The AOM-treated mucosa was perfectly comparable with the normal mucosa of untreated mice, whereas the DSS-treated mucosa was characterized by complete epithelial regeneration at 20 weeks after inflammatory stimulation (Figure 1C).

An additional group of animals, which were treated according to the AOM/DSS protocol, was sacrificed 5 weeks after treatment, and colon samples were collected for the immunohistochemistry of early stage CRC development. All samples presented 3–5 aberrant crypt foci (ACF), the earliest histopathological manifestations of colon lesions, which were characterized by clusters of abnormal tube-like glands in the lining of the colon and 1–3 low dysplastic microadenomas with sizes of less than 1 mm.

The macroscopic observation and histopathological analyses were made independently by two observers masked with respect to the treatment group and confirmed that the AOM/DSS model reliably reproduces, within a predictable time line, colorectal lesions distinctive of human CRC development, as reported in previous studies [7, 9]. On this basis, we investigated the transcriptional profile of advanced adenocarcinomas.

### Gene expression profile via microarray analysis

RNA from colon adenocarcinomas (AOM/DSS-treated) was analysed using MouseWG-6 v2.0 Expression BeadChips and compared with normal mucosae (untreated controls), AOM-only mucosae and DSS-only treated mucosae of mice euthanised on the 20th week.

**Figure 2:**
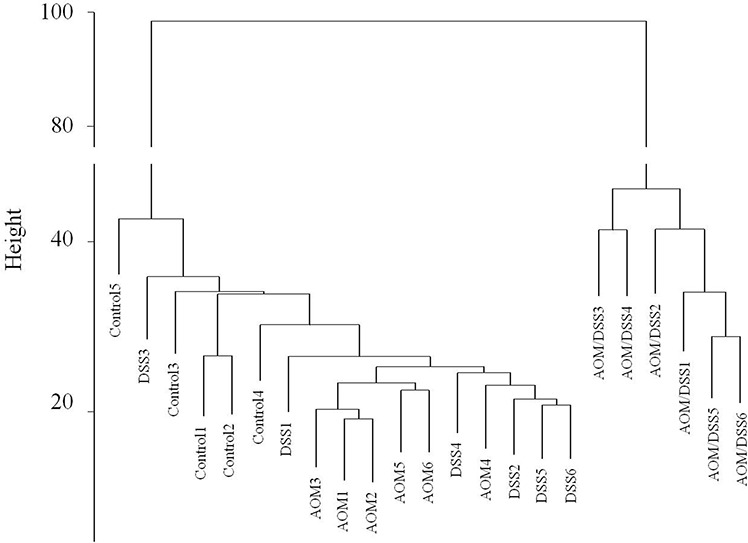
Hierarchical clustering of gene expression data Hierarchical clustering was generated by R hclust function, using Euclidean distance and average linkage as metrics.

The hierarchical clustering of array expression data showed a clear distinction between AOM/DSS-treated animals and the other groups (Figure 2). In addition, the AOM- or DSS-only treatments did not significantly affect the transcriptome, as their data clustered together with those of the untreated animals.

Linear model analysis (BH corrected *p*-value ≤ 0.05 |log_2_FoldChange| ≥ 1) of the 45280 probes present in the MouseWG-6 v2.0 Expression BeadChips, detected 2036 probes as differentially expressed in the adenocarcinoma group, 36 probes in DSS-mucosa and 44 probes in the AOM-mucosa group. The complete list of the gene probes and their expression levels, as determined by statistical analysis, is provided in Supplemental Table 1. Qiagen's Ingenuity Pathway Analysis version 7 program (IPA7, http://www.ingenuity.com) was applied to all 2036 probes (1502 genes) that were differentially expressed in the adenocarcinoma group.

Amongst the 50 most up-regulated and the 50 most down-regulated genes in adenocarcinomas, it is worth noting that several Paneth cell-specific genes, including those that code for defensins (*DEFA5, DEFA-RS2, DEFA4*) [15], secretory phospholipase A2 (*PLA2G2A*), frizzled-5 (*FZD5*) and matrix metallopeptidases (*MMPs*), were differentially expressed. In addition to the expected up-regulation of *AXIN2*, which is widely accepted to be an important downstream effector of the Wnt signaling cascade [16], a high number of other important targets and members of the Wnt signaling pathway were differentially expressed in adenocarcinoma. They included *SOX4* (SRY (sex determining region Y-box 4), *PLA2G2A* (phospholipase A2, group IIA), *CCND1* (cyclin D1), *WNT6* (wingless-type MMTV integration site family, member 6), *WIF1* (WNT inhibitory factor 1), *CD44, DKK3* (dickkopf WNT signaling pathway inhibitor 3), *TCF4* (transcription factor 4), *SFRP* (secreted frizzled-related protein), *FZL5* (frizzled-5), *LGR5* (leucine rich repeat containing G protein coupled receptor 5) and *EPHB2* (Eph receptor B2), as shown in Table 1 and Supplemental Table 1. Other interesting regulatory genes of the Wnt, Hh (Hedgehog), and BMP (Bone Morphogenetic Proteins) pathways were found to be up-regulated, such as *NOTUM* (log_2_ F*C* = 4.3) and *GPC1* (Glypican-1) (log_2_ F*C* = 4.3), or down-regulated, such as *GPC3* (Glypican-3) (log_2_ FC=−1.15). *KRT23* (keratin 23), which was recently identified as a putative immunologic target for colon cancer prevention [17], was also significantly up-regulated in the tumors.

**Table 1:** Top up and down regulated genes in adenocarcinoma, AOM- and DSS-treated colon mucosae

Accession number	Gene name	Gene description	log2FC	log2 average expression	BH corrected *p*-value	Experimental group
13240	Defa6	defensin, alpha, 6	6.78	9.44	5.95E-22	adenocarcinoma
18780	Pla2g2a	phospholipase A2, group IIA (platelets, synovial fluid)	6.68	10.19	3.28E-22	adenocarcinoma
100503970	AY761185	defensin related…	6.61	9.37	2.36E-20	adenocarcinoma
18946	Pnliprp1	pancreatic lipase-related protein 1	6.40	9.94	3.28E-22	adenocarcinoma
13222	Defa-rs2	defensin, alpha, 3	6.04	9.41	2.60E-18	adenocarcinoma
13238	Defa4	defensin, alpha, 4	5.72	9.26	5.20E-17	adenocarcinoma
16673	Krt36	keratin 36	5.25	9.97	4.66E-19	adenocarcinoma
17384	Mmp10	matrix metallopeptidase 10 (stromelysin 2)	5.22	9.59	5.87E-17	adenocarcinoma
94214	Spock2	sparc/osteonectin	5.11	9.33	4.86E-20	adenocarcinoma
17386	Mmp13	matrix metallopeptidase 13 (collagenase 3)	5.02	10.19	1.10E-16	adenocarcinoma
**77583**	**Notum**	**Notum pectinacetylesterase homolog (Drosophila)**	**5.00**	**9.21**	**1.04E-16**	**adenocarcinoma**
94179	Krt23	keratin 23 (histone deacetylase inducible)	4.78	10.49	4.48E-17	adenocarcinoma
17082	Il1rl1	interleukin 1 receptor-like 1	4.75	9.35	1.07E-21	adenocarcinoma
13218	Defa-rs1	defensin, alpha, related sequence 1	4.72	9.12	1.92E-17	adenocarcinoma
230810	Slc30a2	solute carrier family 30 (zinc transporter), member 2	4.69	9.02	3.28E-22	adenocarcinoma
23966	Odz4	teneurin transmembrane protein 4	4.67	9.30	4.55E-20	adenocarcinoma
213948	Atg9b	autophagy related 9B	4.67	9.14	8.28E-20	adenocarcinoma
18791	Plat	plasminogen activator, tissue	4.65	9.86	4.51E-17	adenocarcinoma
18947	Pnliprp2	pancreatic lipase-related protein	4.51	9.19	1.89E-17	adenocarcinoma
68713	Ifitm1	interferon induced transmembrane protein 1	4.48	11.11	3.43E-16	adenocarcinoma
99709	AI747448	autophagy related 9B	4.35	9.65	1.96E-13	adenocarcinoma
56753	Tacstd2	tumor-associated calcium signal transducer 2	4.35	9.15	5.20E-17	adenocarcinoma
20210	Saa3	serum amyloid A 3	4.34	9.17	3.51E-18	adenocarcinoma
**14733**	**Gpc1**	**glypican 1**	**4.31**	**11.01**	**1.03E-15**	**adenocarcinoma**
12006	Axin2	axin2	4.16	10.22	1.37E-15	adenocarcinoma
14038	Wfdc18	WAP four-disulfide core domain 18	4.16	9.48	1.82E-14	adenocarcinoma
13216	Defa1	defensin, alpha 1	4.08	8.92	1.13E-15	adenocarcinoma
11833	Aqp8	aquaporin 8	−4.96	13.31	1.05E-14	adenocarcinoma
12351	Car4	carbonic anhydrase 4	−4.70	12.54	2.68E-18	adenocarcinoma
192113	Atp12a	ATPase, H+/K+ transporting, nongastric, alpha polypeptide	−4.44	12.63	1.18E-12	adenocarcinoma
56857	Slc37a2	solute carrier family 37, member 2	−4.43	10.94	1.82E-14	adenocarcinoma
11537	Cfd	complement factor D	−4.20	11.50	5.17E-05	adenocarcinoma
20341	Selenbp1	selenium binding protein 1	−4.12	13.61	2.56E-16	adenocarcinoma
13170	Dbp	D site albumin promoter binding protein	−3.91	11.05	1.05E-08	adenocarcinoma
72082	Cyp2c55	cytochrome P450, family 2, subfamily c, polypeptide 55	−3.90	12.58	6.66E-12	adenocarcinoma
380997	Cyp2d12	cytochrome P450, family 2, subfamily d, polypeptide 12	−3.70	10.82	7.34E-17	adenocarcinoma
23919	Insl5	insulin-like 5	−3.68	11.12	1.77E-15	adenocarcinoma
20500	Slc13a2	solute carrier family 13, member 2	−3.68	12.17	7.29E-13	adenocarcinoma
72273	2210404O07Rik	small integral membrane protein	−3.66	12.49	1.92E-14	adenocarcinoma
216225	Slc5a8	solute carrier family 5 (iodide transporter), member 8	−3.56	12.81	7.78E-13	adenocarcinoma
21818	Tgm3	transglutaminase 3, E polypeptide	−3.47	11.03	2.63E-13	adenocarcinoma
382097	Gm1123	predicted gene 1123	−3.45	11.67	3.95E-13	adenocarcinoma
20342	Selenbp2	selenium binding protein 2	−3.43	11.25	1.59E-14	adenocarcinoma
11522	Adh1	alcohol dehydrogenase 1 (class I)	−3.37	13.98	9.73E-12	adenocarcinoma
20363	Sepp1	selenoprotein P, plasma, 1	−3.34	12.83	3.79E-14	adenocarcinoma
68416	Sycn	syncollin	−3.34	14.97	9.25E-12	adenocarcinoma
64385	Cyp4f14	cytochrome P450, family 4, subfamily f, polypeptide 14	−3.29	12.48	1.58E-12	adenocarcinoma
234669	Ces2b	carboxyesterase 2B	−3.24	11.00	6.07E-15	adenocarcinoma
13105	Cyp2d9	cytochrome P450, family 2, subfamily d, polypeptide 9	−3.22	11.05	2.32E-12	adenocarcinoma
20887	Sult1a1	sulfotransferase family 1A, phenol-preferring, member 1	−3.19	12.30	1.78E-15	adenocarcinoma
13346	Des	desmin	−3.16	11.60	1.53E-10	adenocarcinoma
13113	Cyp3a13	cytochrome P450, family 3, subfamily a, polypeptide 13	−3.13	11.30	1.46E-14	adenocarcinoma
53315	Sult1d1	sulfotransferase family 1D, member 1	−3.12	11.12	1.28E-14	adenocarcinoma
393082	Mettl7a2	methyltransferase like 7A2	−3.11	9.63	4.52E-10	adenocarcinoma
13101	Cyp2d10	cytochrome P450, family 2, subfamily d, polypeptide 10	−3.10	11.64	5.38E-14	adenocarcinoma
56643	Slc15a1	solute carrier family 15 (oligopeptide transporter), member 1	−3.02	10.38	5.60E-17	adenocarcinoma
**14734**	**Gpc3**	**glypican-3**	**-1.15**	**8.94**	**1.14E-08**	**adenocarcinoma**
93695	Gpnmb	glycoprotein (transmembrane) nmb	1.81	10.01	1.26E-05	AOM
12653	Chgb	chromogranin B	1.57	10.46	2.95E-06	AOM
15360	Hmgcs2	3-hydroxy-3-methylglutaryl-Coenzyme A synthase 2	1.49	12.65	3.87E-02	AOM
75646	Rai14	retinoic acid induced 14	1.37	7.32	2.45E-02	AOM
76459	Car12	carbonic anyhydrase 12	1.28	10.38	7.15E-04	AOM
21990	Tph1	tryptophan hydroxylase 1	1.12	8.88	2.82E-07	AOM
26914	H2afy	H2A histone family, member Y	−2.61	8.71	3.08E-15	AOM
13170	Dbp	D site albumin promoter binding protein	−2.56	11.05	5.15E-04	AOM
225742	St8sia5	ST8 alpha-N-acetyl-neuraminide alpha-2,8-sialyltransferase 5	−2.3	9.07	1.75E-07	AOM
108017	Fxyd4	FXYD domain-containing ion transport regulator 4	−2.22	10.23	1.04E-05	AOM
393082	Mettl7a2	methyltransferase like 7A2	−1.83	9.63	1.93E-04	AOM
192113	Atp12a	ATPase, H+/K+ transporting, nongastric, alpha polypeptide	−1.66	12.63	8.85E-04	AOM
229599	Gm129	circadian associated repressor of transcription	−1.65	8.93	7.08E-06	AOM
17748	Mt1	metallothionein 1	−1.6	14.81	3.45E-06	AOM
380997	Cyp2d12	cytochrome P450, family 2, subfamily d, polypeptide 12	−1.52	10.82	1.75E-07	AOM
66184	Rps4y2	ribosomal protein S4-like	−1.52	8.43	1.55E-09	AOM
17829	Muc1	mucin 1, transmembrane	−1.4	9.57	1.31E-04	AOM
67133	Gp2	glycoprotein 2 (zymogen granule membrane)	−1.27	10.00	3.27E-03	AOM
20208	Saa1	serum amyloid A 1	−1.22	11.16	3.60E-03	AOM
12346	Car1	carbonic anhydrase 1	−1.21	14.02	1.05E-02	AOM
56857	Slc37a2	solute carrier family 37, member 2	−1.21	10.94	2.07E-03	AOM
13105	Cyp2d9	cytochrome P450, family 2, subfamily d, polypeptide 9	−1.2	11.05	1.29E-03	AOM
67204	Eif2s2	eukaryotic translation initiation factor 2, subunit 2 (beta)	−1.19	11.80	7.47E-06	AOM
27409	Abcg5	ATP-binding cassette, sub-family G (WHITE), member 5	−1.17	11.49	4.01E-03	AOM
13034	Ctse	cathepsin E	−1.15	11.54	8.49E-04	AOM
53376	Usp2	ubiquitin specific peptidase 2	−1.14	9.19	6.67E-05	AOM
207952	Klhl25	kelch-like 25	−1.06	8.24	6.88E-07	AOM
208715	Hmgcs1	3-hydroxy-3-methylglutaryl-Coenzyme A synthase 1	−1.03	14.25	6.52E-03	AOM
20692	Sparc	secreted acidic cysteine rich glycoprotein	−1.01	10.58	7.42E-03	AOM
67080	1700019D03Rik	RIKEN cDNA 1700019D03 gene	−1	11.38	1.40E-03	AOM
66660	Sltm	SAFB-like, transcription modulator	2.36	8.82	3.75E-02	DSS
12653	Chgb	chromogranin B	1.6	10.46	2.62E-06	DSS
75646	Rai14	retinoic acid induced 14	1.4	7.32	2.57E-02	DSS
18030	Nfil3	nuclear factor, interleukin 3, regulated	1.29	9.42	8.39E-05	DSS
21990	Tph1	tryptophan hydroxylase 1	1.24	8.88	5.26E-08	DSS
11551	Adra2a	adrenergic receptor, alpha 2a	1.23	10.69	7.68E-03	DSS
76459	Car12	carbonic anyhydrase 12	1.16	10.38	1.94E-03	DSS
104158	Ces1d	carboxylesterase 1D	1.13	11.21	3.84E-02	DSS
242705	E2f2	E2F transcription factor 2	1.04	9.05	6.36E-05	DSS
66811	Duoxa2	dual oxidase maturation factor 2	1	9.91	1.01E-02	DSS
13170	Dbp	D site albumin promoter binding protein	−3.68	11.05	4.09E-06	DSS
26914	H2afy	H2A histone family, member Y	−2.57	8.71	3.93E-15	DSS
229599	Gm129	circadian associated repressor of transcription	−1.87	8.93	1.18E-06	DSS
225742	St8sia5	ST8 alpha-N-acetyl-neuraminide alpha-2,8-sialyltransferase 5	−1.65	9.07	4.46E-05	DSS
393082	Mettl7a2	methyltransferase like 7A2	−1.64	9.63	9.21E-04	DSS
66184	Rps4y2	ribosomal protein S4-like	−1.44	8.43	4.32E-09	DSS
108017	Fxyd4	FXYD domain-containing ion transport regulator 4	−1.42	10.23	2.94E-03	DSS
21685	Tef	thyrotroph embryonic factor	−1.34	9.95	2.17E-03	DSS
217166	Nr1d1	nuclear receptor subfamily 1, group D, member 1	−1.2	10.00	2.45E-02	DSS
53376	Usp2	ubiquitin specific peptidase 2	−1.17	9.19	6.03E-05	DSS
12346	Car1	carbonic anhydrase 1	−1.12	14.02	2.45E-02	DSS
17829	Muc1	mucin 1, transmembrane	−1.1	9.57	1.94E-03	DSS
13034	Ctse	cathepsin E	−1.08	11.54	1.77E-03	DSS
20692	Sparc	secreted acidic cysteine rich glycoprotein	−1.07	10.58	5.73E-03	DSS
192113	Atp12a	ATPase, H+/K+ transporting, nongastric, alpha polypeptide	−1.05	12.63	3.84E-02	DSS
380997	Cyp2d12	cytochrome P450, family 2, subfamily d, polypeptide 12	−1.04	10.82	6.48E-05	DSS
56857	Slc37a2	solute carrier family 37, member 2	−1.04	10.94	8.74E-03	DSS

The fourth column shows the Log2FC of adenocarcinoma, AOM-treated mucosa and DSS-treated mucosa, respectively, vs normal mucosa. The significance level is indicated by a corrected *p*-value.

Interestingly, the majority of down-regulated genes in the tumors were cell membrane transporters that are involved in the movement of ions or small substrates across the intestinal membrane, such as *AQP8* (aquaporin 8), a marker of normal proliferating colonic epithelial cells that is involved in fluid transport in the colon; *CAR4* (carbonic anhydrase 4), which has established roles in bicarbonate production and secretion in the intestine; *ATP12A* (ATPase, H+/K+ transporting), which is responsible for potassium absorption; *SLC37A2* (solute carrier family 37 (glucose-6-phosphate transporter) member-2) and *SELENBP1* (selenium-binding protein).

The AOM-only treated and DSS-only treated groups displayed a small set of altered genes, with very low log2-FC values, compared with the AOM/DSS treated colon mucosa. Among the altered genes, the common up-/down-regulated ones, which were found in both experimental groups, were genes that are mainly involved in (i) gene expression regulation, such as *H2AFY* (H2A histone family, member Y), *DBP* (the D site of albumin promoter binding protein), *EIF2S2* (eukaryotic translation initiation factor 2, subunit 2), *GM129* (circadian associated repressor of transcription), *RPS4Y2* (ribosomal protein S4, Y-linked 2); (ii) membrane receptors and transporters, such as *GPNMB* (glycoprotein NMB), *FXYD4* (frizzled family receptor 4), *MUC1* (mucin 1), *ATP12A* (ATPase, H+/K+ transporting), *SLC37A2* (solute carrier family 37 (glucose-6-phosphate transporter), member-2), *CTS-E* (cathepsin E), *SPARC* (secreted protein, acidic, cysteine-rich); and (iii) metabolism, such as *HMGCS2* (3-hydroxy-3-methylglutaryl-CoA synthase-2), *TPH1* (tryptophan hydroxylase 1); and *SAA1* (serum amyloid A1). It is also worth noting that genes coding for detoxification enzymes were altered in both groups: in particular, *MT1* (metallothionein 1), *CYP2D12* and *CYP2D9* (cytochrome P450) were found to be down-regulated in AOM-treated mice, whereas *CES1D* (carboxylesterase 1D) and *CYP2DL2* (cytochrome P450) were up- and down-regulated, respectively, in DSS-treated mice. Both groups showed no alterations in inflammatory genes.

**Figure 3:**
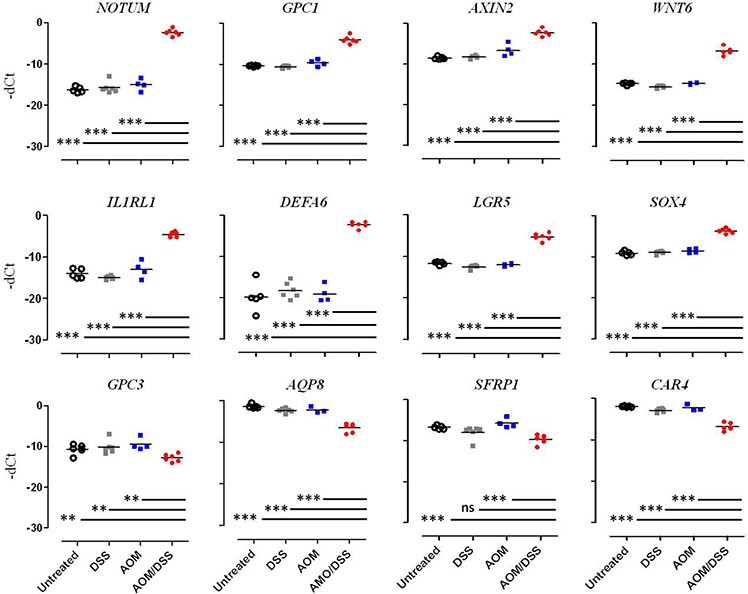
Multi-Gene qPCR validation of differential gene expression Gene expression levels were measured in the four different conditions: Untreated (white circle), DSS (grey square), AOM (blue square) and AOM/DSS (red circle). The results are expressed as Delta C_T_ values between the C_T_ value of the gene of interest and the C_T_ value of β2-microglobulin. Each dot represents the evaluation of the gene levels in a single mouse. Statistically significant differences were calculated using Student's *t*-test: ***p < 0.0005; **p < 0.0078.

Single qPCR, which was performed on a subset of genes selected among that mainly dysregulated genes or involved in Wnt pathway control, confirmed the microarray analysis results: *NOTUM, GPC1*, *AXIN2*, *WNT6, IL1RL1, DEFA-6, LGR5,* and *SOX4* were found to be up-regulated, whereas *GPC3, AQP8, SFRP1,* and *CAR4* were down-regulated (Figure 3).

### Two main pathways are altered in adenocarcinomas: the LPS/IL-1-mediated inhibition of RXR function and the Wnt/β-catenin signaling pathway

Our microarray data set was analysed using IPA7 to discern whether specific biological pathways or functional gene groups were differentially affected in tumors and which of these pathways were still altered in AOM-only and DSS-only treated mucosa after a period of mucosal regeneration.

**Figure 4:**
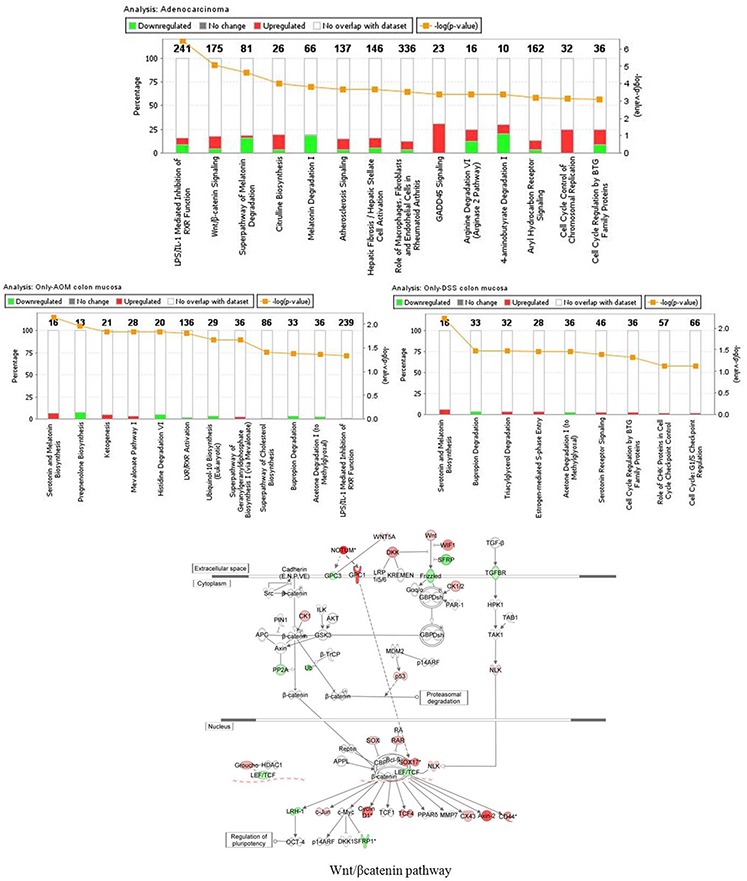
Enriched canonical pathways of the differentially expressed genes as determined by Ingenuity Pathway Analysis (IPA) The significance of canonical pathways was determined by IPA's default threshold [−log(*p*-value)]> 3 for adenocarcinoma and [−log (*p*-value)] > 1.3 for colon mucosa of AOM-only and DSS-only treated mice. *P*-value calculated by Fisher's exact test. The associated gene number above each column represents the number of differentially expressed genes that were involved in the respective canonical pathways. The percentage of genes that were up- or down-regulated is represented in red or green, respectively. In the lower figure, the Wnt/β-catenin pathway has been added and shows the IPA overlay of analysis in adenocarcinoma (up-regulated genes in red, down-regulated genes in green). Direct or indirect interactions are shown by complete or dashed lines, respectively.

The results of the gene ontology analysis showed that the top 3 canonical pathways altered in adenocarcinomas were the LPS/IL-1 mediated inhibition of RXR function, Wnt/β-catenin signaling and the super-pathway of melatonin degradation (Figure 4).

The metabolic super-pathway of melatonin degradation was the pathway with the majority of genes being significantly down-regulated. Several mechanisms by which melatonin controls colon cancer have been proposed and involve the inhibition of tumor angiogenesis, maintenance of the intracellular level of glutathione, and modulation of mitotic and apoptotic processes [18]. However, further studies and controlled clinical trials are still needed in order to establish melatonin's role in cancer treatment more concretely.

We focused our attention on the top 2 significantly dysregulated pathways both of which enclosed the highest number of genes (Supplemental Table 2).

An examination of the LPS/IL-1-mediated inhibition of the RXR function signaling pathway revealed the strong transcriptional up-regulation of several genes that are linked to inflammation and injury response, including *IL1β* (interleukin 1 beta), *IL1RL1* (IL1 receptor-like 1), *TNF* (tumor necrosis factor), *TNFRSF11B* (TNF receptor superfamily, member 11b), *APOC4* (apolipoprotein C-IV), *APOE* (apolipoprotein E), and *GST* (glutathione S-transferase), but the down-regulation of *PPARGC1A/B* (peroxisome proliferator-activated receptor gamma, coactivator 1 alpha/beta). Reductions in the expression of transport proteins, such as *ABCG5/8* (ATP-binding cassette, sub-family G, member 5/8) and *ABCC3* (ATP-binding cassette, sub-family C, member 3), and metabolizing enzymes, such as *GSTT1* (glutathione S-transferase theta 1), *MGST1/3* (microsomal glutathione S-transferase 1/3) and *CYP2C9* (cytochrome P450, family 2, subfamily C, polypeptide 9), were also detected. The decreased expression of these genes has been recently shown to cause impaired metabolism, transport and/or biosynthesis of lipids, cholesterol and xenobiotics [19–21].

As expected, the Wnt/β-catenin signaling pathway was found to be largely involved in tumor progression. As shown in Figure 4 and Supplemental Table 1, the major up-regulated genes were the ligands *WNT5A, WNT6,* and *WNT10A*; the Wnt receptor *FZD10,* the scaffolding protein *AXIN2*, which is essential for β-catenin degradation; the Wnt inhibitory proteins (*WIF1, DKK3)*; *TCF4*, the β-catenin transcriptional partner and the Nemo-like kinase (*NLK)*, which leads to low LEF/TCF/β-catenin complex binding to DNA and LEF1/TCF4 degradation [22].

Other genes, whose functions have been shown to correlate with the Wnt/β-catenin signaling pathway, were *LGR5, EPHB2, EPHB4, EPHB6, NOTUM, GPC1* and *GPC3* [22, 23]. Interestingly, only a few altered genes were found in the AOM-only and DSS-only treated colon mucosae. Moreover, *NFKB* and *AKT* were equally detected to be hub genes of the most altered molecular pathways in both experimental groups, even without the direct deregulation of their expression level (data not shown). In fact, only the residual activity of the *NFKB* inflammatory response pathway and the antiapoptotic activity of *AKT* appeared to persist during the regeneration of the colon mucosa, which occurred after either the AOM-only or DSS-only treatments.

### Correlation of Notum overexpression with intracellular β-catenin staining and changes of glypicans tissue distribution

**Figure 5:**
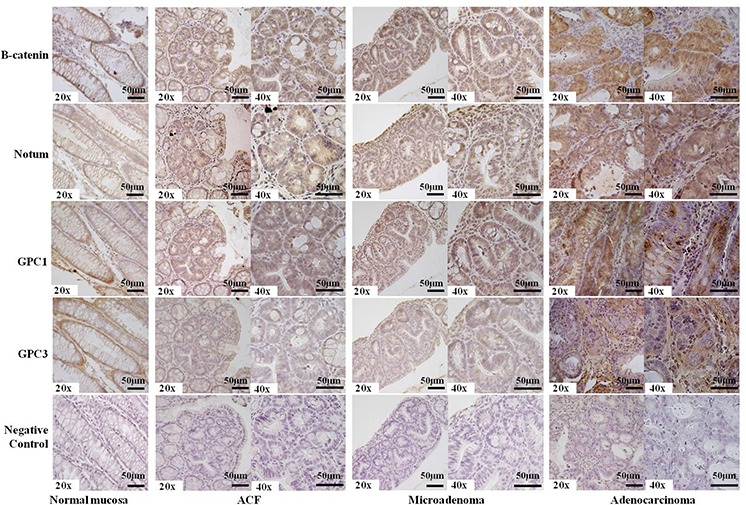
Representative results of immunostaining for β-catenin, Notum, Glypican-1, Glypican-3 in FFPE mouse tissue sections Membrane-bound β-catenin, Notum and Glypican-1 were observed in normal colon mucosa, whereas dysplastic ACF (Aberrant Crypt Foci), microadenomas, and adenocarcinomas exhibited more intense (++) staining (nuclear or cytoplasmic). Glypican-3 staining was less intense (+) than Glypican-1 staining (++), and the adenocarcinoma showed a negative signal (−) with respect to nuclear or cytoplasmic compartments along with more intense (++) extracellular staining. Incubation with a primary antibody was omitted in the negative control. Sections were counterstained with hematoxylin: 20x and 40x original magnification. Scale bar, 50 μm.

In the context of the Wnt/β-catenin pathway, we focused our attention on the regulation of Notum expression in CRC murine lesions as Armadillo, an ortholog of human and murine β-catenin, is known to regulate Drosophila Notum [23]. We performed immunohistochemistry on advanced adenocarcinomas and early lesions of animals sacrificed 5 weeks after tumor induction (Figure 5). Intracellular β-catenin (nuclear and cytoplasmic staining) was observed in all CRC FFPE sections, whereas membrane-bound β-catenin was detected only in normal colon mucosa. Dysplastic ACF exhibited intense intracellular β-catenin staining, which is consistent with previous reports [7, 9], and the same pattern was observed for microadenomas, adenomas and adenocarcinomas.

Notum overexpression was associated with intracellular β-catenin localization (100% of cases) and also observed in early lesions. Glypican-1 staining showed the same type of Notum staining positivity, although a mild cytoplasmic signal was also observed in early lesions. Positive staining for Glypican-1 was observed in 90% of all studied ACFs (21 positive of 24 analysed), whereas Glypican-3 exhibited evident membrane staining in normal colon mucosa and a negative signal in all (100%) dysplastic colon crypts. Similarly, no membrane staining was present in the most advanced tumors. However, we observed extracellular staining in these tumors that is indicative of a marked release of Glypican-3 from the cell membrane (Figure 5).

### Notum and glypicans gene expression and protein localization in human CRC samples

**Figure 6:**
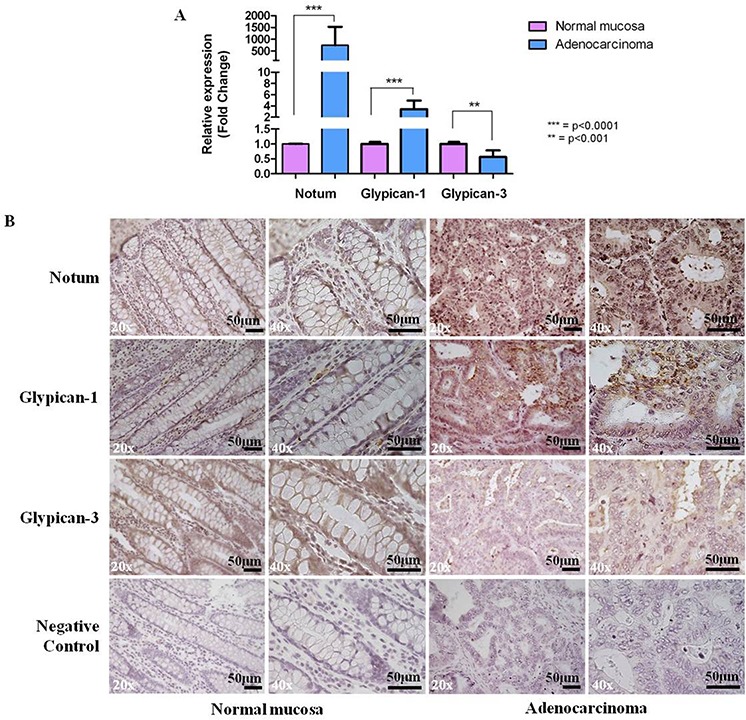
A. qPCR analysis of *NOTUM*, *GPC1* and *GPC3* differential gene expression in human samples Gene expression levels were measured in human colorectal adenocarcinomas with respect to normal colon mucosae, and results are expressed as fold changes, considering the C_T_ value of the gene of interest and the C_T_ value of β-actin. Data are represented as mean +/− SD). Statistically significant differences were calculated using Student's *t*-test: ***p < 0.0001; **p < 0.001. **B.** Representative results of immunostaining for Notum, Glypican-1 and Glypican-3 in human tissue sections. Strong staining (++) in CRC cases for Notum and Glypican-1; weak staining (−) in CRC cases for glypican-3. Incubation with a primary antibody was omitted in the negative control. Sections were counterstained with hematoxylin: 20x and 40x original magnification. Scale bar, 50 μm.

To investigate the eventual correspondence between our observations in the CRC murine model and human colorectal carcinogenesis, we analysed the mRNA levels of *NOTUM*, *GPC1* and *GPC3* in 10 representative human tissue samples, including primary CRC specimens and normal distal colorectal mucosa from the same patient. In this pilot study, a statistically significant up-regulation of *NOTUM* and *GPC1* ( *p*-value < 0.0001) and down-regulation of *GPC3* ( *p*-value < 0.001) mRNA levels were observed in all tumor samples. The relative expression levels were determined with the ΔΔC_T_ method individually for each patient with the normal mucosa serving as the calibrator (Figure 6A). The expression level values ranged, respectively, from 66 to 4408 for *NOTUM* (median value 520.45), from 1.57 to 6.77 for *GPC1* (median value 2.76), and from 0.2 to 1.2 for *GPC3* (median value 0.69).

To assess Notum, Glypican-1 and Glypican-3 protein localization in human CRC samples, we performed immunohistochemical analysis on 10 tumor cases and 10 normal matched mucosa specimens. As previously observed in mouse specimens, the analysis revealed a strong increase of membrane/cytoplasmic staining in tumors for Notum and Glypican-1 in 100% (10/10) and 80% (8/10) of tumors, respectively, and a weak staining for Glypican-3 in 80% (8/10) (Figure 6B).

## DISCUSSION

The aim of our study was to identify novel genes that are specifically deregulated in colorectal cancer using a reliable preclinical platform represented by the chemically induced mouse model of sporadic CRC. The AOM/DSS model employed in this study (one injection of AOM, one cycle of DSS – “two step model”) is an acute inflammation-related model in which the initial inflammatory microenvironment is important to promote and accelerate the malignant progression which starts after the AOM administration. Nevertheless, the most advanced adenomas and adenocarcinomas lack the strong inflammatory background observed in other mouse models, which proceed through repeated cycles of DSS administration (one injection of AOM, three cycles of DSS). In that case, the inflammation becomes chronic and non-resolved [24], as seen in human IBD-related CRC where chronic inflammation always precedes and is directly linked to tumor formation [25, 26]. Changes in gene expression are extensive in those models, especially in genes involved in the inflammatory response and immune defence [27]. Conversely, tumors generated by our “two step” AOM/DSS treatment accurately recapitulate the pathogenesis observed in human sporadic noninflammatory CRC [28]. We observed that the molecular assessment of AOM/DSS-induced tumors is characterized by a mild inflammatory background with a number of genes linked to inflammation and innate immune response, as will be discussed in detail hereafter, but without alterations in the key inflammation pathway mediators, e.g., the *NFKB, IL-10, IL-6, TNFA* and *TGFβ* genes [29, 30].

Few data are available on the long-term adaptive response of colon mucosa with respect to an independent low genotoxic stress effect (such as a single dose of the mutagen AOM) or to mild inflammatory stress (a single cycle of DSS administration). Therefore, we decided to analyse the colon mucosa of mice treated with single AOM and DSS administrations, following a regeneration time of 20 weeks. We observed a very small number of altered genes in both AOM-only and DSS-only treated mice with respect to the normal mucosa of untreated mice (Table 1). We confirmed that both the AOM-only and DSS-only treated mice showed not only morphologically normal colon mucosa but also no molecular alterations approximately 20 weeks after the initial AOM or DSS stimulation (Figure 1). Indeed, hierarchical clustering of gene expression profiles (Figure 2) revealed reasonable overlap between the genes altered in the AOM and DSS mucosae compared with the normal mucosa of the control group, proving that almost complete colon mucosa regeneration can occur after both AOM and DSS treatments. We also confirmed that the carcinogen AOM minimally affects the molecular functions of the treated colon mucosa without a subsequent DSS-induced inflammatory phase in the AOM/DSS colorectal cancer model, which is in accordance with the findings of Tanaka et al. [8].

Gene expression profiling in the adenocarcinoma showed the differential expression of 2036 probes with a |log_2_ FC| ≥ 1, including 1092 (53.6%) that were up-regulated and 944 (46.4%) that were down-regulated. In general, we observed a significant variation in the expression of genes, particularly those related to cell growth, immune response, cellular transport and the regulation of morphology. Our data indicate that antimicrobial α-defensins, known as components of the innate immune system, are among the most up-regulated genes in adenocarcinomas. The presence of abnormally high defensins expression levels has been identified in a variety of human tumors [31]; however, their role in carcinogenesis requires further investigation. Other markers of Paneth cells, such as secretory phospholipase A2 (*PLA2G2A*), *MMP10* and *MMP13*, were also up-regulated, confirming that these cells are a cellular component of colon tumors, which is in agreement with previous reports [32, 33]. Interestingly, the presence of Paneth cells in colon cancers has been suggested to be a likely consequence of Wnt pathway activation [34].

We also reported the up-regulation of interleukin 1 receptor-like 1 (*IL1RL1*), interleukin-1 beta (*IL1β*) and other genes involved in the “LPS/IL1 mediated inhibition of RXR function”, which were all identified by IPA ontology analysis. The above observation is consistent with other data that report that the transgenic overexpression of *IL1β* in gastric mucosa is sufficient to induce gastric cancer in mice [35]. New findings have recently indicated that *IL1β* may promote colon tumor growth and invasion via the activation of cancer stem cell self-renewal and epithelial-mesenchymal transition (EMT) [36]

As expected, the second most dysregulated canonical pathway in adenocarcinoma was the Wnt/β-catenin pathway. Wnt target genes are varied and context-specific [37] because Wnt/β-catenin signaling controls proliferation, cell fate determination and differentiation in numerous developmental stages and in adult tissue homeostasis. Furthermore, Wnt signaling components are positively or negatively regulated by TCF/β-catenin [38–41].

Our results indicate that the Wnt-induced activation of *AXIN2* and *DKK3* as well as suppression of *FZD5*, secreted frizzled-related protein 1 (*SFRP1*) and transforming growth factor beta 3 (*TGFB3*) constituted negative feedback loops, which are able to dampen Wnt signaling. In addition, *IL1β* has been shown to indirectly activate β-catenin signaling by inducing canonical *WNT7B* expression and by inhibiting the expression of canonical Wnt antagonists [42]. Furthermore, we observed the up-regulation of *IL1β* and *IL1RL1*. We detected significant over-expression of *TCF4*, which is in accordance with Wnt-induced TCF/LEF gene expression and constitutes a positive feed-forward circuit that reinforces Wnt signaling. This feature has been observed elsewhere during colon carcinogenesis [43]. These various Wnt pathway self-regulatory loops are mostly used in a cell-specific modality, affording additional complexity to Wnt response amplitude and duration control.

Importantly, with regard to Wnt/β-catenin signal modulation, the results of our microarray analysis identified the overexpression of *NOTUM* and the alteration of *GPC1* and *GPC3* expression. There has been a recent renewed interest in the involvement of Notum and glypicans in the modulation of Wnt signaling. The secreted enzyme Notum has been found to inactivate Wnt by removing a lipid that is linked to the Wnt protein and that is required for the activation of Wnt receptor proteins [4]. Wnt signals also turn on the expression of the gene encoding Notum, leading to negative feedback regulation that intrinsically limits signaling. Originally discovered in fruit flies in screens for genes that interact with the Wnt protein Wingless, *NOTUM* gene encodes a secreted hydrolase, which has recently been identified in hepatocellular carcinoma with mutant β-catenin [44].

The Notum-enhanced expression in murine colorectal adenocarcinoma reflects its activation in the canonical Wnt/β-catenin pathway, implying the crucial involvement of Notum dysregulation in CRC pathogenesis. Our clinical findings in a set of human sporadic colorectal adenocarcinomas confirmed the transcriptional and protein up-regulation of Notum. This finding was in agreement with the over-expression of Notum observed in human primary colorectal and gastric cancers and their cell lines as shown in the expression database of the International Genomics Consortium (http://www.intgen.org/), where Notum overexpression is also reported in breast, lung, ovarian and endometrial cancers.

In addition, we observed the over-expression of *GPC1* and under-expression of *GPC3* in murine tumors. As the activity of NOTUM has been demonstrated to involve the release of GPI-anchored glypicans (analogous to Drosophila Dip and Dally) from the cell surface [45], we further investigated the involvement of such genes in the Wnt/β-catenin pathway in not only the tumors but also preneoplastic lesions detected in the colon of AOM/DSS-treated animals at 5 weeks after tumor induction. Immunohistochemistry confirmed the overexpression of Notum in 100% of the adenocarcinomas analysed. High levels of the protein were significantly associated with the intracellular (nuclear or cytoplasmic) accumulation of β-catenin (Figure 5). In contrast, the expression of the two glypicans followed a different trend. Glypican-3 exhibited marked membrane staining in normal colon mucosa, whereas a negative signal was observed in both early and late colon lesions. This finding provided further evidence supporting the functional link between Notum and Glypican-3. In fact, the use of a cellular system in which Glypican-3 stimulates Wnt signaling provided evidence that Notum can act as a negative functional regulator of this growth factor [46]. Glypican-3 has been shown to be an inhibitor of cell proliferation, and it can induce apoptosis in certain types of tumor cells [47]. Recent reports have indicated that Glypican-3 displays a tissue-specific pattern of expression during tumor progression. Glypican-3 expression is reduced in the progression of cancers that originate from GPC3-positive tissues, such as ovarian cancer [48] and mesothelioma [49]. Conversely, the expression of this glypican is up-regulated in hepatocellular carcinomas, although Glypican-3 is not expressed in the liver, suggesting that in this case it behaves as an oncofoetal protein [50]. Recent studies have also showed a down-modulation of Glypican-3 and an up-regulation of Glypican-1 gene expression in glioblastoma [51]. The molecular and immunohistochemical analysis performed in our human CRC set revealed very similar results, indicating the significant association of *GPC1* over-expression and *GPC3* under-expression in tumor lesions with respect to the normal colon mucosa (Figure 6).

Possible mechanisms at the basis of the transcriptional control of these two glypicans have been investigated. In several malignant tumors, including ovarian carcinoma, cholangiocarcinoma, mesothelioma, and breast cancer, *GPC3* is down-regulated as a result of hypermethylation of the *GPC3* promoter [48, 49, 52, 53]. Also, the involvement of miRNAs in postrascriptional control of *GPC3* has been shown in hepatocellular carcinomas [54].

With regard to *GPC1*, few data have been reported describing transcriptional modulation of the gene in different kinds of tumors, but without a functional explanation [55]. However, the existence of other overlapping levels of regulation, possibly at level of translation have been suggested; as such, the existence of translation-level regulation has been described in some genes involved in the biosynthesis of glycosaminoglycans and proteoglycans [56].

Here, we showed the inverse correlation between gene expression and protein distribution of Notum and Glypican-3. The progressive accumulation of Glypican-3 in the adenocarcinoma extracellular environment may be explained by the release of Glypican-3 from the cell membrane. Nevertheless, the possibility that this release, in turn, could be due to the enzymatic activity of Notum, which is contextually up-regulated during CRC development, has been overcome by the last findings of Kakugawa et al [4]. Other mechanisms mediated by sheddase (protease or heparanase) could be responsible of the cleavage of cell surface attached Glypican-3 although further studies are needed to clarify this point. Currently, the mechanism of Glypican-3 shedding into the extracellular space and sera is of general interest, and the possibility of using GPC3 as a serological marker in different cancer types is under investigation [57].

Less is known regarding Glypican-1, which, in our study, showed a very similar expression pattern to that of Notum in late lesions, although some positive staining was also observed in 90% of the analysed ACFs. This finding suggests that Glypican-1 is important but not essential for tumor initiation. Glypican-1 expression has also been found to be significantly increased in a large proportion of pancreatic tumors [58, 59]. However, the functional link between Notum and Glypican-1 would benefit from in-depth investigation.

Numerous genetic and functional studies performed in Drosophila, Xenopus, zebrafish and mammals have demonstrated that glypicans may regulate the signaling activity of Wnts, Hedgehog (Hh), BMPs and FGFs in a tissue-specific manner [60–62].

For example, the activation of the Hh pathway, which is deeply involved in CRC development, can be due to the removal of the functional inhibitory effect which is exerted by GPC3 through the competition of GPC3 with the receptor Patched for Hh binding [45]. Interestingly, no transcriptional dysregulation of the principal genes involved in Hh signaling pathway was observed in our CRC model.

In the particular case of Wnts, GPI-anchored glypicans have been proposed to stimulate signaling by facilitating and/or stabilizing the interaction between Wnts and their cell surface receptors [63]. However, glypicans can also act as competitive inhibitors of Wnt signaling when secreted into the extracellular environment [64]. On this basis, our data on Glypican-1 and Glypican-3 dysregulation are of high interest, as they can help depict a more comprehensive picture of Wnt signal modulation in the tumor microenvironment.

In summary, we have demonstrated the following for the first time. Notum is over-expressed in early and late lesions of the AOM/DSS murine model of sporadic CRC and in human colorectal adenocarcinomas. Notum expression levels are correlated to β-catenin abnormal distribution, indicating that Notum expression is associated with canonical Wnt signal modulation in CRC pathogenesis. Glypican-1 and Glypican-3 dysregulation is related to Notum and β-catenin alterations mostly in AOM/DSS-induced colorectal adenocarcinomas and in human colorectal tumors but also, in some cases, in early murine CRC stages.

These data suggest that Notum, Glypican-1 and Glypican-3 may be included in the variegated landscape of the different regulators of Wnt/β-catenin signaling. Furthermore, the promising results obtained in the set of human CRC samples encouraged us to suggest them as new biomarker candidates for CRC that should be validated in further clinical studies.

## MATERIALS AND METHODS

### Animals and sample processing

A total of 30 6-week-old Balb/c mice were intraperitoneally injected with a single dose of 10 mg/kg azoxymethane (AOM) (Sigma-Aldrich, St. Louis, MO) on day 1, followed by a single weekly cycle of 2% dextran sulfate sodium (DSS) (MP Biomedicals, Solon, OH; MW 36–50 kDa) administered in their drinking water. The mice were divided into four groups: 1) AOM/DSS treated, 2) AOM treated, 3) DSS treated, and 4) untreated (control). At the end of the 20th week, 6 mice per group were euthanised. Early colorectal lesions were investigated at an age of 5 weeks by immunohistochemistry in an extra group of 6 AOM/DSS-treated mice. All animal procedures were performed in accordance with institutional guidelines for laboratory animal care and in adherence with ethical standards. The study was approved by the Italian Ministry of Health according to the decree n. 336/2013-B.

The large intestine was removed from each mouse, cut open longitudinally along the main axis and flushed with cold PBS. The tumor masses from AOM/DSS treated mice, AOM-only and DSS-only treated colon mucosa, as well as normal mucosa, were cut, immediately placed in 5 volumes of RNAlater (Ambion, Austin, TX), and then stored at −80°C for RNA extraction. Other areas of the large intestine were fixed in 4% paraformaldehyde for 24 hours and embedded in paraffin. Histological sections (4 μm) of formalin-fixed paraffin-embedded (FFPE) samples were then prepared using routine procedures for histopathological analyses and immunohistochemistry.

### CRC patient samples

Tissues from 10 patients (5 males and 5 females; mean age of 74.1 ± 7.5 years) with CRC were obtained in collaboration with the Medical University of Plovdiv, Bulgaria. All patients signed informed consent in accordance with the WMA Declaration of Helsinki (2013). The study was approved by protocol # R-1838/15-07-2013. All patients were staged as T3N0 according to WHO classification (7^th^ Revision of the TNM system) with a G2 grade of cellular differentiation. On the basis of the TNM classification, none of them had detected metastasis at the time of diagnosis which gives us grounds to assume that these cases could be possibly comparable to the mouse tumors.

All tissue samples were analysed by two independent pathologists.

Tumor tissue and distal normal mucosa were isolated from all patients during surgical resection, fixed in formalin and embedded in paraffin. A portion of the tumor and normal mucosa was placed in RNAlater (Ambion, Austin, TX), immediately frozen after surgery and stored at −80°C until nucleic acid extraction.

### RNA extraction and purification

Total RNA was isolated from each tissue using TRIzol^®^ reagent (Invitrogen, Carlsbad, CA) according to the manufacturer's instructions. RNA was estimated qualitatively using an Agilent 2100 Bioanalyzer (Agilent, Santa Clara, CA) and quantified using a NanoVuePlus spectrophotometer (GE Healthcare, Milan, Italy). Any genomic DNA contamination was removed from the total RNA using a DNA-free kit (Ambion, Austin, TX, USA). Purified RNA was stored at −80°C until it was used.

### RNA microarray analysis

Murine cRNA was synthesized with an Illumina RNA amplification kit (Ambion), starting from 500 ng of total RNA and following the manufacturer's instructions. MouseWG-6 v2.0 Expression BeadChip hybridization, washing and staining were also carried out. Arrays were scanned on an Illumina BeadStation 500. BeadChip array data quality control was performed with the Illumina GenomeStudio software. The average probe intensity signal was calculated using GenomeStudio without background correction. Raw data were analysed with the oneChannelGUI [65] Bioconductor package [66]. Average probe intensities were log_2_ transformed and normalized by means of the Lowess method [67]. The number of genes to be evaluated was reduced by applying an interquartile (IQR) filter that removed non-significant probe sets (i.e., non-expressed and non-changing sets) [68]. Differentially expressed transcripts were detected by linear model analysis (BH corrected *p*-value ≤ 0.05 |log_2_FoldChange| ≥ 1). The following comparisons were considered: DSS-Control, AOM-Control, (AOM/DSS)-Control. Detected genes were loaded into IPA.

### Gene ontology analysis

The biological interpretation of the microarray data was conducted using Qiagen's Ingenuity Pathway Analysis version 7 (IPA7, http://www.ingenuity.com). Two parameters in the canonical pathway analysis, LogRatio and the *p*-value, measured the significance of the association between the data set and the canonical pathway. LogRatio represented the ratio of the number of genes from the dataset that mapped to the pathway divided by the total number of genes that mapped to the canonical pathway. The *p*-value was calculated with Fisher's exact test.

### Quantitative real time PCR (qPCR)

1 μg of murine DNase-treated RNA was retrotranscribed using RETROscript™ reagents (Ambion) and qPCRs were carried out using gene-specific primers (QuantiTect Primer Assay, Qiagen; Chatsworth, CA, USA; *GAPDH*:QT00079247, *B2M*:QT01149547, *DEFA6*:QT00 162491, *IL1RL1*:QT01063062, *WNT6*:QT01660883, *SOX4*:QT01755971, *GPR49*:QT00123193, *AQP8*:QT001 60559, *CAR4*:QT00095340, *SFRP1*:QT00167153, *NOTUM*:QT01749559, *GPC1*:QT00164017, *GPC3*: QT00118790, *AXIN2*:QT00126539), SYBR green and 7900HT RT-PCR Systems (Applied Biosystems, Milan, Italy). 1 μg of human DNase-treated RNA was reverse-transcribed using High Capacity cDNA Reverse Transcription kit (Applied Biosystems) and qPCRs were set up using Taqman^®^ Gene Expression Assays (Applied Biosystems; Assay ID: NOTUM:Hs00394510_m1; GPC1:Hs00892476_m1; GPC3:Hs01018936_m1) and Taqman^®^ Gene Expression Master Mix (Applied Biosystems). Reactions were run on 7900HT Fast RT-PCR System (Applied Biosystems). Data were analyzed using SDS software 2.3 (Applied Biosystems). Relative gene expression was quantified using the threshold cycle (C_T_) value and normalized to housekeeping genes β2-microglobulin and GAPDH (for murine RNA analysis) and β-actin and RNA18S5 (for human RNA analysis).

The results of the murine sample analysis were expressed as Delta C_T_, which represents the difference in C_T_ values between the gene of interest and the β2-microglobulin reference gene. GAPDH normalized data gave comparable results (data not shown). Relative expression in the human tumor samples compared with distal normal colon tissue (calibrator) was calculated according to the method of Fold Change (2^-(DeltaDelta C_T_)). β-actin and RNA18S5 normalized data gave comparable results.

Statistical calculations were performed with Prism 3.0 software (GraphPad Software). Student's unpaired *t*-test was used to analyse the qPCR results. The data were considered to be significant at *p* < 0.001.

### Histopathological analysis and immunohistochemistry

Both murine and human FFPE sections were stained using hematoxylin (H) and eosin (E) according to standard histochemical procedures. Mouse colonic mucosa lesions (Aberrant Crypt Foci - ACF, microadenoma, adenoma, adenocarcinoma) were diagnosed according to the histopathological criteria described by Boivin et al [69]. Immunohistochemistry was performed on 4-μm-thick FFPE tissue sections after antigen retrieval in sodium citrate buffer (10 mM sodium citrate, pH 6), for 40 minutes at 95°C. The samples were incubated with rabbit anti-human/mouse Notum (ThermoFisher Scientific Inc, IL, USA, 1:300); goat anti- human/mouse Glypican 1 (Santa Cruz Biotechnology, Santa Cruz, CA, USA, 1:50), rat anti-mouse Glypican 3 (Novus Biologicals, LLC, Littleton, CO, USA, 1:50); rat anti-human Glypican 3 (ThermoFisher Scientific Inc, IL, USA, 1:100) or rabbit anti-mouse β-catenin (Santa Cruz Biotechnology, Santa Cruz, CA, 1:100) antibodies overnight at 4°C, followed by biotin-labelled secondary antibody and HRP-conjugated avidin for 30 minutes at room temperature. Detection was achieved using a substrate/chromogen mixture (DAB) and hematoxylin counterstaining. Incubation with the primary antibody was omitted for the negative controls. The immunostained slides were observed under a microscope, and the image data were analysed using NIS FreeWare 2.10 software (Nikon, Japan). A semi-quantitative intensity scale ranging from (−) for 10–20% immunopositive cells (low staining) to (+) for 40–60% immunopositive cells (medium staining) and (++) 80–100% immunopositive cells for intense staining was adopted.

## SUPPLEMENTARY TABLE




