# Supplementary material for: Novel insights into Notum and glypicans regulation in colorectal cancer
Source: Oncotarget. 2015 Oct 20;6(38):41237–57. doi: 10.18632/oncotarget.5652 (PMC4747403; doi:10.18632/oncotarget.5652)
Supplement: Supplementary file 1 [file oncotarget-06-41237-s001.pdf]

SUPPLEMENTARY TABLE

Supplementary Table S2: Genes involved in the top 2 significantly dysregulated pathways

Canonical pathway	Entrez Gene ID	Symbol	Entrez Gene Name	Log Ratio	*p*-value
LPS/IL-1-mediated inhibition of RXR function signaling	17082	IL1RL1	interleukin 1 receptor-like 1	4.748	1.07E-21
	18383	TNFRSF11B	tumor necrosis factor receptor superfamily, 11b	3.684	1.51E-16
	11425	APOC4	apolipoprotein C-IV	2.907	1.27E-05
	239273	ABCC4	ATP-binding cassette, sub-family C (CFTR/MRP), 4	2.716	2.63E-15
	228608	SMOX	spermine oxidase	2.091	2.51E-13
	16176	IL1B	interleukin 1 beta	1.779	1.23E-06
	214579	ALDH5A1	aldehyde dehydrogenase 5 family, member A1	1.580	8.03E-13
	14859	GSTA3	glutathione S-transferase alpha 3	1.438	1.68E-04
	68312	GSTM2	glutathione S-transferase mu 2 (muscle)	1.386	9.99E-11
	13088	CYP2B6	cytochrome P450, family 2, subfamily B, polypep. 6	1.369	1.40E-09
	12780	ABCC2	ATP-binding cassette, sub-family C (CFTR/MRP), 2	1.240	4.39E-12
	56454	ALDH18A1	aldehyde dehydrogenase 18 family, member A1	1.161	1.14E-09
	11816	APOE	apolipoprotein E	1.120	5.01E-03
	15478	HS3ST3A1	heparan sulfate 3-O-sulfotransferase 3A1	1.090	3.85E-06
	16476	JUN	jun proto-oncogene	1.053	9.34E-07
	21926	TNF	tumor necrosis factor	1.025	9.24E-08
	50790	ACSL4	acyl-CoA synthetase long-chain family member 4	1.019	1.65E-11
	72082	CYP2C9	cytochrome P450, family 2, subfamily C, polypep. 9	−3.902	6.66E-12
	53315	Sult1d1	sulfotransferase family 1D, member 1	−3.123	1.28E-14
	27409	ABCG5	ATP-binding cassette, sub-family G (WHITE), 5	−2.625	5.88E-10
	109731	MAOB	monoamine oxidase B	−2.527	3.72E-11
	69083	SULT1C2	sulfotransferase family, cytosolic, 1C, member 2	−2.358	5.79E-10

(*Continued*)

Canonical pathway	Entrez Gene ID	Symbol	Entrez Gene Name	Log Ratio	*p*-value
	16173	IL18	interleukin 18 (interferon-gamma-inducing factor)	−2.246	1.86E-10
	20186	NR1H4	nuclear receptor subfamily 1, group H, member 4	−2.229	8.93E-11
	19017	PPARGC1A	peroxisome proliferator-activated receptor gamma,1α	−2.160	4.28E-09
	381334	GAL3ST2	galactose-3-O-sulfotransferase 2	−2.145	3.47E-13
	170826	PPARGC1B	peroxisome proliferator-activated receptor gamma,1β	−2.008	2.21E-11
	67470	ABCG8	ATP-binding cassette, sub-family G (WHITE), 8	−1.835	1.25E-06
	26424	NR5A2	nuclear receptor subfamily 5, group A, member 2	−1.673	6.36E-11
	66447	MGST3	microsomal glutathione S-transferase 3	−1.460	5.90E-08
	14871	GSTT1	glutathione S-transferase theta 1	−1.430	1.35E-11
	56615	MGST1	microsomal glutathione S-transferase 1	−1.420	1.88E-08
	76408	ABCC3	ATP-binding cassette, sub-family C (CFTR/MRP), 3	−1.416	1.99E-10
	18171	NR1I2	nuclear receptor subfamily 1, group I, member 2	−1.382	2.59E-10
	74205	ACSL3	acyl-CoA synthetase long-chain family member 3	−1.279	2.81E-07
	11812	APOC1	apolipoprotein C-I	−1.193	4.35E-02
	54200	SULT2B1	sulfotransferase family, cytosolic, 2B, member 1	−1.189	2.23E-09
	104776	ALDH6A1	aldehyde dehydrogenase 6 family, member A1	−1.062	1.09E-06
	12359	CAT	catalase	−1.031	3.16E-07
Wnt/β-catenin signaling	12006	AXIN2	axin 2	4.159	1.37E-15
	20677	SOX4	SRY (sex determining region Y)-box 4	3.478	1.75E-14
	12443	CCND1	cyclin D1	3.134	1.45E-15
	22420	WNT6	wingless-type MMTV integration site family, 6	3.058	3.77E-17
	20671	SOX17	SRY (sex determining region Y)-box 17	2.794	1.39E-14
	24117	WIF1	WNT inhibitory factor 1	2.719	2.99E-15
	12505	CD44	CD44 molecule (Indian blood group)	2.661	2.70E-15
	93897	FZD10	frizzled family receptor 10	2.604	1.63E-11

(*Continued*)

Canonical pathway	Entrez Gene ID	Symbol	Entrez Gene Name	Log Ratio	*p*-value
	22409	WNT10A	wingless-type MMTV integration site family, 10A	2.538	1.34E-15
	50781	DKK3	dickkopf 3 homolog (Xenopus laevis)	2.325	1.99E-11
	21413	TCF4	transcription factor 4	2.259	2.38E-15
	21885	TLE1	transducin-like enhancer of split 1	1.842	6.60E-14
	218772	RARB	retinoic acid receptor, beta	1.737	1.12E-11
	104318	CSNK1D	casein kinase 1, delta	1.637	3.20E-08
	14609	GJA1	gap junction protein, alpha 1, 43kDa	1.591	4.52E-09
	20682	SOX9	SRY (Sex Determining Region Y)-Box 9	1.572	2.16E-07
	27373	CSNK1E	casein kinase 1, epsilon	1.542	2.36E-09
	20672	SOX18	SRY (sex determining region Y)-box 18	1.228	8.97E-07
	22059	TP53	tumor protein p53	1.185	3.56E-07
	18099	NLK	nemo-like kinase	1.157	1.06E-08
	20667	SOX12	SRY (sex determining region Y)-box 12	1.118	4.51E-09
	22418	WNT5A	wingless-type MMTV integration site family, 5A	1.098	1.82E-06
	16476	JUN	jun proto-oncogene	1.053	9.34E-07
	20377	SFRP1	secreted frizzled-related protein 1	−1.997	1.11E-07
	24108	UBD	ubiquitin D	−1.727	1.08E-09
	26424	NR5A2	nuclear receptor subfamily 5, group A, member 2	−1.673	6.36E-11
	14367	FZD5	frizzled family receptor 5	−1.529	2.83E-10
	21416	TCF7L2	transcription factor 7-like 2 (T-cell specific, HMG-box)	−1.060	1.38E-09
	21814	TGFBR3	transforming growth factor, beta receptor III	−1.048	8.64E-06
	226849	PPP2R5A	protein phosphatase 2, regulatory subunit B’, alpha	−1.011	5.03E-10
