# Supplementary material for: Novel insights into Notum and glypicans regulation in colorectal cancer
Source: Oncotarget. 2015 Oct 20;6(38):41237–57. doi: 10.18632/oncotarget.5652 (PMC4747403; doi:10.18632/oncotarget.5652)
Supplement: Supplementary file 2 [file oncotarget-06-41237-s002.doc]

**Supplementary Table 1: important targets and members of the Wnt signalling pathway differentially expressed in adenocarcinoma.**

| **IlluID** | **Entrez** | **SYMBOL** | **log2FC** | **log2 average** | **BH corrected** | **Experimental Group** |
| --- | --- | --- | --- | --- | --- | --- |
|  | **GeneID** |  |  | **expression** | **p-value** |  |
| ILMN_2718910 | 230810 | Slc30a2 | 4.687 | 9.020 | 3.28E-22 | adenocarcinoma |
| ILMN_2727163 | 18780 | Pla2g2a | 6.681 | 10.187 | 3.28E-22 | adenocarcinoma |
| ILMN_1225909 | 18946 | Pnliprp1 | 6.400 | 9.935 | 3.28E-22 | adenocarcinoma |
| ILMN_1254739 | 13240 | Defa6 | 6.784 | 9.436 | 5.95E-22 | adenocarcinoma |
| ILMN_2757617 | 17082 | Il1rl1 | 4.748 | 9.351 | 1.07E-21 | adenocarcinoma |
| ILMN_1212867 | 100503970 | AY761185 | 6.614 | 9.370 | 2.36E-20 | adenocarcinoma |
| ILMN_2524832 | NA | NA | 6.122 | 9.373 | 2.68E-20 | adenocarcinoma |
| ILMN_1230926 | 23966 | Tenm4 | 4.669 | 9.301 | 4.55E-20 | adenocarcinoma |
| ILMN_2504268 | 94214 | Spock2 | 5.115 | 9.332 | 4.86E-20 | adenocarcinoma |
| ILMN_1230099 | NA | NA | 6.291 | 9.434 | 8.28E-20 | adenocarcinoma |
| ILMN_3160369 | 213948 | Atg9b | 4.667 | 9.138 | 8.28E-20 | adenocarcinoma |
| ILMN_1251096 | 94214 | Spock2 | 3.668 | 8.918 | 1.24E-19 | adenocarcinoma |
| ILMN_1235329 | 104156 | Etv5 | 3.198 | 8.930 | 1.24E-19 | adenocarcinoma |
| ILMN_2739019 | NA | NA | 5.857 | 9.248 | 1.93E-19 | adenocarcinoma |
| ILMN_3162820 | 23966 | Tenm4 | 4.021 | 9.161 | 3.19E-19 | adenocarcinoma |
| ILMN_2445249 | 16673 | Krt36 | 5.249 | 9.969 | 4.66E-19 | adenocarcinoma |
| ILMN_1214498 | 56448 | Cyp2d22 | -3.262 | 11.148 | 1.09E-18 | adenocarcinoma |
| ILMN_2669793 | 12443 | Ccnd1 | 2.463 | 9.481 | 2.60E-18 | adenocarcinoma |
| ILMN_2607813 | 13222 | Defa-rs2 | 6.039 | 9.413 | 2.60E-18 | adenocarcinoma |
| ILMN_2655795 | 12351 | Car4 | -4.695 | 12.544 | 2.68E-18 | adenocarcinoma |
| ILMN_2772632 | 20210 | Saa3 | 4.339 | 9.169 | 3.51E-18 | adenocarcinoma |
| ILMN_2457437 | NA | NA | 3.891 | 9.515 | 3.98E-18 | adenocarcinoma |
| ILMN_2741063 | 108897 | Aif1l | 2.920 | 8.695 | 4.62E-18 | adenocarcinoma |
| ILMN_2524945 | NA | NA | 5.951 | 9.290 | 6.54E-18 | adenocarcinoma |
| ILMN_2423835 | 22420 | Wnt6 | 3.025 | 9.040 | 1.40E-17 | adenocarcinoma |
| ILMN_1222084 | 140743 | Rem2 | 2.872 | 8.769 | 1.83E-17 | adenocarcinoma |
| ILMN_2990661 | 18947 | Pnliprp2 | 4.506 | 9.186 | 1.89E-17 | adenocarcinoma |
| ILMN_2993174 | 13218 | Defa-rs1 | 4.715 | 9.124 | 1.92E-17 | adenocarcinoma |
| ILMN_2696611 | 93960 | Nkd1 | 3.719 | 9.213 | 2.32E-17 | adenocarcinoma |
| ILMN_2658501 | 66141 | Ifitm3 | 3.876 | 12.141 | 3.00E-17 | adenocarcinoma |
| ILMN_2901765 | 22420 | Wnt6 | 3.058 | 8.974 | 3.77E-17 | adenocarcinoma |
| ILMN_2746738 | 56213 | Htra1 | 3.099 | 10.068 | 4.17E-17 | adenocarcinoma |
| ILMN_2700608 | 20907 | Stx1a | 3.222 | 9.201 | 4.48E-17 | adenocarcinoma |
| ILMN_2671165 | 94179 | Krt23 | 4.780 | 10.486 | 4.48E-17 | adenocarcinoma |
| ILMN_2775937 | 18791 | Plat | 4.649 | 9.861 | 4.51E-17 | adenocarcinoma |
| ILMN_2710914 | 13238 | Defa4 | 5.503 | 9.320 | 4.51E-17 | adenocarcinoma |
| ILMN_1217180 | 68713 | Ifitm1 | 3.899 | 12.010 | 4.51E-17 | adenocarcinoma |
| ILMN_2607127 | 11514 | Adcy8 | 2.366 | 8.569 | 4.76E-17 | adenocarcinoma |
| ILMN_1231270 | 13222 | Defa-rs2 | 5.020 | 9.083 | 4.99E-17 | adenocarcinoma |
| ILMN_2524846 | 13238 | Defa4 | 5.716 | 9.260 | 5.20E-17 | adenocarcinoma |
| ILMN_1231513 | 56753 | Tacstd2 | 4.353 | 9.148 | 5.20E-17 | adenocarcinoma |
| ILMN_1258881 | 56643 | Slc15a1 | -3.024 | 10.379 | 5.60E-17 | adenocarcinoma |
| ILMN_2613908 | 11684 | Alox12 | 3.599 | 8.904 | 5.60E-17 | adenocarcinoma |
| ILMN_2731113 | 14028 | Evx1 | 3.041 | 9.142 | 5.60E-17 | adenocarcinoma |
| ILMN_2619952 | 17384 | Mmp10 | 5.223 | 9.592 | 5.87E-17 | adenocarcinoma |
| ILMN_1377919 | 73710 | Tubb2b | 3.676 | 10.522 | 6.75E-17 | adenocarcinoma |
| ILMN_2875737 | 380997 | Cyp2d12 | -3.701 | 10.822 | 7.34E-17 | adenocarcinoma |
| ILMN_2932164 | 77583 | Notum | 5.003 | 9.207 | 1.04E-16 | adenocarcinoma |
| ILMN_2710905 | 20201 | S100a8 | 3.353 | 8.786 | 1.05E-16 | adenocarcinoma |
| ILMN_2737685 | 17386 | Mmp13 | 5.017 | 10.194 | 1.10E-16 | adenocarcinoma |
| ILMN_2753149 | 11567 | Avil | 3.226 | 8.898 | 1.21E-16 | adenocarcinoma |
| ILMN_1244310 | 226866 | Sbspon | 3.240 | 8.657 | 1.33E-16 | adenocarcinoma |
| ILMN_2513826 | 18383 | Tnfrsf11b | 3.684 | 9.564 | 1.51E-16 | adenocarcinoma |
| ILMN_2888552 | 55963 | Slc1a4 | 2.986 | 8.996 | 1.58E-16 | adenocarcinoma |
| ILMN_2598766 | 19130 | Prox1 | 4.053 | 9.224 | 2.13E-16 | adenocarcinoma |
| ILMN_1246808 | 20720 | Serpine2 | 3.296 | 9.590 | 2.24E-16 | adenocarcinoma |
| ILMN_2534431 | 16673 | Krt36 | 4.251 | 9.180 | 2.29E-16 | adenocarcinoma |
| ILMN_2742426 | 20341 | Selenbp1 | -4.122 | 13.608 | 2.56E-16 | adenocarcinoma |
| ILMN_2598877 | 50934 | Slc7a8 | 2.584 | 8.723 | 2.56E-16 | adenocarcinoma |
| ILMN_2692167 | 18947 | Pnliprp2 | 3.908 | 9.236 | 3.06E-16 | adenocarcinoma |
| ILMN_1244484 | 19876 | Robo1 | 3.192 | 9.258 | 3.16E-16 | adenocarcinoma |
| ILMN_2685770 | 93840 | Vangl2 | 2.050 | 8.602 | 3.16E-16 | adenocarcinoma |
| ILMN_1214071 | 68713 | Ifitm1 | 4.478 | 11.113 | 3.43E-16 | adenocarcinoma |
| ILMN_2641201 | 64406 | Sp5 | 3.398 | 8.739 | 3.64E-16 | adenocarcinoma |
| ILMN_2938047 | 104156 | Etv5 | 2.311 | 8.669 | 3.64E-16 | adenocarcinoma |
| ILMN_3139253 | 74007 | Btbd11 | 2.719 | 8.893 | 4.50E-16 | adenocarcinoma |
| ILMN_1247199 | 26914 | H2afy | -2.340 | 8.712 | 5.57E-16 | adenocarcinoma |
| ILMN_2756023 | 100102 | Pcsk9 | 3.744 | 9.796 | 5.57E-16 | adenocarcinoma |
| ILMN_2985042 | 16673 | Krt36 | 4.360 | 9.019 | 6.07E-16 | adenocarcinoma |
| ILMN_2705119 | 12226 | Btg1 | -2.391 | 13.624 | 7.34E-16 | adenocarcinoma |
| ILMN_3156058 | 68938 | Aspscr1 | 3.094 | 10.187 | 7.61E-16 | adenocarcinoma |
| ILMN_2611180 | 74186 | Ccdc3 | 2.840 | 9.565 | 8.10E-16 | adenocarcinoma |
| ILMN_2966104 | 56213 | Htra1 | 3.345 | 9.820 | 8.25E-16 | adenocarcinoma |
| ILMN_2705390 | 171168 | Acer1 | -3.017 | 11.497 | 8.25E-16 | adenocarcinoma |
| ILMN_1221503 | 12443 | Ccnd1 | 3.087 | 11.248 | 8.50E-16 | adenocarcinoma |
| ILMN_1215096 | 12499 | Entpd5 | -1.823 | 10.293 | 8.83E-16 | adenocarcinoma |
| ILMN_2728682 | 320111 | Prr18 | 2.931 | 9.276 | 9.17E-16 | adenocarcinoma |
| ILMN_2727309 | NA | NA | -4.231 | 13.479 | 9.18E-16 | adenocarcinoma |
| ILMN_2740391 | 17752 | Mt4 | 3.328 | 8.790 | 1.02E-15 | adenocarcinoma |
| ILMN_2635784 | 14733 | Gpc1 | 4.314 | 11.009 | 1.03E-15 | adenocarcinoma |
| ILMN_2669974 | 13216 | Defa1 | 4.077 | 8.915 | 1.13E-15 | adenocarcinoma |
| ILMN_2764651 | 17751 | Mt3 | 3.878 | 8.799 | 1.14E-15 | adenocarcinoma |
| ILMN_2460094 | 22409 | Wnt10a | 2.538 | 8.557 | 1.34E-15 | adenocarcinoma |
| ILMN_2896314 | 12006 | Axin2 | 4.159 | 10.223 | 1.37E-15 | adenocarcinoma |
| ILMN_1216764 | 15937 | Ier3 | 3.714 | 10.633 | 1.45E-15 | adenocarcinoma |
| ILMN_2601471 | 12443 | Ccnd1 | 3.134 | 10.928 | 1.45E-15 | adenocarcinoma |
| ILMN_1246733 | 195209 | Gm22 | 2.866 | 8.767 | 1.45E-15 | adenocarcinoma |
| ILMN_2621433 | 116701 | Fgfrl1 | 2.902 | 9.428 | 1.61E-15 | adenocarcinoma |
| ILMN_2608858 | 23919 | Insl5 | -3.684 | 11.115 | 1.77E-15 | adenocarcinoma |
| ILMN_2745370 | 20887 | Sult1a1 | -3.195 | 12.295 | 1.78E-15 | adenocarcinoma |
| ILMN_1230224 | 72685 | Dnajc6 | 2.529 | 9.132 | 1.94E-15 | adenocarcinoma |
| ILMN_2620435 | 51797 | Ctps | 2.007 | 10.634 | 2.35E-15 | adenocarcinoma |
| ILMN_2636463 | 21413 | Tcf4 | 2.259 | 11.992 | 2.38E-15 | adenocarcinoma |
| ILMN_2731331 | 329064 | Pkd2l1 | 3.986 | 8.822 | 2.47E-15 | adenocarcinoma |
| ILMN_1232667 | 80876 | Ifitm2 | 2.624 | 12.348 | 2.63E-15 | adenocarcinoma |
| ILMN_2621708 | 239273 | Abcc4 | 2.716 | 9.248 | 2.63E-15 | adenocarcinoma |
| ILMN_2592953 | 20394 | Scg5 | -1.890 | 9.313 | 2.67E-15 | adenocarcinoma |
| ILMN_1223697 | 12505 | Cd44 | 2.661 | 9.674 | 2.70E-15 | adenocarcinoma |
| ILMN_1239726 | 30927 | Snai3 | 1.819 | 8.361 | 2.76E-15 | adenocarcinoma |
| ILMN_2961152 | 110310 | Krt7 | 2.370 | 10.821 | 2.90E-15 | adenocarcinoma |
| ILMN_2857748 | 24117 | Wif1 | 2.719 | 8.791 | 2.99E-15 | adenocarcinoma |
| ILMN_1235131 | 228366 | Gyltl1b | 2.185 | 9.902 | 3.67E-15 | adenocarcinoma |
| ILMN_1259610 | 102657 | Cd276 | 2.081 | 8.507 | 4.18E-15 | adenocarcinoma |
| ILMN_1213286 | NA | NA | -3.135 | 10.598 | 4.41E-15 | adenocarcinoma |
| ILMN_2635631 | 20350 | Sema3f | 3.471 | 9.866 | 4.53E-15 | adenocarcinoma |
| ILMN_2534783 | 234669 | Ces2b | -3.243 | 11.001 | 6.07E-15 | adenocarcinoma |
| ILMN_3103896 | 21857 | Timp1 | 2.180 | 9.097 | 6.07E-15 | adenocarcinoma |
| ILMN_2854157 | 11833 | Aqp8 | -4.896 | 13.205 | 6.32E-15 | adenocarcinoma |
| ILMN_2592321 | 12554 | Cdh13 | 2.447 | 9.011 | 6.45E-15 | adenocarcinoma |
| ILMN_2618935 | 67182 | Pdzk1ip1 | -2.802 | 11.168 | 7.40E-15 | adenocarcinoma |
| ILMN_2845849 | 19775 | Xpr1 | -2.012 | 11.277 | 7.40E-15 | adenocarcinoma |
| ILMN_3149143 | 12499 | Entpd5 | -1.894 | 10.731 | 7.89E-15 | adenocarcinoma |
| ILMN_2895312 | 108112 | Eif4ebp3 | -2.851 | 10.607 | 8.51E-15 | adenocarcinoma |
| ILMN_2700627 | 13113 | Cyp3a13 | -2.776 | 10.561 | 8.54E-15 | adenocarcinoma |
| ILMN_2447991 | NA | NA | 2.544 | 8.598 | 8.54E-15 | adenocarcinoma |
| ILMN_2760064 | 230393 | Focad | 2.410 | 10.938 | 8.61E-15 | adenocarcinoma |
| ILMN_1254295 | 223227 | Sox21 | 3.402 | 10.472 | 9.12E-15 | adenocarcinoma |
| ILMN_2640248 | 16776 | Lama5 | 2.519 | 8.844 | 9.33E-15 | adenocarcinoma |
| ILMN_2756379 | 11833 | Aqp8 | -4.961 | 13.313 | 1.05E-14 | adenocarcinoma |
| ILMN_2439341 | 22321 | Vars | 2.124 | 9.424 | 1.16E-14 | adenocarcinoma |
| ILMN_2640848 | NA | NA | -2.667 | 9.966 | 1.18E-14 | adenocarcinoma |
| ILMN_2890534 | 13239 | Defa5 | 3.291 | 8.630 | 1.20E-14 | adenocarcinoma |
| ILMN_2754985 | 21664 | Phlda1 | 3.617 | 9.916 | 1.26E-14 | adenocarcinoma |
| ILMN_2641217 | 53315 | Sult1d1 | -3.123 | 11.123 | 1.28E-14 | adenocarcinoma |
| ILMN_1220115 | 19205 | Ptbp1 | 1.708 | 10.409 | 1.39E-14 | adenocarcinoma |
| ILMN_2647873 | 215690 | Nav1 | 1.956 | 9.997 | 1.39E-14 | adenocarcinoma |
| ILMN_2747986 | 20671 | Sox17 | 2.794 | 8.523 | 1.39E-14 | adenocarcinoma |
| ILMN_2639214 | 13113 | Cyp3a13 | -3.127 | 11.303 | 1.46E-14 | adenocarcinoma |
| ILMN_2518483 | 66350 | Pla2g12a | 1.698 | 8.564 | 1.54E-14 | adenocarcinoma |
| ILMN_2939652 | 20342 | Selenbp2 | -3.427 | 11.250 | 1.59E-14 | adenocarcinoma |
| ILMN_2745614 | 66270 | Fam134b | -2.486 | 11.914 | 1.60E-14 | adenocarcinoma |
| ILMN_2883016 | 215690 | Nav1 | 2.065 | 9.797 | 1.60E-14 | adenocarcinoma |
| ILMN_2727116 | 20677 | Sox4 | 3.478 | 10.176 | 1.75E-14 | adenocarcinoma |
| ILMN_1239717 | 14038 | Wfdc18 | 4.156 | 9.482 | 1.82E-14 | adenocarcinoma |
| ILMN_1257444 | 56857 | Slc37a2 | -4.432 | 10.937 | 1.82E-14 | adenocarcinoma |
| ILMN_2653207 | 21677 | Tead2 | 2.737 | 8.990 | 1.85E-14 | adenocarcinoma |
| ILMN_2648317 | 72273 | 2210404O07Rik | -3.656 | 12.495 | 1.92E-14 | adenocarcinoma |
| ILMN_2750588 | 98170 | Tmem132a | 1.600 | 8.580 | 2.08E-14 | adenocarcinoma |
| ILMN_2769918 | 21857 | Timp1 | 2.188 | 9.137 | 2.35E-14 | adenocarcinoma |
| ILMN_2864062 | 22337 | Vdr | -1.823 | 9.427 | 2.35E-14 | adenocarcinoma |
| ILMN_1244340 | 20997 | T | 1.835 | 8.416 | 2.41E-14 | adenocarcinoma |
| ILMN_2712895 | 109292 | 4631423B10Rik | 1.801 | 8.829 | 2.49E-14 | adenocarcinoma |
| ILMN_2855334 | 110006 | Gusb | 2.026 | 11.188 | 2.57E-14 | adenocarcinoma |
| ILMN_3139514 | 21386 | Tbx3 | 2.779 | 8.738 | 2.58E-14 | adenocarcinoma |
| ILMN_2894355 | 19130 | Prox1 | 2.369 | 8.488 | 2.64E-14 | adenocarcinoma |
| ILMN_1248740 | 20350 | Sema3f | 3.495 | 10.424 | 2.73E-14 | adenocarcinoma |
| ILMN_1223261 | NA | NA | -1.624 | 9.947 | 2.90E-14 | adenocarcinoma |
| ILMN_3001481 | 11443 | Chrnb1 | 2.266 | 10.444 | 2.94E-14 | adenocarcinoma |
| ILMN_1214765 | 19775 | Xpr1 | -1.839 | 11.632 | 2.94E-14 | adenocarcinoma |
| ILMN_2752994 | 67731 | Fbxo32 | -2.227 | 10.372 | 2.94E-14 | adenocarcinoma |
| ILMN_2461871 | 107767 | Scamp1 | -1.757 | 10.436 | 3.27E-14 | adenocarcinoma |
| ILMN_2606162 | 30794 | Pdlim4 | 2.322 | 10.917 | 3.27E-14 | adenocarcinoma |
| ILMN_3137899 | 21906 | Otop1 | 2.097 | 8.550 | 3.31E-14 | adenocarcinoma |
| ILMN_1231872 | 13222 | Defa-rs2 | 4.002 | 8.792 | 3.52E-14 | adenocarcinoma |
| ILMN_2721357 | 218454 | Lhfpl2 | -2.914 | 12.053 | 3.52E-14 | adenocarcinoma |
| ILMN_1220508 | 73713 | Rbm20 | 2.119 | 8.970 | 3.79E-14 | adenocarcinoma |
| ILMN_3141048 | 20363 | Sepp1 | -3.344 | 12.833 | 3.79E-14 | adenocarcinoma |
| ILMN_2602139 | NA | NA | -2.624 | 10.017 | 3.79E-14 | adenocarcinoma |
| ILMN_2776278 | 17069 | Ly6e | 3.453 | 9.994 | 3.98E-14 | adenocarcinoma |
| ILMN_2925947 | 268860 | Abat | -2.707 | 10.110 | 4.04E-14 | adenocarcinoma |
| ILMN_2699522 | 226419 | Dyrk3 | 2.367 | 8.506 | 4.14E-14 | adenocarcinoma |
| ILMN_2948143 | 26570 | Slc7a11 | 1.435 | 8.375 | 4.50E-14 | adenocarcinoma |
| ILMN_2890357 | 230752 | Eva1b | 1.707 | 9.072 | 4.55E-14 | adenocarcinoma |
| ILMN_2445166 | 22337 | Vdr | -2.789 | 11.509 | 4.71E-14 | adenocarcinoma |
| ILMN_1258189 | NA | NA | -2.468 | 10.155 | 4.76E-14 | adenocarcinoma |
| ILMN_2711948 | 20539 | Slc7a5 | 2.544 | 9.240 | 5.24E-14 | adenocarcinoma |
| ILMN_1229535 | 13101 | Cyp2d10 | -3.101 | 11.637 | 5.38E-14 | adenocarcinoma |
| ILMN_1217097 | 63955 | Cables1 | -2.316 | 11.544 | 6.05E-14 | adenocarcinoma |
| ILMN_1213978 | 19277 | Ptpro | 2.286 | 8.895 | 6.06E-14 | adenocarcinoma |
| ILMN_2961221 | 24059 | Slco2a1 | -1.863 | 9.994 | 6.43E-14 | adenocarcinoma |
| ILMN_1230788 | 21885 | Tle1 | 1.842 | 10.008 | 6.60E-14 | adenocarcinoma |
| ILMN_2588051 | NA | NA | 2.413 | 9.869 | 6.60E-14 | adenocarcinoma |
| ILMN_2634667 | 17288 | Mep1b | -2.309 | 9.999 | 6.88E-14 | adenocarcinoma |
| ILMN_1232176 | 100515 | Zfp518b | 1.818 | 9.565 | 7.04E-14 | adenocarcinoma |
| ILMN_2600565 | 80884 | Maged2 | 2.013 | 10.961 | 7.48E-14 | adenocarcinoma |
| ILMN_2712075 | 16819 | Lcn2 | 3.919 | 9.555 | 7.89E-14 | adenocarcinoma |
| ILMN_2682865 | 68938 | Aspscr1 | 2.601 | 9.641 | 8.05E-14 | adenocarcinoma |
| ILMN_1252655 | 58210 | Sectm1b | -2.520 | 13.651 | 8.35E-14 | adenocarcinoma |
| ILMN_1221048 | 107272 | Psat1 | 1.729 | 8.711 | 9.34E-14 | adenocarcinoma |
| ILMN_2990220 | 216825 | Usp22 | 1.691 | 11.054 | 9.34E-14 | adenocarcinoma |
| ILMN_1256622 | 381680 | Nxpe5 | -2.225 | 9.938 | 9.34E-14 | adenocarcinoma |
| ILMN_2772070 | 13370 | Dio1 | -2.312 | 9.856 | 9.51E-14 | adenocarcinoma |
| ILMN_2691352 | 13639 | Efna4 | 1.415 | 8.660 | 1.07E-13 | adenocarcinoma |
| ILMN_1240726 | 17387 | Mmp14 | 2.821 | 11.760 | 1.10E-13 | adenocarcinoma |
| ILMN_2982781 | 16529 | Kcnk5 | -2.536 | 11.974 | 1.11E-13 | adenocarcinoma |
| ILMN_2545805 | NA | NA | -3.534 | 11.043 | 1.13E-13 | adenocarcinoma |
| ILMN_1233840 | 16560 | Kif1a | 2.382 | 10.092 | 1.17E-13 | adenocarcinoma |
| ILMN_2691897 | NA | NA | 2.956 | 8.523 | 1.19E-13 | adenocarcinoma |
| ILMN_2692527 | NA | NA | 2.819 | 8.454 | 1.40E-13 | adenocarcinoma |
| ILMN_2522884 | NA | NA | 1.901 | 8.720 | 1.44E-13 | adenocarcinoma |
| ILMN_1230263 | 107029 | Me2 | -1.659 | 10.457 | 1.48E-13 | adenocarcinoma |
| ILMN_1241332 | 320772 | Mdga2 | 1.882 | 8.500 | 1.61E-13 | adenocarcinoma |
| ILMN_2433848 | NA | NA | -2.175 | 10.388 | 1.67E-13 | adenocarcinoma |
| ILMN_2649199 | 15446 | Hpgd | -2.573 | 10.932 | 1.69E-13 | adenocarcinoma |
| ILMN_3160326 | 19092 | Prkg2 | 1.997 | 8.589 | 1.73E-13 | adenocarcinoma |
| ILMN_2612774 | 231801 | Agfg2 | -1.501 | 10.917 | 1.77E-13 | adenocarcinoma |
| ILMN_1245549 | 80982 | 9930013L23Rik | 3.241 | 8.638 | 1.78E-13 | adenocarcinoma |
| ILMN_2718638 | 56185 | Hao2 | -2.434 | 10.188 | 1.78E-13 | adenocarcinoma |
| ILMN_2685921 | 11443 | Chrnb1 | 1.641 | 9.251 | 1.78E-13 | adenocarcinoma |
| ILMN_2445165 | 22337 | Vdr | -2.568 | 10.953 | 1.82E-13 | adenocarcinoma |
| ILMN_2625940 | 18011 | Neurl1a | 2.375 | 9.867 | 1.96E-13 | adenocarcinoma |
| ILMN_1253045 | 99709 | AI747448 | 4.353 | 9.651 | 1.96E-13 | adenocarcinoma |
| ILMN_2725914 | 15013 | H2-Q2 | -2.699 | 11.024 | 2.02E-13 | adenocarcinoma |
| ILMN_2424299 | 27279 | Tnfrsf12a | 2.446 | 9.319 | 2.13E-13 | adenocarcinoma |
| ILMN_1225257 | 74556 | Themis3 | -2.578 | 11.829 | 2.17E-13 | adenocarcinoma |
| ILMN_2965660 | 494504 | Apcdd1 | 4.014 | 9.027 | 2.17E-13 | adenocarcinoma |
| ILMN_1250569 | 268480 | Rapgefl1 | -1.813 | 10.623 | 2.25E-13 | adenocarcinoma |
| ILMN_1240972 | 68108 | 9430008C03Rik | 1.754 | 10.045 | 2.25E-13 | adenocarcinoma |
| ILMN_1244272 | 13823 | Epb4.1l3 | -2.074 | 12.260 | 2.25E-13 | adenocarcinoma |
| ILMN_2642565 | 13240 | Defa6 | 2.713 | 8.455 | 2.27E-13 | adenocarcinoma |
| ILMN_2793522 | 29820 | Tnfrsf19 | 2.007 | 8.311 | 2.33E-13 | adenocarcinoma |
| ILMN_2634670 | 17288 | Mep1b | -2.315 | 10.130 | 2.35E-13 | adenocarcinoma |
| ILMN_1247267 | 228608 | Smox | 2.091 | 10.790 | 2.51E-13 | adenocarcinoma |
| ILMN_2777363 | 21818 | Tgm3 | -3.468 | 11.026 | 2.63E-13 | adenocarcinoma |
| ILMN_2684600 | 101613 | Nlrp6 | -2.892 | 11.174 | 2.63E-13 | adenocarcinoma |
| ILMN_2725134 | 239853 | Gpr128 | -2.302 | 10.942 | 2.64E-13 | adenocarcinoma |
| ILMN_2607786 | 76267 | Fads1 | 2.408 | 11.150 | 2.67E-13 | adenocarcinoma |
| ILMN_2708203 | 12577 | Cdkn1c | 3.073 | 11.156 | 2.67E-13 | adenocarcinoma |
| ILMN_1217493 | 13639 | Efna4 | 1.463 | 8.660 | 2.76E-13 | adenocarcinoma |
| ILMN_2624180 | 66805 | Tspan1 | -2.174 | 11.605 | 2.76E-13 | adenocarcinoma |
| ILMN_2629582 | 18792 | Plau | 3.036 | 10.286 | 2.94E-13 | adenocarcinoma |
| ILMN_2667384 | 14664 | Slc6a9 | 2.275 | 10.311 | 2.95E-13 | adenocarcinoma |
| ILMN_2834563 | 66957 | Serpinb11 | 1.915 | 8.328 | 3.13E-13 | adenocarcinoma |
| ILMN_2792485 | 218793 | Ube2e2 | 1.507 | 10.931 | 3.14E-13 | adenocarcinoma |
| ILMN_2791952 | 55927 | Hes6 | 2.324 | 11.656 | 3.17E-13 | adenocarcinoma |
| ILMN_3074259 | 16784 | Lamp2 | 2.050 | 12.191 | 3.25E-13 | adenocarcinoma |
| ILMN_3147331 | 232078 | Thnsl2 | -1.866 | 10.277 | 3.27E-13 | adenocarcinoma |
| ILMN_1238069 | 207819 | 4930539E08Rik | -2.788 | 12.726 | 3.27E-13 | adenocarcinoma |
| ILMN_1248367 | 268860 | Abat | -3.008 | 11.648 | 3.44E-13 | adenocarcinoma |
| ILMN_3042923 | 381334 | Gal3st2 | -2.145 | 12.066 | 3.47E-13 | adenocarcinoma |
| ILMN_2595282 | 72462 | Rrp1b | 1.803 | 9.370 | 3.57E-13 | adenocarcinoma |
| ILMN_2636536 | 66350 | Pla2g12a | 2.713 | 10.912 | 3.59E-13 | adenocarcinoma |
| ILMN_1254943 | 233552 | Gdpd5 | 1.287 | 8.818 | 3.76E-13 | adenocarcinoma |
| ILMN_2461172 | 217316 | Slc16a5 | -2.561 | 10.302 | 3.76E-13 | adenocarcinoma |
| ILMN_2757634 | 104943 | Fam110c | -1.617 | 10.616 | 3.87E-13 | adenocarcinoma |
| ILMN_1248727 | NA | NA | 1.786 | 9.673 | 3.87E-13 | adenocarcinoma |
| ILMN_1247220 | 21386 | Tbx3 | 2.998 | 8.803 | 3.92E-13 | adenocarcinoma |
| ILMN_2636005 | 78748 | Rassf10 | 1.433 | 8.181 | 3.93E-13 | adenocarcinoma |
| ILMN_1239800 | 382097 | Gm1123 | -3.445 | 11.666 | 3.95E-13 | adenocarcinoma |
| ILMN_2492944 | 225631 | Onecut2 | 3.024 | 8.574 | 3.95E-13 | adenocarcinoma |
| ILMN_2680054 | 27528 | Nrep | 2.628 | 9.809 | 3.97E-13 | adenocarcinoma |
| ILMN_2479681 | 72640 | Mex3a | 2.175 | 8.484 | 4.28E-13 | adenocarcinoma |
| ILMN_1225921 | 394432 | Ugt1a7c | -2.490 | 11.213 | 4.28E-13 | adenocarcinoma |
| ILMN_1232668 | 17119 | Mxd1 | -2.247 | 11.374 | 4.28E-13 | adenocarcinoma |
| ILMN_3009685 | 19280 | Ptprs | 2.583 | 10.584 | 4.28E-13 | adenocarcinoma |
| ILMN_1229743 | 212307 | Mapre2 | -1.925 | 10.689 | 4.44E-13 | adenocarcinoma |
| ILMN_2599659 | 22379 | Fmnl3 | 1.721 | 8.740 | 4.49E-13 | adenocarcinoma |
| ILMN_2529395 | 269529 | Fbxo10 | 2.355 | 9.815 | 4.52E-13 | adenocarcinoma |
| ILMN_1251065 | 69064 | Fuom | -2.098 | 10.909 | 4.65E-13 | adenocarcinoma |
| ILMN_1237378 | 20254 | Scg2 | -1.315 | 8.874 | 4.65E-13 | adenocarcinoma |
| ILMN_1229056 | 27999 | Fam3c | 1.467 | 10.667 | 4.68E-13 | adenocarcinoma |
| ILMN_2649333 | 238323 | Rps6kl1 | 1.681 | 8.358 | 5.02E-13 | adenocarcinoma |
| ILMN_2677408 | 15469 | Prmt1 | 2.120 | 11.464 | 5.05E-13 | adenocarcinoma |
| ILMN_2603081 | 107569 | Nt5c3 | -1.708 | 12.329 | 5.05E-13 | adenocarcinoma |
| ILMN_2838501 | 94249 | Slc24a3 | -2.266 | 10.376 | 5.06E-13 | adenocarcinoma |
| ILMN_2865047 | 382097 | Gm1123 | -2.745 | 10.904 | 5.38E-13 | adenocarcinoma |
| ILMN_2623947 | 11758 | Prdx6 | -1.657 | 9.154 | 5.38E-13 | adenocarcinoma |
| ILMN_2504886 | 77583 | Notum | 1.741 | 8.284 | 5.38E-13 | adenocarcinoma |
| ILMN_3107945 | 76380 | Cep112 | 2.650 | 8.922 | 5.62E-13 | adenocarcinoma |
| ILMN_2451202 | 21909 | Tlx2 | 1.599 | 8.338 | 5.78E-13 | adenocarcinoma |
| ILMN_2828420 | 74340 | Ahcyl2 | -2.085 | 11.515 | 5.84E-13 | adenocarcinoma |
| ILMN_2694955 | 16010 | Igfbp4 | 2.869 | 10.348 | 5.84E-13 | adenocarcinoma |
| ILMN_1218051 | 544963 | Iqgap2 | -2.147 | 11.808 | 5.91E-13 | adenocarcinoma |
| ILMN_2728134 | 319909 | Ism1 | 2.695 | 10.014 | 5.98E-13 | adenocarcinoma |
| ILMN_1224256 | 226144 | Erlin1 | -1.618 | 10.659 | 5.98E-13 | adenocarcinoma |
| ILMN_2661340 | 108115 | Slco4a1 | 1.779 | 8.712 | 6.21E-13 | adenocarcinoma |
| ILMN_2963091 | 103551 | E130012A19Rik | 2.027 | 8.480 | 6.43E-13 | adenocarcinoma |
| ILMN_2946288 | 16532 | Kcnu1 | 1.297 | 8.232 | 6.49E-13 | adenocarcinoma |
| ILMN_1240289 | 231668 | Vsig10 | -1.388 | 10.507 | 6.61E-13 | adenocarcinoma |
| ILMN_2969845 | NA | NA | -2.597 | 9.770 | 6.65E-13 | adenocarcinoma |
| ILMN_2769136 | 17768 | Mthfd2 | 1.779 | 9.316 | 6.72E-13 | adenocarcinoma |
| ILMN_2713686 | 103711 | Pnpo | -1.575 | 9.459 | 6.78E-13 | adenocarcinoma |
| ILMN_2623735 | 70266 | Ccbl1 | -2.197 | 11.300 | 7.11E-13 | adenocarcinoma |
| ILMN_2771034 | 17304 | Mfge8 | 2.669 | 10.412 | 7.19E-13 | adenocarcinoma |
| ILMN_2932964 | 51797 | Ctps | 1.613 | 9.648 | 7.19E-13 | adenocarcinoma |
| ILMN_2632034 | 217946 | Cdca7l | 1.943 | 8.930 | 7.21E-13 | adenocarcinoma |
| ILMN_2474052 | NA | NA | 2.156 | 10.486 | 7.21E-13 | adenocarcinoma |
| ILMN_1225376 | 64075 | Smoc1 | 1.884 | 8.585 | 7.21E-13 | adenocarcinoma |
| ILMN_2783997 | 19824 | Trim10 | 2.101 | 8.219 | 7.29E-13 | adenocarcinoma |
| ILMN_2646369 | 20500 | Slc13a2 | -3.681 | 12.167 | 7.29E-13 | adenocarcinoma |
| ILMN_2606152 | NA | NA | 2.469 | 8.426 | 7.29E-13 | adenocarcinoma |
| ILMN_2961216 | 24059 | Slco2a1 | -1.986 | 10.916 | 7.36E-13 | adenocarcinoma |
| ILMN_2919786 | 13877 | Erh | 1.342 | 12.225 | 7.40E-13 | adenocarcinoma |
| ILMN_2595283 | 72462 | Rrp1b | 1.676 | 9.186 | 7.43E-13 | adenocarcinoma |
| ILMN_2873444 | 67731 | Fbxo32 | -2.088 | 11.357 | 7.56E-13 | adenocarcinoma |
| ILMN_2820305 | 17288 | Mep1b | -2.156 | 10.679 | 7.63E-13 | adenocarcinoma |
| ILMN_2758264 | 74134 | Cyp2s1 | -2.500 | 11.071 | 7.63E-13 | adenocarcinoma |
| ILMN_1234487 | 11601 | Angpt2 | 2.251 | 9.554 | 7.78E-13 | adenocarcinoma |
| ILMN_2739384 | 216225 | Slc5a8 | -3.565 | 12.811 | 7.78E-13 | adenocarcinoma |
| ILMN_2664660 | 214579 | Aldh5a1 | 1.580 | 10.065 | 8.03E-13 | adenocarcinoma |
| ILMN_1249766 | 14472 | Gbx2 | 3.090 | 8.625 | 8.22E-13 | adenocarcinoma |
| ILMN_1245335 | 240055 | Neurl1b | -1.378 | 9.306 | 8.28E-13 | adenocarcinoma |
| ILMN_1240247 | 69718 | Ipmk | -1.848 | 10.934 | 8.45E-13 | adenocarcinoma |
| ILMN_1259561 | 19072 | Prep | 1.468 | 10.112 | 8.50E-13 | adenocarcinoma |
| ILMN_2741132 | 15312 | Hmgn1 | 1.715 | 12.368 | 8.88E-13 | adenocarcinoma |
| ILMN_1220763 | 19752 | Rnase1 | 2.050 | 8.450 | 8.89E-13 | adenocarcinoma |
| ILMN_2677921 | 246154 | Vasn | 1.642 | 9.460 | 8.98E-13 | adenocarcinoma |
| ILMN_1250201 | 230991 | B930041F14Rik | 2.650 | 10.003 | 8.98E-13 | adenocarcinoma |
| ILMN_2678466 | NA | NA | -1.855 | 10.335 | 9.23E-13 | adenocarcinoma |
| ILMN_2686745 | 71729 | Rgs12 | 1.608 | 8.521 | 9.40E-13 | adenocarcinoma |
| ILMN_1227458 | 77976 | Nuak1 | 1.386 | 8.944 | 9.40E-13 | adenocarcinoma |
| ILMN_2482178 | 433904 | Ociad2 | -1.899 | 11.865 | 9.40E-13 | adenocarcinoma |
| ILMN_2597030 | 228608 | Smox | 2.058 | 10.463 | 9.81E-13 | adenocarcinoma |
| ILMN_2687744 | 70510 | Rnf167 | -1.388 | 10.175 | 9.90E-13 | adenocarcinoma |
| ILMN_3031099 | 67876 | Coq10b | -2.167 | 11.095 | 9.95E-13 | adenocarcinoma |
| ILMN_1213989 | 65112 | Pmepa1 | 1.847 | 8.767 | 1.03E-12 | adenocarcinoma |
| ILMN_1244829 | 15114 | Hap1 | 2.321 | 9.260 | 1.05E-12 | adenocarcinoma |
| ILMN_1226606 | 98170 | Tmem132a | 1.568 | 8.334 | 1.06E-12 | adenocarcinoma |
| ILMN_1214800 | 70510 | Rnf167 | -1.551 | 10.629 | 1.06E-12 | adenocarcinoma |
| ILMN_2773485 | 232078 | Thnsl2 | -1.905 | 10.177 | 1.10E-12 | adenocarcinoma |
| ILMN_2961005 | 72500 | Ier5l | 1.728 | 8.818 | 1.10E-12 | adenocarcinoma |
| ILMN_2925424 | 239606 | Slc2a13 | -1.910 | 9.995 | 1.11E-12 | adenocarcinoma |
| ILMN_2787212 | 18025 | Nfe2l3 | 1.388 | 8.334 | 1.11E-12 | adenocarcinoma |
| ILMN_2618176 | 12702 | Socs3 | 2.329 | 9.388 | 1.11E-12 | adenocarcinoma |
| ILMN_1236245 | NA | NA | -1.891 | 9.597 | 1.11E-12 | adenocarcinoma |
| ILMN_3072957 | 72640 | Mex3a | 1.426 | 8.395 | 1.12E-12 | adenocarcinoma |
| ILMN_2923607 | 27280 | Phlda3 | 2.101 | 8.980 | 1.16E-12 | adenocarcinoma |
| ILMN_1228582 | 192113 | Atp12a | -4.440 | 12.634 | 1.18E-12 | adenocarcinoma |
| ILMN_1214117 | NA | NA | 1.588 | 8.244 | 1.18E-12 | adenocarcinoma |
| ILMN_2734181 | 60525 | Acss2 | -1.567 | 12.969 | 1.19E-12 | adenocarcinoma |
| ILMN_2647234 | 13370 | Dio1 | -1.679 | 9.320 | 1.22E-12 | adenocarcinoma |
| ILMN_1229534 | 70510 | Rnf167 | -1.280 | 10.082 | 1.25E-12 | adenocarcinoma |
| ILMN_2940713 | 380711 | Rap1gap2 | -1.838 | 11.127 | 1.29E-12 | adenocarcinoma |
| ILMN_3104462 | 67876 | Coq10b | -1.694 | 11.245 | 1.29E-12 | adenocarcinoma |
| ILMN_1252243 | 68294 | Mfsd10 | 1.827 | 10.287 | 1.30E-12 | adenocarcinoma |
| ILMN_2752371 | 209378 | Itih5 | -1.407 | 9.089 | 1.36E-12 | adenocarcinoma |
| ILMN_1253362 | 93721 | Cpn1 | 1.811 | 8.393 | 1.39E-12 | adenocarcinoma |
| ILMN_1230440 | 73338 | Itpripl1 | 2.423 | 9.481 | 1.53E-12 | adenocarcinoma |
| ILMN_2755492 | 27999 | Fam3c | 1.459 | 10.213 | 1.55E-12 | adenocarcinoma |
| ILMN_2987339 | 18432 | Mybbp1a | 1.961 | 10.031 | 1.57E-12 | adenocarcinoma |
| ILMN_2758717 | 235527 | Plscr4 | -2.210 | 10.127 | 1.58E-12 | adenocarcinoma |
| ILMN_1231625 | 64385 | Cyp4f14 | -3.290 | 12.477 | 1.58E-12 | adenocarcinoma |
| ILMN_2729799 | 226999 | Slc9a2 | -1.861 | 9.638 | 1.64E-12 | adenocarcinoma |
| ILMN_2606130 | 69837 | Pcgf1 | 1.882 | 10.535 | 1.64E-12 | adenocarcinoma |
| ILMN_2622217 | 17880 | Myh11 | -2.759 | 11.260 | 1.68E-12 | adenocarcinoma |
| ILMN_2531741 | 67160 | Eef1g | 1.322 | 12.684 | 1.77E-12 | adenocarcinoma |
| ILMN_2599657 | 22379 | Fmnl3 | 1.442 | 8.379 | 1.77E-12 | adenocarcinoma |
| ILMN_1226970 | 66894 | Wwp2 | -1.732 | 11.174 | 1.77E-12 | adenocarcinoma |
| ILMN_2736197 | 72512 | Tmem173 | 1.824 | 8.667 | 1.77E-12 | adenocarcinoma |
| ILMN_2492264 | 22402 | Wisp1 | 1.640 | 8.802 | 1.77E-12 | adenocarcinoma |
| ILMN_1224211 | NA | NA | 1.415 | 8.490 | 1.82E-12 | adenocarcinoma |
| ILMN_1252402 | 21676 | Tead1 | 1.767 | 9.865 | 1.89E-12 | adenocarcinoma |
| ILMN_1220166 | NA | NA | -2.051 | 10.375 | 2.02E-12 | adenocarcinoma |
| ILMN_2641613 | 20588 | Smarcc1 | 1.375 | 8.971 | 2.14E-12 | adenocarcinoma |
| ILMN_1257088 | 19142 | Prss12 | 1.386 | 8.275 | 2.14E-12 | adenocarcinoma |
| ILMN_2651054 | 269589 | Sytl1 | -1.707 | 11.237 | 2.20E-12 | adenocarcinoma |
| ILMN_2810473 | 11364 | Acadm | -1.383 | 13.456 | 2.24E-12 | adenocarcinoma |
| ILMN_1215147 | 14113 | Fbl | 2.024 | 11.337 | 2.25E-12 | adenocarcinoma |
| ILMN_2751509 | 223706 | Cyp2d34 | -2.316 | 9.869 | 2.25E-12 | adenocarcinoma |
| ILMN_2687507 | 13105 | Cyp2d9 | -3.220 | 11.045 | 2.32E-12 | adenocarcinoma |
| ILMN_2733605 | 72685 | Dnajc6 | 2.009 | 8.637 | 2.33E-12 | adenocarcinoma |
| ILMN_2888842 | 69046 | Isca1 | -1.297 | 11.954 | 2.36E-12 | adenocarcinoma |
| ILMN_1252310 | 13436 | Dnmt3b | 1.561 | 9.348 | 2.37E-12 | adenocarcinoma |
| ILMN_2648409 | 102545 | Cmtm7 | 1.896 | 9.179 | 2.37E-12 | adenocarcinoma |
| ILMN_1221067 | 18102 | Nme1 | 2.009 | 13.061 | 2.45E-12 | adenocarcinoma |
| ILMN_3161105 | NA | NA | -1.537 | 10.258 | 2.52E-12 | adenocarcinoma |
| ILMN_2903945 | 23882 | Gadd45g | 1.907 | 10.883 | 2.53E-12 | adenocarcinoma |
| ILMN_1226517 | NA | NA | 1.561 | 9.957 | 2.67E-12 | adenocarcinoma |
| ILMN_2713071 | 56473 | Fads2 | -1.812 | 10.904 | 2.73E-12 | adenocarcinoma |
| ILMN_2599402 | 21885 | Tle1 | 1.556 | 9.253 | 2.75E-12 | adenocarcinoma |
| ILMN_2648548 | 12369 | Casp7 | -1.817 | 11.325 | 2.76E-12 | adenocarcinoma |
| ILMN_2638114 | 19242 | Ptn | 3.187 | 10.514 | 2.98E-12 | adenocarcinoma |
| ILMN_2687372 | 76608 | Hectd3 | -1.191 | 10.477 | 3.01E-12 | adenocarcinoma |
| ILMN_2735184 | 12822 | Col18a1 | 2.824 | 10.465 | 3.09E-12 | adenocarcinoma |
| ILMN_2931411 | 12462 | Cct3 | 1.139 | 11.939 | 3.11E-12 | adenocarcinoma |
| ILMN_1233339 | 56520 | Nme4 | 1.927 | 9.014 | 3.25E-12 | adenocarcinoma |
| ILMN_2856567 | 19064 | Ppy | -2.018 | 10.338 | 3.47E-12 | adenocarcinoma |
| ILMN_2506489 | 215690 | Nav1 | 1.466 | 8.554 | 3.52E-12 | adenocarcinoma |
| ILMN_2705361 | 114664 | Hsd17b11 | -1.514 | 11.643 | 3.52E-12 | adenocarcinoma |
| ILMN_1227225 | 21849 | Trim28 | 1.901 | 11.253 | 3.61E-12 | adenocarcinoma |
| ILMN_1221373 | NA | NA | -2.026 | 10.517 | 3.80E-12 | adenocarcinoma |
| ILMN_2690596 | 15213 | Hey1 | 1.319 | 8.707 | 3.88E-12 | adenocarcinoma |
| ILMN_2737090 | 66184 | Rps4l | -1.595 | 8.432 | 4.13E-12 | adenocarcinoma |
| ILMN_2618794 | 68927 | Ptcd2 | -1.079 | 10.278 | 4.15E-12 | adenocarcinoma |
| ILMN_2599719 | 68682 | Slc44a2 | 1.513 | 10.609 | 4.24E-12 | adenocarcinoma |
| ILMN_2653567 | 80837 | Rhoj | 2.607 | 10.472 | 4.24E-12 | adenocarcinoma |
| ILMN_1252338 | 545123 | Cyp2d11 | -2.596 | 10.596 | 4.25E-12 | adenocarcinoma |
| ILMN_2734252 | 15203 | Heph | -1.801 | 11.498 | 4.34E-12 | adenocarcinoma |
| ILMN_2606719 | 12780 | Abcc2 | 1.240 | 8.345 | 4.39E-12 | adenocarcinoma |
| ILMN_2943722 | 433904 | Ociad2 | -1.964 | 11.908 | 4.40E-12 | adenocarcinoma |
| ILMN_2589662 | 12162 | Bmp7 | 1.833 | 8.498 | 4.40E-12 | adenocarcinoma |
| ILMN_2844996 | 109711 | Actn1 | 1.455 | 11.672 | 4.93E-12 | adenocarcinoma |
| ILMN_1241225 | 320685 | Dctd | 1.454 | 8.984 | 4.96E-12 | adenocarcinoma |
| ILMN_2984828 | 17069 | Ly6e | 2.804 | 9.374 | 5.06E-12 | adenocarcinoma |
| ILMN_2971688 | 211323 | Nrg1 | 1.570 | 8.561 | 5.10E-12 | adenocarcinoma |
| ILMN_1250000 | 56398 | Chp1 | -1.787 | 10.028 | 5.24E-12 | adenocarcinoma |
| ILMN_2789862 | 330671 | B4galnt4 | 1.509 | 8.122 | 5.28E-12 | adenocarcinoma |
| ILMN_1250696 | 21983 | Tpbg | 1.797 | 9.475 | 5.31E-12 | adenocarcinoma |
| ILMN_1232707 | 56045 | Samhd1 | -2.060 | 9.579 | 5.41E-12 | adenocarcinoma |
| ILMN_2680628 | 67307 | Pbld2 | -2.220 | 10.586 | 5.45E-12 | adenocarcinoma |
| ILMN_2652458 | 98932 | Myl9 | -2.413 | 10.513 | 5.54E-12 | adenocarcinoma |
| ILMN_1227164 | 22154 | Tubb5 | 2.582 | 13.384 | 5.55E-12 | adenocarcinoma |
| ILMN_1217454 | 16784 | Lamp2 | 1.970 | 12.903 | 5.61E-12 | adenocarcinoma |
| ILMN_1243652 | 21345 | Tagln | -2.335 | 10.035 | 5.66E-12 | adenocarcinoma |
| ILMN_2790357 | 20361 | Sema7a | 2.489 | 8.599 | 5.75E-12 | adenocarcinoma |
| ILMN_2883392 | NA | NA | 1.991 | 12.950 | 6.06E-12 | adenocarcinoma |
| ILMN_2427869 | 71242 | Spata24 | 1.232 | 9.289 | 6.10E-12 | adenocarcinoma |
| ILMN_2736539 | 72082 | Cyp2c55 | -3.902 | 12.582 | 6.66E-12 | adenocarcinoma |
| ILMN_1213026 | 226744 | Cnst | -1.132 | 10.551 | 6.71E-12 | adenocarcinoma |
| ILMN_1234773 | 69787 | Anxa13 | -1.292 | 8.892 | 6.76E-12 | adenocarcinoma |
| ILMN_1225056 | 330064 | Slc5a6 | -1.677 | 11.143 | 6.83E-12 | adenocarcinoma |
| ILMN_2689790 | 16012 | Igfbp6 | -1.963 | 9.503 | 7.07E-12 | adenocarcinoma |
| ILMN_1255728 | 19167 | Psma3 | 1.788 | 10.118 | 7.14E-12 | adenocarcinoma |
| ILMN_1224336 | 74646 | Spsb1 | 1.369 | 9.465 | 7.14E-12 | adenocarcinoma |
| ILMN_2527796 | 100043899 | R3hdml | 1.379 | 8.132 | 7.24E-12 | adenocarcinoma |
| ILMN_2785350 | 171095 | Il17rc | -1.732 | 11.758 | 7.24E-12 | adenocarcinoma |
| ILMN_1248293 | 22601 | Yap1 | 1.380 | 9.673 | 7.36E-12 | adenocarcinoma |
| ILMN_2986051 | 70266 | Ccbl1 | -2.130 | 11.895 | 7.59E-12 | adenocarcinoma |
| ILMN_2744890 | 23882 | Gadd45g | 1.336 | 8.963 | 7.59E-12 | adenocarcinoma |
| ILMN_2738345 | 225341 | Lims2 | -1.780 | 9.861 | 7.70E-12 | adenocarcinoma |
| ILMN_2595593 | 17178 | Fxyd3 | 1.643 | 10.323 | 7.81E-12 | adenocarcinoma |
| ILMN_2839745 | 319278 | A230050P20Rik | 1.940 | 9.772 | 7.90E-12 | adenocarcinoma |
| ILMN_1226104 | 18260 | Ocln | -1.442 | 10.506 | 8.36E-12 | adenocarcinoma |
| ILMN_2850077 | 11522 | Adh1 | -2.917 | 15.027 | 8.37E-12 | adenocarcinoma |
| ILMN_2727546 | 97775 | D930048N14Rik | 2.100 | 9.487 | 8.37E-12 | adenocarcinoma |
| ILMN_1253986 | 71228 | Dlg5 | 2.114 | 10.577 | 8.37E-12 | adenocarcinoma |
| ILMN_3163306 | 223227 | Sox21 | 1.504 | 8.450 | 8.38E-12 | adenocarcinoma |
| ILMN_2677472 | 18230 | Nxn | 1.997 | 10.868 | 8.67E-12 | adenocarcinoma |
| ILMN_1255766 | 24055 | Sh3bp2 | 1.512 | 9.319 | 8.76E-12 | adenocarcinoma |
| ILMN_1231713 | NA | NA | 1.273 | 8.132 | 8.84E-12 | adenocarcinoma |
| ILMN_2937122 | 67095 | Trak1 | -1.679 | 10.635 | 8.87E-12 | adenocarcinoma |
| ILMN_2616682 | 19142 | Prss12 | 1.276 | 8.081 | 8.87E-12 | adenocarcinoma |
| ILMN_1214880 | 233335 | Synm | -2.108 | 10.349 | 8.96E-12 | adenocarcinoma |
| ILMN_1243900 | 223650 | Eppk1 | -1.456 | 11.714 | 9.02E-12 | adenocarcinoma |
| ILMN_3068231 | 17187 | Max | -1.706 | 9.917 | 9.02E-12 | adenocarcinoma |
| ILMN_2972478 | 216856 | Nlgn2 | 1.370 | 8.379 | 9.06E-12 | adenocarcinoma |
| ILMN_1232533 | 68416 | Sycn | -3.337 | 14.970 | 9.25E-12 | adenocarcinoma |
| ILMN_2472730 | 241494 | Zfp385b | -2.013 | 9.193 | 9.45E-12 | adenocarcinoma |
| ILMN_2422982 | 104027 | Synpo | -1.683 | 11.831 | 9.52E-12 | adenocarcinoma |
| ILMN_2737758 | 19280 | Ptprs | 1.245 | 8.602 | 9.69E-12 | adenocarcinoma |
| ILMN_1258501 | 11522 | Adh1 | -3.367 | 13.978 | 9.73E-12 | adenocarcinoma |
| ILMN_2772174 | 235036 | Ppan | 2.006 | 9.964 | 9.73E-12 | adenocarcinoma |
| ILMN_1225764 | 76574 | Mfsd2a | 2.675 | 8.532 | 9.73E-12 | adenocarcinoma |
| ILMN_1231503 | 67041 | Oxct1 | -2.314 | 12.578 | 1.00E-11 | adenocarcinoma |
| ILMN_1236358 | 107932 | Chd4 | 1.632 | 10.109 | 1.02E-11 | adenocarcinoma |
| ILMN_2745930 | 56370 | Tagln3 | 1.638 | 8.625 | 1.06E-11 | adenocarcinoma |
| ILMN_3111685 | 20216 | Acsm3 | -2.716 | 12.510 | 1.06E-11 | adenocarcinoma |
| ILMN_2907642 | 104458 | Rars | 1.156 | 10.972 | 1.07E-11 | adenocarcinoma |
| ILMN_2702903 | 13107 | Cyp2f2 | -2.854 | 10.519 | 1.07E-11 | adenocarcinoma |
| ILMN_2903718 | 100503970 | AY761185 | 2.812 | 8.521 | 1.08E-11 | adenocarcinoma |
| ILMN_2494618 | 216825 | Usp22 | 1.514 | 10.015 | 1.08E-11 | adenocarcinoma |
| ILMN_2960467 | 18674 | Slc25a3 | -1.298 | 11.251 | 1.08E-11 | adenocarcinoma |
| ILMN_2503361 | 73192 | Xpot | 1.895 | 10.456 | 1.09E-11 | adenocarcinoma |
| ILMN_1214811 | 19164 | Psen1 | -1.363 | 11.392 | 1.09E-11 | adenocarcinoma |
| ILMN_2740152 | 212862 | Chpt1 | -1.448 | 10.053 | 1.10E-11 | adenocarcinoma |
| ILMN_3029727 | 435684 | Shf | 1.938 | 9.146 | 1.11E-11 | adenocarcinoma |
| ILMN_2908070 | 29870 | Gtse1 | 2.018 | 8.776 | 1.11E-11 | adenocarcinoma |
| ILMN_1230878 | 15039 | H2-T22 | -1.172 | 9.017 | 1.12E-11 | adenocarcinoma |
| ILMN_2886828 | 218772 | Rarb | 1.737 | 8.471 | 1.12E-11 | adenocarcinoma |
| ILMN_2417611 | 71661 | 0610005C13Rik | -1.388 | 8.580 | 1.13E-11 | adenocarcinoma |
| ILMN_1257340 | 26992 | Brd7 | 1.087 | 10.176 | 1.17E-11 | adenocarcinoma |
| ILMN_2467292 | 239606 | Slc2a13 | -2.129 | 10.511 | 1.18E-11 | adenocarcinoma |
| ILMN_2867835 | 17319 | Mif | 1.720 | 14.220 | 1.18E-11 | adenocarcinoma |
| ILMN_2696749 | 170758 | Rac3 | 1.172 | 8.530 | 1.18E-11 | adenocarcinoma |
| ILMN_2718662 | 98932 | Myl9 | -2.870 | 11.348 | 1.18E-11 | adenocarcinoma |
| ILMN_1233809 | 102545 | Cmtm7 | 1.791 | 9.464 | 1.22E-11 | adenocarcinoma |
| ILMN_3043587 | NA | NA | 2.013 | 8.894 | 1.23E-11 | adenocarcinoma |
| ILMN_1225191 | 223646 | Naprt1 | -1.617 | 12.576 | 1.25E-11 | adenocarcinoma |
| ILMN_1221243 | 73296 | Rhobtb3 | 1.426 | 9.006 | 1.27E-11 | adenocarcinoma |
| ILMN_1244093 | 69814 | Prss32 | -2.046 | 12.899 | 1.27E-11 | adenocarcinoma |
| ILMN_2760272 | 19885 | Rorc | -1.783 | 9.811 | 1.27E-11 | adenocarcinoma |
| ILMN_2450767 | NA | NA | 1.747 | 9.065 | 1.27E-11 | adenocarcinoma |
| ILMN_1231836 | 272636 | Esyt3 | 2.097 | 8.854 | 1.27E-11 | adenocarcinoma |
| ILMN_2614853 | 328162 | Trmt61a | 1.375 | 9.640 | 1.31E-11 | adenocarcinoma |
| ILMN_2776283 | 21401 | Tcea3 | -2.060 | 11.403 | 1.32E-11 | adenocarcinoma |
| ILMN_2695085 | 81840 | Sorcs2 | 1.155 | 8.324 | 1.32E-11 | adenocarcinoma |
| ILMN_3138046 | 225997 | Trpm6 | -1.519 | 9.610 | 1.33E-11 | adenocarcinoma |
| ILMN_2710455 | 56016 | Hebp2 | 1.140 | 8.954 | 1.33E-11 | adenocarcinoma |
| ILMN_2930203 | NA | NA | -2.622 | 12.202 | 1.35E-11 | adenocarcinoma |
| ILMN_2680533 | 14871 | Gstt1 | -1.430 | 9.702 | 1.35E-11 | adenocarcinoma |
| ILMN_2500370 | 26921 | Map4k4 | 1.846 | 11.109 | 1.35E-11 | adenocarcinoma |
| ILMN_1235433 | 110075 | Bmp3 | -2.066 | 9.854 | 1.37E-11 | adenocarcinoma |
| ILMN_1232578 | NA | NA | 2.469 | 8.489 | 1.38E-11 | adenocarcinoma |
| ILMN_2677634 | 224796 | Clic5 | -1.951 | 9.565 | 1.38E-11 | adenocarcinoma |
| ILMN_2934534 | 28000 | Prpf19 | 1.406 | 10.776 | 1.38E-11 | adenocarcinoma |
| ILMN_1232263 | 16529 | Kcnk5 | -1.473 | 9.371 | 1.41E-11 | adenocarcinoma |
| ILMN_1215055 | 21841 | Tia1 | 1.311 | 9.292 | 1.44E-11 | adenocarcinoma |
| ILMN_2605666 | 16784 | Lamp2 | 2.216 | 11.673 | 1.45E-11 | adenocarcinoma |
| ILMN_1258636 | 27062 | Cadps | 2.640 | 8.926 | 1.45E-11 | adenocarcinoma |
| ILMN_2588398 | NA | NA | 1.505 | 11.260 | 1.45E-11 | adenocarcinoma |
| ILMN_2803920 | 17068 | Ly6d | 2.619 | 8.698 | 1.51E-11 | adenocarcinoma |
| ILMN_2618795 | 68927 | Ptcd2 | -1.031 | 10.245 | 1.52E-11 | adenocarcinoma |
| ILMN_1226280 | 68147 | Gar1 | 2.203 | 11.305 | 1.57E-11 | adenocarcinoma |
| ILMN_1253579 | 330671 | B4galnt4 | 1.144 | 8.142 | 1.60E-11 | adenocarcinoma |
| ILMN_2725719 | 243373 | AI854703 | -2.577 | 11.181 | 1.61E-11 | adenocarcinoma |
| ILMN_2454823 | NA | NA | 1.351 | 8.030 | 1.62E-11 | adenocarcinoma |
| ILMN_1232884 | 20698 | Sphk1 | 1.463 | 8.841 | 1.62E-11 | adenocarcinoma |
| ILMN_2976821 | 93897 | Fzd10 | 2.604 | 8.728 | 1.63E-11 | adenocarcinoma |
| ILMN_2735429 | 232023 | Vopp1 | 1.378 | 10.053 | 1.64E-11 | adenocarcinoma |
| ILMN_3136205 | 50790 | Acsl4 | 1.019 | 8.379 | 1.65E-11 | adenocarcinoma |
| ILMN_2608429 | 11972 | Atp6v0d1 | -1.442 | 10.720 | 1.65E-11 | adenocarcinoma |
| ILMN_2656504 | 245195 | Retnlg | 1.589 | 8.252 | 1.70E-11 | adenocarcinoma |
| ILMN_1232435 | 70945 | Mmrn1 | -1.504 | 9.189 | 1.71E-11 | adenocarcinoma |
| ILMN_1228778 | NA | NA | 2.270 | 8.412 | 1.74E-11 | adenocarcinoma |
| ILMN_2979257 | 268480 | Rapgefl1 | -1.325 | 9.620 | 1.79E-11 | adenocarcinoma |
| ILMN_2601907 | 16825 | Ldb1 | 1.579 | 12.484 | 1.80E-11 | adenocarcinoma |
| ILMN_1229266 | 271305 | Phf21b | 1.886 | 8.987 | 1.92E-11 | adenocarcinoma |
| ILMN_2777884 | 13846 | Ephb4 | 1.556 | 10.864 | 1.92E-11 | adenocarcinoma |
| ILMN_2737710 | 21844 | Tiam1 | 1.687 | 8.744 | 1.95E-11 | adenocarcinoma |
| ILMN_2880536 | 80914 | Uck2 | 1.498 | 10.268 | 1.96E-11 | adenocarcinoma |
| ILMN_2420465 | 353310 | Zfp703 | 2.048 | 9.379 | 1.97E-11 | adenocarcinoma |
| ILMN_2852957 | 50781 | Dkk3 | 2.325 | 10.853 | 1.99E-11 | adenocarcinoma |
| ILMN_2482600 | 21934 | Tnfrsf11a | -1.344 | 10.227 | 2.00E-11 | adenocarcinoma |
| ILMN_1252131 | 16619 | Klk1b27 | -2.660 | 14.387 | 2.01E-11 | adenocarcinoma |
| ILMN_2777609 | 21400 | Tcea2 | 1.470 | 9.544 | 2.04E-11 | adenocarcinoma |
| ILMN_2435835 | 14027 | Evpl | -1.121 | 10.172 | 2.04E-11 | adenocarcinoma |
| ILMN_2426480 | NA | NA | -1.706 | 10.180 | 2.05E-11 | adenocarcinoma |
| ILMN_2958099 | 11565 | Adssl1 | 1.487 | 8.916 | 2.11E-11 | adenocarcinoma |
| ILMN_2644587 | 66912 | Bzw2 | 1.473 | 11.936 | 2.17E-11 | adenocarcinoma |
| ILMN_3143748 | 71148 | Mier1 | -1.381 | 11.198 | 2.19E-11 | adenocarcinoma |
| ILMN_2771709 | 170826 | Ppargc1b | -2.008 | 10.620 | 2.21E-11 | adenocarcinoma |
| ILMN_1213811 | 15486 | Hsd17b2 | -2.075 | 11.504 | 2.23E-11 | adenocarcinoma |
| ILMN_1259174 | 20259 | Scin | -2.984 | 13.884 | 2.25E-11 | adenocarcinoma |
| ILMN_2777319 | 20855 | Stc1 | 2.224 | 8.385 | 2.47E-11 | adenocarcinoma |
| ILMN_1246351 | NA | NA | -1.401 | 9.273 | 2.59E-11 | adenocarcinoma |
| ILMN_2430813 | 67399 | Pdlim7 | 1.798 | 10.772 | 2.61E-11 | adenocarcinoma |
| ILMN_2889832 | 546546 | Serpina3h | 2.551 | 9.415 | 2.61E-11 | adenocarcinoma |
| ILMN_2803138 | 108079 | Prkaa2 | -1.773 | 10.012 | 2.66E-11 | adenocarcinoma |
| ILMN_3088934 | 72657 | 2700094K13Rik | 1.844 | 11.967 | 2.69E-11 | adenocarcinoma |
| ILMN_2665008 | 106572 | Rab31 | 1.388 | 10.771 | 2.76E-11 | adenocarcinoma |
| ILMN_2740149 | 212862 | Chpt1 | -1.657 | 9.792 | 2.88E-11 | adenocarcinoma |
| ILMN_2715893 | 67731 | Fbxo32 | -1.720 | 10.302 | 2.88E-11 | adenocarcinoma |
| ILMN_1244853 | 100040259 | Gm16379 | 1.538 | 10.000 | 3.00E-11 | adenocarcinoma |
| ILMN_2689138 | 217732 | Cipc | -2.175 | 11.483 | 3.01E-11 | adenocarcinoma |
| ILMN_2606288 | 212070 | Clrn3 | -2.066 | 11.252 | 3.11E-11 | adenocarcinoma |
| ILMN_2945588 | 67203 | Nde1 | 1.032 | 8.435 | 3.13E-11 | adenocarcinoma |
| ILMN_2493338 | NA | NA | 2.414 | 10.573 | 3.17E-11 | adenocarcinoma |
| ILMN_1256359 | 228608 | Smox | 1.289 | 8.741 | 3.38E-11 | adenocarcinoma |
| ILMN_2772821 | 70423 | Tspan15 | -1.137 | 9.497 | 3.46E-11 | adenocarcinoma |
| ILMN_2868220 | 16323 | Inhba | 2.466 | 8.708 | 3.59E-11 | adenocarcinoma |
| ILMN_2712668 | 270198 | Pfkfb4 | -1.743 | 11.065 | 3.61E-11 | adenocarcinoma |
| ILMN_2903926 | 68797 | Pdgfrl | 1.259 | 8.338 | 3.69E-11 | adenocarcinoma |
| ILMN_2606746 | 12349 | Car2 | -2.405 | 12.709 | 3.72E-11 | adenocarcinoma |
| ILMN_2719069 | 109731 | Maob | -2.527 | 9.840 | 3.72E-11 | adenocarcinoma |
| ILMN_3107059 | 56226 | Espn | -1.180 | 11.970 | 3.88E-11 | adenocarcinoma |
| ILMN_2616479 | 17287 | Mep1a | -1.770 | 9.366 | 3.97E-11 | adenocarcinoma |
| ILMN_2744245 | 66894 | Wwp2 | -1.215 | 9.252 | 4.00E-11 | adenocarcinoma |
| ILMN_1245531 | 74513 | Neto2 | 1.239 | 8.155 | 4.00E-11 | adenocarcinoma |
| ILMN_2791355 | 76051 | Ganc | -1.474 | 10.210 | 4.12E-11 | adenocarcinoma |
| ILMN_2709047 | 64424 | Polr1e | 1.137 | 9.522 | 4.15E-11 | adenocarcinoma |
| ILMN_1251984 | NA | NA | 1.235 | 10.635 | 4.18E-11 | adenocarcinoma |
| ILMN_2938932 | 626708 | Defa26 | 2.713 | 8.574 | 4.20E-11 | adenocarcinoma |
| ILMN_3015387 | 432516 | Myo1a | -1.209 | 9.084 | 4.20E-11 | adenocarcinoma |
| ILMN_2544661 | NA | NA | -1.432 | 10.991 | 4.24E-11 | adenocarcinoma |
| ILMN_1253634 | 75723 | Amotl1 | 1.468 | 9.296 | 4.25E-11 | adenocarcinoma |
| ILMN_1237670 | 12496 | Entpd2 | -1.335 | 10.588 | 4.36E-11 | adenocarcinoma |
| ILMN_1259910 | 17978 | Ncoa2 | -1.250 | 9.547 | 4.41E-11 | adenocarcinoma |
| ILMN_2809611 | 20810 | Srm | 1.815 | 9.516 | 4.41E-11 | adenocarcinoma |
| ILMN_1220243 | 208177 | Phldb2 | 1.160 | 8.351 | 4.46E-11 | adenocarcinoma |
| ILMN_2827997 | 214189 | Scgn | -1.057 | 8.882 | 4.46E-11 | adenocarcinoma |
| ILMN_1255422 | 12457 | Ccrn4l | -1.546 | 10.740 | 4.47E-11 | adenocarcinoma |
| ILMN_1232593 | 70044 | Tut1 | 1.005 | 9.914 | 4.48E-11 | adenocarcinoma |
| ILMN_3162081 | 83669 | Wdr6 | 1.512 | 8.759 | 4.52E-11 | adenocarcinoma |
| ILMN_2663996 | 66825 | Rnf186 | -1.413 | 10.732 | 4.55E-11 | adenocarcinoma |
| ILMN_2775891 | 67781 | Ilf2 | 1.197 | 11.228 | 4.58E-11 | adenocarcinoma |
| ILMN_1250612 | 381605 | Tbc1d2 | -1.262 | 9.935 | 4.59E-11 | adenocarcinoma |
| ILMN_3108004 | 268663 | Cdhr2 | -1.646 | 12.023 | 4.60E-11 | adenocarcinoma |
| ILMN_2764883 | 17134 | Mafg | 1.902 | 10.440 | 4.61E-11 | adenocarcinoma |
| ILMN_2441642 | NA | NA | 2.003 | 8.525 | 4.63E-11 | adenocarcinoma |
| ILMN_2631704 | 12630 | Cfi | 2.462 | 8.717 | 4.64E-11 | adenocarcinoma |
| ILMN_1217054 | 70683 | Utp20 | -2.228 | 10.980 | 4.74E-11 | adenocarcinoma |
| ILMN_3160218 | 270152 | Amica1 | 1.901 | 9.536 | 4.83E-11 | adenocarcinoma |
| ILMN_2737193 | 53356 | Eif3g | 1.298 | 12.430 | 4.83E-11 | adenocarcinoma |
| ILMN_3105205 | 94184 | Pdxdc1 | -1.564 | 11.005 | 4.83E-11 | adenocarcinoma |
| ILMN_2711562 | 18719 | Pip5k1b | -1.451 | 11.090 | 4.84E-11 | adenocarcinoma |
| ILMN_2651575 | 15979 | Ifngr1 | -1.452 | 11.862 | 4.84E-11 | adenocarcinoma |
| ILMN_1248415 | 22134 | Tgoln1 | -1.856 | 11.754 | 4.90E-11 | adenocarcinoma |
| ILMN_2827081 | 27219 | Sgk2 | -2.094 | 10.255 | 5.08E-11 | adenocarcinoma |
| ILMN_1221470 | 74493 | Tnks2 | 1.102 | 11.296 | 5.09E-11 | adenocarcinoma |
| ILMN_2505841 | 54725 | Cadm1 | 1.464 | 8.689 | 5.09E-11 | adenocarcinoma |
| ILMN_1232537 | 320982 | Arl4c | 1.301 | 8.188 | 5.12E-11 | adenocarcinoma |
| ILMN_2803921 | 17068 | Ly6d | 2.096 | 8.575 | 5.13E-11 | adenocarcinoma |
| ILMN_1223416 | 226101 | Myof | 1.772 | 10.409 | 5.24E-11 | adenocarcinoma |
| ILMN_1247347 | 14526 | Gcg | -2.232 | 10.130 | 5.25E-11 | adenocarcinoma |
| ILMN_1244773 | 72657 | 2700094K13Rik | 1.870 | 10.463 | 5.38E-11 | adenocarcinoma |
| ILMN_2714638 | 17289 | Mertk | -1.626 | 9.838 | 5.40E-11 | adenocarcinoma |
| ILMN_2959292 | 22271 | Upp1 | -2.347 | 10.850 | 5.45E-11 | adenocarcinoma |
| ILMN_2464999 | 22248 | Unc119 | -1.357 | 9.784 | 5.52E-11 | adenocarcinoma |
| ILMN_1212801 | NA | NA | -1.251 | 9.039 | 5.54E-11 | adenocarcinoma |
| ILMN_2884728 | 21400 | Tcea2 | 1.398 | 9.454 | 5.59E-11 | adenocarcinoma |
| ILMN_1218956 | 21676 | Tead1 | 1.087 | 8.739 | 5.61E-11 | adenocarcinoma |
| ILMN_3101919 | 380921 | Dgkh | -1.228 | 9.182 | 5.62E-11 | adenocarcinoma |
| ILMN_2761046 | 70556 | Slc25a33 | 1.386 | 9.566 | 5.71E-11 | adenocarcinoma |
| ILMN_2833936 | 68631 | Cryl1 | -1.973 | 12.168 | 5.73E-11 | adenocarcinoma |
| ILMN_2956765 | 22335 | Vdac3 | -1.399 | 12.160 | 6.08E-11 | adenocarcinoma |
| ILMN_2689265 | 102632 | Acad11 | -1.189 | 8.885 | 6.09E-11 | adenocarcinoma |
| ILMN_2668236 | 100503659 | Dos | 1.194 | 8.545 | 6.15E-11 | adenocarcinoma |
| ILMN_2594066 | 66532 | Rep15 | -1.989 | 14.186 | 6.36E-11 | adenocarcinoma |
| ILMN_2634389 | 26424 | Nr5a2 | -1.673 | 9.584 | 6.36E-11 | adenocarcinoma |
| ILMN_1233064 | 12566 | Cdk2 | 1.641 | 10.258 | 6.41E-11 | adenocarcinoma |
| ILMN_3140788 | 19205 | Ptbp1 | 1.344 | 11.577 | 6.67E-11 | adenocarcinoma |
| ILMN_2846194 | 52430 | Echdc2 | 2.400 | 10.168 | 6.67E-11 | adenocarcinoma |
| ILMN_2890866 | 66813 | Bcl2l14 | -1.292 | 9.774 | 6.89E-11 | adenocarcinoma |
| ILMN_2818484 | 493583 | Itlnb | 3.535 | 9.195 | 6.93E-11 | adenocarcinoma |
| ILMN_1250435 | 66938 | Sh3d21 | -1.757 | 13.677 | 6.97E-11 | adenocarcinoma |
| ILMN_2970167 | 66894 | Wwp2 | -1.032 | 9.382 | 7.05E-11 | adenocarcinoma |
| ILMN_1225226 | 14450 | Gart | 1.218 | 9.879 | 7.08E-11 | adenocarcinoma |
| ILMN_2524817 | 69926 | Dnah17 | 1.339 | 8.551 | 7.15E-11 | adenocarcinoma |
| ILMN_2691493 | 56390 | Sssca1 | 1.173 | 10.054 | 7.15E-11 | adenocarcinoma |
| ILMN_1252602 | 56398 | Chp1 | -1.230 | 9.153 | 7.21E-11 | adenocarcinoma |
| ILMN_2688287 | 70835 | Prss22 | 2.135 | 8.326 | 7.21E-11 | adenocarcinoma |
| ILMN_2487482 | 22751 | Zfp90 | 1.278 | 9.192 | 7.21E-11 | adenocarcinoma |
| ILMN_2571683 | 19205 | Ptbp1 | 1.257 | 10.040 | 7.53E-11 | adenocarcinoma |
| ILMN_2799361 | 57783 | Tnip1 | -1.528 | 10.073 | 7.57E-11 | adenocarcinoma |
| ILMN_2746830 | 192970 | Dhrs11 | -2.054 | 10.256 | 7.69E-11 | adenocarcinoma |
| ILMN_1234738 | 106757 | Catsperd | 1.724 | 8.455 | 7.87E-11 | adenocarcinoma |
| ILMN_2604310 | 69123 | Eci3 | -1.171 | 9.140 | 7.88E-11 | adenocarcinoma |
| ILMN_2693913 | 225631 | Onecut2 | 2.775 | 8.657 | 7.91E-11 | adenocarcinoma |
| ILMN_1224116 | 57319 | Smpdl3a | -1.674 | 12.195 | 8.02E-11 | adenocarcinoma |
| ILMN_2559248 | NA | NA | -1.891 | 9.094 | 8.05E-11 | adenocarcinoma |
| ILMN_2726128 | 15203 | Heph | -1.420 | 10.276 | 8.07E-11 | adenocarcinoma |
| ILMN_1246145 | 270328 | Gsdmc3 | -2.791 | 10.686 | 8.10E-11 | adenocarcinoma |
| ILMN_2661125 | 94275 | Maged1 | 1.730 | 11.857 | 8.26E-11 | adenocarcinoma |
| ILMN_2660754 | NA | NA | 1.059 | 8.178 | 8.39E-11 | adenocarcinoma |
| ILMN_1233831 | 71601 | Ceacam20 | -1.879 | 11.896 | 8.52E-11 | adenocarcinoma |
| ILMN_1225520 | 66356 | Knop1 | 1.116 | 9.739 | 8.52E-11 | adenocarcinoma |
| ILMN_2803674 | 20202 | S100a9 | 2.287 | 8.340 | 8.52E-11 | adenocarcinoma |
| ILMN_2539337 | 68910 | Zfp467 | -1.697 | 9.529 | 8.55E-11 | adenocarcinoma |
| ILMN_1237140 | 101943 | Sf3b3 | 1.268 | 10.641 | 8.55E-11 | adenocarcinoma |
| ILMN_2994380 | 17961 | Nat2 | -1.492 | 10.576 | 8.81E-11 | adenocarcinoma |
| ILMN_2772319 | 12859 | Cox5b | -1.065 | 13.078 | 8.82E-11 | adenocarcinoma |
| ILMN_1254622 | 66425 | Pcp4l1 | -1.341 | 9.274 | 8.85E-11 | adenocarcinoma |
| ILMN_1240274 | 227723 | Prrc2b | 1.454 | 11.085 | 8.93E-11 | adenocarcinoma |
| ILMN_2747456 | 117198 | Ivns1abp | -1.259 | 12.353 | 8.93E-11 | adenocarcinoma |
| ILMN_2622163 | 14450 | Gart | 1.155 | 8.951 | 8.93E-11 | adenocarcinoma |
| ILMN_1225873 | 20510 | Slc1a1 | -1.686 | 9.625 | 8.93E-11 | adenocarcinoma |
| ILMN_2773936 | 14739 | S1pr2 | 1.410 | 9.641 | 8.93E-11 | adenocarcinoma |
| ILMN_2727013 | 20186 | Nr1h4 | -2.229 | 11.916 | 8.93E-11 | adenocarcinoma |
| ILMN_1259546 | 217212 | Pyy | -2.168 | 13.054 | 9.01E-11 | adenocarcinoma |
| ILMN_2960714 | 68262 | Agpat4 | -1.651 | 11.777 | 9.51E-11 | adenocarcinoma |
| ILMN_2592486 | 21946 | Pglyrp1 | 2.326 | 12.582 | 9.61E-11 | adenocarcinoma |
| ILMN_2485340 | 269181 | Mgat4a | -1.231 | 13.892 | 9.71E-11 | adenocarcinoma |
| ILMN_2646640 | 67080 | 1700019D03Rik | -2.226 | 11.376 | 9.84E-11 | adenocarcinoma |
| ILMN_2735294 | 68312 | Gstm7 | 1.386 | 8.670 | 9.99E-11 | adenocarcinoma |
| ILMN_3034303 | 268663 | Cdhr2 | -1.460 | 12.576 | 1.00E-10 | adenocarcinoma |
| ILMN_2687547 | 20324 | Sdpr | -1.736 | 10.114 | 1.00E-10 | adenocarcinoma |
| ILMN_2847834 | 22248 | Unc119 | -1.203 | 9.418 | 1.01E-10 | adenocarcinoma |
| ILMN_1251416 | 20404 | Sh3gl2 | -1.559 | 10.776 | 1.01E-10 | adenocarcinoma |
| ILMN_2737192 | 53356 | Eif3g | 1.291 | 12.857 | 1.01E-10 | adenocarcinoma |
| ILMN_2959293 | 22271 | Upp1 | -2.310 | 10.514 | 1.02E-10 | adenocarcinoma |
| ILMN_2841334 | 235469 | Zfp280d | 1.231 | 9.349 | 1.02E-10 | adenocarcinoma |
| ILMN_1229516 | 15484 | Hsd11b2 | -1.749 | 10.890 | 1.02E-10 | adenocarcinoma |
| ILMN_3133448 | 17304 | Mfge8 | 2.580 | 12.746 | 1.02E-10 | adenocarcinoma |
| ILMN_1254631 | NA | NA | 1.791 | 11.493 | 1.02E-10 | adenocarcinoma |
| ILMN_2727980 | 94184 | Pdxdc1 | -1.225 | 11.251 | 1.03E-10 | adenocarcinoma |
| ILMN_1256019 | 11364 | Acadm | -1.653 | 11.171 | 1.03E-10 | adenocarcinoma |
| ILMN_2731769 | 226971 | Plekhb2 | -1.119 | 12.942 | 1.04E-10 | adenocarcinoma |
| ILMN_2827080 | 27219 | Sgk2 | -1.718 | 9.475 | 1.06E-10 | adenocarcinoma |
| ILMN_2493826 | NA | NA | -2.253 | 13.276 | 1.06E-10 | adenocarcinoma |
| ILMN_1222112 | 383491 | Prdm14 | -1.140 | 8.871 | 1.07E-10 | adenocarcinoma |
| ILMN_3160626 | 12684 | Cideb | -1.747 | 11.314 | 1.08E-10 | adenocarcinoma |
| ILMN_1241523 | NA | NA | 1.414 | 8.324 | 1.12E-10 | adenocarcinoma |
| ILMN_2769832 | 81879 | Tfcp2l1 | -1.989 | 12.961 | 1.15E-10 | adenocarcinoma |
| ILMN_2634933 | 57890 | Il17re | -1.191 | 9.907 | 1.15E-10 | adenocarcinoma |
| ILMN_3052781 | 384071 | Slc25a34 | -1.410 | 9.345 | 1.16E-10 | adenocarcinoma |
| ILMN_2920309 | 66966 | Trit1 | 1.202 | 10.063 | 1.19E-10 | adenocarcinoma |
| ILMN_2600627 | 100608 | Noc4l | 1.355 | 8.953 | 1.21E-10 | adenocarcinoma |
| ILMN_1250928 | 11906 | Zfhx3 | 1.818 | 10.672 | 1.21E-10 | adenocarcinoma |
| ILMN_2607408 | 59092 | Pcbp4 | 1.961 | 10.013 | 1.21E-10 | adenocarcinoma |
| ILMN_2650008 | 14160 | Lgr5 | 1.317 | 8.265 | 1.21E-10 | adenocarcinoma |
| ILMN_1252444 | 99526 | Usp53 | -1.450 | 10.226 | 1.21E-10 | adenocarcinoma |
| ILMN_2713714 | 15567 | Slc6a4 | -1.829 | 9.455 | 1.26E-10 | adenocarcinoma |
| ILMN_2659426 | 57266 | Cxcl14 | 1.781 | 8.352 | 1.30E-10 | adenocarcinoma |
| ILMN_2824002 | 212398 | Frat2 | -1.653 | 10.436 | 1.30E-10 | adenocarcinoma |
| ILMN_3060635 | 225997 | Trpm6 | -1.212 | 9.091 | 1.33E-10 | adenocarcinoma |
| ILMN_2594714 | 23872 | Ets2 | 1.414 | 13.066 | 1.38E-10 | adenocarcinoma |
| ILMN_2797726 | 74051 | Steap2 | 1.129 | 9.219 | 1.39E-10 | adenocarcinoma |
| ILMN_2904641 | NA | NA | 1.400 | 9.134 | 1.39E-10 | adenocarcinoma |
| ILMN_1221348 | 394432 | Ugt1a7c | -1.559 | 9.245 | 1.40E-10 | adenocarcinoma |
| ILMN_2769064 | 67680 | Sdhb | -1.257 | 11.183 | 1.40E-10 | adenocarcinoma |
| ILMN_1258601 | 22628 | Ywhag | 1.288 | 9.994 | 1.47E-10 | adenocarcinoma |
| ILMN_2883990 | 56878 | Rbms1 | 1.408 | 10.705 | 1.49E-10 | adenocarcinoma |
| ILMN_2690396 | 67169 | Nradd | 1.278 | 8.435 | 1.51E-10 | adenocarcinoma |
| ILMN_2592834 | 20287 | Sct | -1.345 | 10.717 | 1.53E-10 | adenocarcinoma |
| ILMN_2836982 | 13346 | Des | -3.163 | 11.604 | 1.53E-10 | adenocarcinoma |
| ILMN_2678200 | 353172 | Gars | 1.384 | 13.883 | 1.55E-10 | adenocarcinoma |
| ILMN_2695793 | 14159 | Fes | 1.276 | 9.033 | 1.56E-10 | adenocarcinoma |
| ILMN_2681583 | 74552 | Nipal3 | -1.277 | 10.563 | 1.56E-10 | adenocarcinoma |
| ILMN_1253015 | 72112 | Ppp1r14d | -1.645 | 11.352 | 1.56E-10 | adenocarcinoma |
| ILMN_2534790 | 76273 | Ndfip2 | -1.670 | 10.620 | 1.56E-10 | adenocarcinoma |
| ILMN_2644936 | 226243 | Habp2 | 1.599 | 8.334 | 1.57E-10 | adenocarcinoma |
| ILMN_2731265 | 15484 | Hsd11b2 | -1.913 | 10.678 | 1.59E-10 | adenocarcinoma |
| ILMN_1233836 | 330260 | Pon2 | 1.087 | 13.281 | 1.67E-10 | adenocarcinoma |
| ILMN_1232298 | 192173 | Fam195b | 1.472 | 11.144 | 1.67E-10 | adenocarcinoma |
| ILMN_2797642 | 215387 | Ncaph | 1.648 | 8.756 | 1.69E-10 | adenocarcinoma |
| ILMN_2746906 | 110959 | Nudt19 | -1.282 | 12.948 | 1.69E-10 | adenocarcinoma |
| ILMN_2806700 | 13057 | Cyba | 1.528 | 11.948 | 1.69E-10 | adenocarcinoma |
| ILMN_2774507 | 210622 | Pamr1 | -1.979 | 9.807 | 1.72E-10 | adenocarcinoma |
| ILMN_2701355 | 332397 | Nanos1 | 1.406 | 8.296 | 1.73E-10 | adenocarcinoma |
| ILMN_1239221 | 69573 | Hilpda | 1.147 | 9.271 | 1.76E-10 | adenocarcinoma |
| ILMN_1258988 | 16010 | Igfbp4 | 2.605 | 11.128 | 1.76E-10 | adenocarcinoma |
| ILMN_2777471 | 15040 | H2-T23 | -1.870 | 13.001 | 1.77E-10 | adenocarcinoma |
| ILMN_2721360 | 14635 | Galk1 | 1.236 | 9.337 | 1.84E-10 | adenocarcinoma |
| ILMN_1242013 | 80914 | Uck2 | 1.309 | 9.635 | 1.84E-10 | adenocarcinoma |
| ILMN_1241270 | 208518 | Cep78 | 1.765 | 9.852 | 1.85E-10 | adenocarcinoma |
| ILMN_2773113 | 12332 | Capg | 2.031 | 10.204 | 1.85E-10 | adenocarcinoma |
| ILMN_1236517 | 16173 | Il18 | -2.246 | 11.404 | 1.86E-10 | adenocarcinoma |
| ILMN_2763245 | 14825 | Cxcl1 | 3.660 | 9.147 | 1.86E-10 | adenocarcinoma |
| ILMN_2764995 | 70572 | Ipo5 | 1.179 | 10.010 | 1.87E-10 | adenocarcinoma |
| ILMN_2734646 | 20866 | Stim1 | -1.536 | 10.441 | 1.88E-10 | adenocarcinoma |
| ILMN_2943599 | 12070 | Ngfrap1 | 1.707 | 10.700 | 1.90E-10 | adenocarcinoma |
| ILMN_2942674 | 225341 | Lims2 | -1.347 | 9.252 | 1.93E-10 | adenocarcinoma |
| ILMN_2740151 | 212862 | Chpt1 | -1.474 | 9.904 | 1.95E-10 | adenocarcinoma |
| ILMN_2651706 | 207521 | Dtx4 | 1.036 | 8.689 | 1.96E-10 | adenocarcinoma |
| ILMN_2685157 | 76408 | Abcc3 | -1.416 | 10.923 | 1.99E-10 | adenocarcinoma |
| ILMN_1231293 | 77996 | Cutal | -1.902 | 10.573 | 2.00E-10 | adenocarcinoma |
| ILMN_1251165 | 71041 | Pcgf6 | 1.483 | 9.478 | 2.00E-10 | adenocarcinoma |
| ILMN_2793739 | 381236 | Lipo1 | -1.226 | 9.404 | 2.03E-10 | adenocarcinoma |
| ILMN_1231724 | 19711 | Resp18 | -1.454 | 8.950 | 2.03E-10 | adenocarcinoma |
| ILMN_2923865 | 12368 | Casp6 | 1.220 | 10.999 | 2.04E-10 | adenocarcinoma |
| ILMN_1233187 | 17187 | Max | -1.300 | 13.169 | 2.04E-10 | adenocarcinoma |
| ILMN_2750801 | 68275 | Rpa1 | 1.538 | 11.982 | 2.05E-10 | adenocarcinoma |
| ILMN_1250907 | 17216 | Mcm2 | 1.746 | 9.101 | 2.05E-10 | adenocarcinoma |
| ILMN_2911551 | 14917 | Gucy2c | -1.339 | 9.292 | 2.05E-10 | adenocarcinoma |
| ILMN_2491654 | 68926 | Ubap2 | 1.599 | 11.496 | 2.06E-10 | adenocarcinoma |
| ILMN_2934532 | 28000 | Prpf19 | 1.453 | 11.842 | 2.11E-10 | adenocarcinoma |
| ILMN_1252601 | 77045 | Bcl7a | 1.478 | 9.118 | 2.13E-10 | adenocarcinoma |
| ILMN_3152241 | 104681 | Slc16a6 | 1.103 | 8.283 | 2.20E-10 | adenocarcinoma |
| ILMN_2918499 | 18669 | Abcb1b | 1.194 | 8.344 | 2.20E-10 | adenocarcinoma |
| ILMN_1233175 | 18938 | Ppp1r14b | 1.416 | 11.541 | 2.20E-10 | adenocarcinoma |
| ILMN_2602185 | 53860 | Sep-09 | 1.090 | 8.914 | 2.20E-10 | adenocarcinoma |
| ILMN_2632839 | 71774 | Shroom1 | 1.403 | 8.255 | 2.25E-10 | adenocarcinoma |
| ILMN_2642462 | 14886 | Gtf2i | 1.501 | 12.738 | 2.26E-10 | adenocarcinoma |
| ILMN_2473692 | NA | NA | 1.472 | 8.522 | 2.27E-10 | adenocarcinoma |
| ILMN_2798138 | 54120 | Gipc2 | -1.475 | 12.121 | 2.28E-10 | adenocarcinoma |
| ILMN_2728754 | 57320 | Park7 | 1.150 | 13.252 | 2.29E-10 | adenocarcinoma |
| ILMN_2619639 | 233066 | Syne4 | 1.716 | 8.330 | 2.32E-10 | adenocarcinoma |
| ILMN_1233523 | NA | NA | -1.202 | 8.944 | 2.32E-10 | adenocarcinoma |
| ILMN_1221985 | 73610 | Zfp433 | -1.077 | 8.311 | 2.34E-10 | adenocarcinoma |
| ILMN_2784950 | 18807 | Pld3 | 1.044 | 9.022 | 2.34E-10 | adenocarcinoma |
| ILMN_2717613 | 12566 | Cdk2 | 1.193 | 9.146 | 2.35E-10 | adenocarcinoma |
| ILMN_1236796 | 20595 | Smn1 | 1.296 | 11.275 | 2.36E-10 | adenocarcinoma |
| ILMN_1221264 | 16600 | Klf4 | -1.972 | 10.946 | 2.40E-10 | adenocarcinoma |
| ILMN_1240257 | 108148 | Galnt2 | 1.052 | 11.213 | 2.42E-10 | adenocarcinoma |
| ILMN_3074610 | 545288 | Cyp2c67 | -1.846 | 9.707 | 2.46E-10 | adenocarcinoma |
| ILMN_2505100 | 71609 | Tradd | -1.431 | 10.542 | 2.46E-10 | adenocarcinoma |
| ILMN_2693314 | 13113 | Cyp3a13 | -1.306 | 9.013 | 2.47E-10 | adenocarcinoma |
| ILMN_2739872 | 50916 | Irx4 | 1.514 | 8.164 | 2.47E-10 | adenocarcinoma |
| ILMN_2901029 | 328977 | Zfp532 | 1.088 | 9.042 | 2.48E-10 | adenocarcinoma |
| ILMN_1250860 | 239673 | 4732456N10Rik | 1.791 | 8.322 | 2.49E-10 | adenocarcinoma |
| ILMN_2647331 | 73181 | Nfatc4 | 1.527 | 9.074 | 2.51E-10 | adenocarcinoma |
| ILMN_2448997 | 240427 | Setbp1 | -1.189 | 9.461 | 2.53E-10 | adenocarcinoma |
| ILMN_2691492 | 56390 | Sssca1 | 1.097 | 9.642 | 2.55E-10 | adenocarcinoma |
| ILMN_2872371 | 72112 | Ppp1r14d | -1.903 | 12.213 | 2.55E-10 | adenocarcinoma |
| ILMN_2668696 | 16164 | Il13ra1 | -1.218 | 11.614 | 2.57E-10 | adenocarcinoma |
| ILMN_1240471 | 67442 | Retsat | -1.936 | 12.895 | 2.59E-10 | adenocarcinoma |
| ILMN_1222543 | NA | NA | -2.219 | 13.161 | 2.59E-10 | adenocarcinoma |
| ILMN_1233376 | 106522 | Pkdcc | -1.135 | 9.125 | 2.59E-10 | adenocarcinoma |
| ILMN_2993109 | 74747 | Ddit4 | 1.638 | 11.358 | 2.59E-10 | adenocarcinoma |
| ILMN_2583829 | NA | NA | 1.045 | 8.029 | 2.59E-10 | adenocarcinoma |
| ILMN_2645737 | 18171 | Nr1i2 | -1.382 | 9.717 | 2.59E-10 | adenocarcinoma |
| ILMN_2735118 | 226169 | Pprc1 | 1.609 | 9.194 | 2.61E-10 | adenocarcinoma |
| ILMN_2436781 | 74356 | 4931428F04Rik | 1.051 | 8.726 | 2.65E-10 | adenocarcinoma |
| ILMN_2635197 | 76943 | Psapl1 | 2.375 | 8.643 | 2.66E-10 | adenocarcinoma |
| ILMN_2825574 | 73710 | Tubb2b | 1.015 | 8.230 | 2.68E-10 | adenocarcinoma |
| ILMN_1246201 | 58226 | Cacna1h | 1.699 | 9.475 | 2.72E-10 | adenocarcinoma |
| ILMN_3126609 | 26365 | Ceacam1 | -2.058 | 14.884 | 2.74E-10 | adenocarcinoma |
| ILMN_1244261 | 20216 | Acsm3 | -2.224 | 11.123 | 2.74E-10 | adenocarcinoma |
| ILMN_2733277 | 192287 | Slc25a36 | -1.143 | 9.935 | 2.75E-10 | adenocarcinoma |
| ILMN_1236906 | 52626 | Cdkn2aipnl | 1.125 | 10.765 | 2.75E-10 | adenocarcinoma |
| ILMN_3000080 | 68778 | Gucd1 | -1.596 | 12.694 | 2.77E-10 | adenocarcinoma |
| ILMN_3160190 | 381122 | Capn13 | -2.288 | 11.403 | 2.77E-10 | adenocarcinoma |
| ILMN_1230119 | 12070 | Ngfrap1 | 1.622 | 10.464 | 2.77E-10 | adenocarcinoma |
| ILMN_1257666 | 52588 | Tspan14 | 1.178 | 10.866 | 2.77E-10 | adenocarcinoma |
| ILMN_2708906 | 18010 | Neu1 | -1.213 | 13.436 | 2.78E-10 | adenocarcinoma |
| ILMN_2840975 | 216456 | Gls2 | 1.752 | 8.426 | 2.81E-10 | adenocarcinoma |
| ILMN_3161263 | 14367 | Fzd5 | -1.529 | 11.993 | 2.83E-10 | adenocarcinoma |
| ILMN_2660862 | 74777 | Sepn1 | 1.739 | 9.466 | 2.89E-10 | adenocarcinoma |
| ILMN_2746556 | 50781 | Dkk3 | 2.268 | 10.338 | 2.90E-10 | adenocarcinoma |
| ILMN_1240598 | 217125 | Samd14 | 1.185 | 9.371 | 2.92E-10 | adenocarcinoma |
| ILMN_2768026 | 110006 | Gusb | 1.188 | 8.810 | 2.97E-10 | adenocarcinoma |
| ILMN_2766875 | 433904 | Ociad2 | -1.704 | 13.197 | 3.01E-10 | adenocarcinoma |
| ILMN_2836586 | 68294 | Mfsd10 | 1.293 | 8.639 | 3.01E-10 | adenocarcinoma |
| ILMN_2432550 | 217410 | Trib2 | 1.258 | 8.570 | 3.01E-10 | adenocarcinoma |
| ILMN_2916035 | 16906 | Lmnb1 | 1.812 | 9.607 | 3.02E-10 | adenocarcinoma |
| ILMN_2757641 | 13640 | Efna5 | -1.129 | 9.192 | 3.09E-10 | adenocarcinoma |
| ILMN_2628567 | 27280 | Phlda3 | 1.572 | 8.589 | 3.10E-10 | adenocarcinoma |
| ILMN_3027751 | 20411 | Sorbs1 | -1.147 | 9.156 | 3.10E-10 | adenocarcinoma |
| ILMN_1252400 | 76630 | Stambpl1 | -1.094 | 9.409 | 3.16E-10 | adenocarcinoma |
| ILMN_1256563 | 234407 | Glt25d1 | 1.484 | 9.495 | 3.18E-10 | adenocarcinoma |
| ILMN_2977535 | 229473 | D930015E06Rik | 1.626 | 8.793 | 3.20E-10 | adenocarcinoma |
| ILMN_2986393 | 22143 | Tuba1b | 1.466 | 13.471 | 3.21E-10 | adenocarcinoma |
| ILMN_2620122 | 18438 | P2rx4 | -1.430 | 10.391 | 3.24E-10 | adenocarcinoma |
| ILMN_2445324 | 22761 | Zfpm1 | 2.389 | 9.804 | 3.28E-10 | adenocarcinoma |
| ILMN_1230443 | 22003 | Tpm1 | -2.442 | 10.477 | 3.31E-10 | adenocarcinoma |
| ILMN_2734391 | 51801 | Ramp1 | -1.322 | 11.337 | 3.34E-10 | adenocarcinoma |
| ILMN_2773259 | 17022 | Lum | -1.363 | 8.672 | 3.42E-10 | adenocarcinoma |
| ILMN_2513870 | 22637 | Zap70 | 1.514 | 8.599 | 3.46E-10 | adenocarcinoma |
| ILMN_2899788 | 19732 | Rgl2 | 1.587 | 10.232 | 3.46E-10 | adenocarcinoma |
| ILMN_2727893 | 67444 | Ilkap | 1.143 | 11.459 | 3.47E-10 | adenocarcinoma |
| ILMN_2613558 | 71949 | Cers5 | -1.242 | 12.189 | 3.54E-10 | adenocarcinoma |
| ILMN_1213786 | 269702 | Mphosph9 | 1.283 | 9.255 | 3.56E-10 | adenocarcinoma |
| ILMN_2789294 | 71687 | Tmem25 | -1.138 | 9.372 | 3.58E-10 | adenocarcinoma |
| ILMN_2672969 | 270328 | Gsdmc3 | -2.331 | 10.033 | 3.58E-10 | adenocarcinoma |
| ILMN_2676481 | 56209 | Gde1 | -1.292 | 11.373 | 3.60E-10 | adenocarcinoma |
| ILMN_2598852 | 19385 | Ranbp1 | 1.324 | 12.133 | 3.60E-10 | adenocarcinoma |
| ILMN_2822141 | 67636 | Lyrm5 | -1.613 | 10.681 | 3.66E-10 | adenocarcinoma |
| ILMN_2870864 | 210622 | Pamr1 | -1.729 | 9.634 | 3.67E-10 | adenocarcinoma |
| ILMN_2661366 | 235135 | Tmem45b | -1.394 | 15.094 | 3.69E-10 | adenocarcinoma |
| ILMN_1258534 | 11972 | Atp6v0d1 | -1.107 | 10.733 | 3.69E-10 | adenocarcinoma |
| ILMN_1218866 | 20362 | Sep-08 | 1.285 | 9.398 | 3.70E-10 | adenocarcinoma |
| ILMN_2724322 | 21771 | Cirh1a | 1.066 | 10.493 | 3.74E-10 | adenocarcinoma |
| ILMN_1215647 | 68114 | Mum1 | 1.273 | 11.198 | 3.85E-10 | adenocarcinoma |
| ILMN_2671259 | 27979 | Eif3b | 1.309 | 10.376 | 3.87E-10 | adenocarcinoma |
| ILMN_2419078 | 75957 | Mir17hg | 1.339 | 9.391 | 3.87E-10 | adenocarcinoma |
| ILMN_2526987 | 67160 | Eef1g | 1.033 | 12.223 | 3.96E-10 | adenocarcinoma |
| ILMN_3152380 | 14252 | Flot2 | 1.190 | 10.634 | 3.96E-10 | adenocarcinoma |
| ILMN_1250438 | 17357 | Marcksl1 | 1.670 | 8.827 | 3.96E-10 | adenocarcinoma |
| ILMN_1239181 | 67824 | Nmral1 | 1.203 | 8.832 | 3.96E-10 | adenocarcinoma |
| ILMN_3103904 | 19266 | Ptprd | -1.425 | 10.365 | 3.96E-10 | adenocarcinoma |
| ILMN_1224606 | 13521 | Slc26a2 | -1.929 | 9.301 | 4.06E-10 | adenocarcinoma |
| ILMN_3084087 | 117198 | Ivns1abp | -1.404 | 11.552 | 4.06E-10 | adenocarcinoma |
| ILMN_3076534 | NA | NA | -1.854 | 10.745 | 4.12E-10 | adenocarcinoma |
| ILMN_1217928 | 19205 | Ptbp1 | 1.222 | 9.523 | 4.15E-10 | adenocarcinoma |
| ILMN_2491589 | 232334 | Vgll4 | 1.373 | 9.160 | 4.19E-10 | adenocarcinoma |
| ILMN_1260397 | 100036521 | Gm16039 | -1.527 | 10.350 | 4.25E-10 | adenocarcinoma |
| ILMN_1257579 | 54563 | Nup210 | 1.995 | 10.797 | 4.25E-10 | adenocarcinoma |
| ILMN_2928160 | 22321 | Vars | 1.826 | 9.294 | 4.28E-10 | adenocarcinoma |
| ILMN_2624938 | 18611 | Pea15a | 1.412 | 10.040 | 4.28E-10 | adenocarcinoma |
| ILMN_1236899 | NA | NA | 1.328 | 8.692 | 4.31E-10 | adenocarcinoma |
| ILMN_2756891 | 99010 | Lpcat4 | 1.557 | 8.456 | 4.38E-10 | adenocarcinoma |
| ILMN_1259528 | NA | NA | 1.682 | 12.229 | 4.39E-10 | adenocarcinoma |
| ILMN_2959291 | 22271 | Upp1 | -2.587 | 12.327 | 4.39E-10 | adenocarcinoma |
| ILMN_2588650 | 269336 | Ccdc32 | -1.142 | 10.451 | 4.39E-10 | adenocarcinoma |
| ILMN_2599251 | 114654 | Ly6g6d | -1.161 | 9.418 | 4.42E-10 | adenocarcinoma |
| ILMN_2772117 | 16429 | Itln1 | 1.581 | 8.296 | 4.42E-10 | adenocarcinoma |
| ILMN_1246073 | 13590 | Lefty1 | -1.201 | 8.888 | 4.51E-10 | adenocarcinoma |
| ILMN_1233113 | 226352 | Epb4.1l5 | 1.017 | 9.058 | 4.51E-10 | adenocarcinoma |
| ILMN_1244458 | 238463 | Tubal3 | -1.576 | 9.708 | 4.52E-10 | adenocarcinoma |
| ILMN_2991019 | 393082 | Mettl7a2 | -3.111 | 9.626 | 4.52E-10 | adenocarcinoma |
| ILMN_2485410 | 69812 | Abhd11os | -1.367 | 9.952 | 4.52E-10 | adenocarcinoma |
| ILMN_2940446 | 13139 | Dgka | -1.367 | 11.320 | 4.55E-10 | adenocarcinoma |
| ILMN_2943387 | 22353 | Vip | -1.540 | 9.324 | 4.57E-10 | adenocarcinoma |
| ILMN_2608789 | 23954 | Nek3 | -1.087 | 9.433 | 4.57E-10 | adenocarcinoma |
| ILMN_2744587 | 56506 | Cib2 | 2.163 | 9.680 | 4.60E-10 | adenocarcinoma |
| ILMN_2642985 | 114664 | Hsd17b11 | -1.103 | 9.550 | 4.66E-10 | adenocarcinoma |
| ILMN_1256890 | 70122 | Mllt3 | -1.364 | 9.363 | 4.73E-10 | adenocarcinoma |
| ILMN_2965903 | 15186 | Hdc | 2.188 | 9.027 | 4.74E-10 | adenocarcinoma |
| ILMN_2700107 | 20019 | Polr1a | 1.843 | 10.438 | 4.82E-10 | adenocarcinoma |
| ILMN_2725188 | 11982 | Atp10a | 2.033 | 8.676 | 4.85E-10 | adenocarcinoma |
| ILMN_1245431 | 331046 | Tgm4 | 3.771 | 8.878 | 4.85E-10 | adenocarcinoma |
| ILMN_1218347 | 107589 | Mylk | -1.566 | 13.509 | 4.86E-10 | adenocarcinoma |
| ILMN_1214052 | 78449 | 2700046A07Rik | 1.508 | 8.198 | 4.97E-10 | adenocarcinoma |
| ILMN_2746766 | 226849 | Ppp2r5a | -1.011 | 11.514 | 5.03E-10 | adenocarcinoma |
| ILMN_2501929 | 19266 | Ptprd | -1.457 | 10.281 | 5.12E-10 | adenocarcinoma |
| ILMN_2752146 | 268860 | Abat | -1.016 | 8.564 | 5.13E-10 | adenocarcinoma |
| ILMN_2690873 | 19826 | Rnps1 | 1.002 | 10.076 | 5.16E-10 | adenocarcinoma |
| ILMN_2445378 | 72077 | Gcnt3 | -1.668 | 12.163 | 5.20E-10 | adenocarcinoma |
| ILMN_1248258 | NA | NA | -1.574 | 11.009 | 5.30E-10 | adenocarcinoma |
| ILMN_2828427 | 16897 | Llgl1 | 1.244 | 11.643 | 5.35E-10 | adenocarcinoma |
| ILMN_1258028 | 53897 | Gal3st1 | -1.318 | 11.394 | 5.38E-10 | adenocarcinoma |
| ILMN_1217927 | 60507 | Qtrt1 | 1.077 | 9.075 | 5.40E-10 | adenocarcinoma |
| ILMN_1231204 | 270152 | Amica1 | 1.958 | 9.251 | 5.40E-10 | adenocarcinoma |
| ILMN_1225835 | 50530 | Mfap5 | -1.265 | 8.467 | 5.48E-10 | adenocarcinoma |
| ILMN_2636335 | 20382 | Srsf2 | 1.183 | 12.874 | 5.49E-10 | adenocarcinoma |
| ILMN_2487170 | 22004 | Tpm2 | -2.654 | 11.286 | 5.51E-10 | adenocarcinoma |
| ILMN_2859613 | 18295 | Ogn | -2.799 | 10.894 | 5.59E-10 | adenocarcinoma |
| ILMN_2718645 | 269593 | Luzp1 | -1.196 | 11.678 | 5.63E-10 | adenocarcinoma |
| ILMN_2637094 | 18553 | Pcsk6 | 1.580 | 9.536 | 5.67E-10 | adenocarcinoma |
| ILMN_2540489 | 18938 | Ppp1r14b | 1.439 | 10.212 | 5.67E-10 | adenocarcinoma |
| ILMN_2870074 | 13848 | Ephb6 | 1.370 | 8.204 | 5.67E-10 | adenocarcinoma |
| ILMN_2649091 | 18799 | Plcd1 | -1.267 | 9.611 | 5.67E-10 | adenocarcinoma |
| ILMN_2914010 | 13401 | Dmwd | 1.039 | 9.014 | 5.68E-10 | adenocarcinoma |
| ILMN_1233664 | 216971 | Fam222b | 1.033 | 10.342 | 5.68E-10 | adenocarcinoma |
| ILMN_1212704 | 242505 | Rasef | -1.486 | 9.944 | 5.71E-10 | adenocarcinoma |
| ILMN_2612973 | 69083 | Sult1c2 | -2.358 | 10.588 | 5.79E-10 | adenocarcinoma |
| ILMN_2893321 | 330050 | Fam185a | 1.099 | 10.133 | 5.82E-10 | adenocarcinoma |
| ILMN_2701515 | 233328 | Lrrk1 | 1.325 | 10.366 | 5.84E-10 | adenocarcinoma |
| ILMN_2725781 | 27409 | Abcg5 | -2.625 | 11.491 | 5.88E-10 | adenocarcinoma |
| ILMN_2690976 | 12567 | Cdk4 | 1.598 | 10.218 | 5.88E-10 | adenocarcinoma |
| ILMN_3163001 | 97998 | Deptor | -1.268 | 10.390 | 5.92E-10 | adenocarcinoma |
| ILMN_1260241 | 218613 | Mier3 | -1.543 | 11.115 | 5.96E-10 | adenocarcinoma |
| ILMN_2777282 | 67160 | Eef1g | 1.235 | 14.275 | 6.00E-10 | adenocarcinoma |
| ILMN_2619983 | 15529 | Sdc2 | -1.191 | 9.800 | 6.25E-10 | adenocarcinoma |
| ILMN_1227723 | 68323 | Nudt22 | 1.267 | 10.241 | 6.41E-10 | adenocarcinoma |
| ILMN_2746783 | 338521 | Fa2h | -1.929 | 11.357 | 6.50E-10 | adenocarcinoma |
| ILMN_1225880 | 20810 | Srm | 1.228 | 9.241 | 6.51E-10 | adenocarcinoma |
| ILMN_1238423 | 56070 | Tcerg1 | 1.181 | 10.315 | 6.51E-10 | adenocarcinoma |
| ILMN_2527129 | 100504173 | Gm15466 | 1.286 | 10.176 | 6.61E-10 | adenocarcinoma |
| ILMN_3005740 | 22637 | Zap70 | 1.260 | 8.166 | 6.62E-10 | adenocarcinoma |
| ILMN_2628647 | 13195 | Ddc | -1.524 | 11.460 | 6.68E-10 | adenocarcinoma |
| ILMN_1242456 | 107351 | Kank1 | 1.149 | 9.913 | 6.75E-10 | adenocarcinoma |
| ILMN_2687156 | 319625 | Galm | -1.460 | 10.508 | 6.77E-10 | adenocarcinoma |
| ILMN_2999670 | 225742 | St8sia5 | -2.377 | 9.065 | 6.77E-10 | adenocarcinoma |
| ILMN_1257630 | 68617 | Soga2 | 1.292 | 8.930 | 6.77E-10 | adenocarcinoma |
| ILMN_1241903 | 16600 | Klf4 | -2.073 | 11.740 | 6.79E-10 | adenocarcinoma |
| ILMN_2722996 | 19261 | Sirpa | 1.247 | 8.863 | 6.94E-10 | adenocarcinoma |
| ILMN_2521014 | 212989 | Best2 | -1.349 | 10.601 | 6.94E-10 | adenocarcinoma |
| ILMN_2699167 | 110842 | Etfa | -1.358 | 11.386 | 7.00E-10 | adenocarcinoma |
| ILMN_2710354 | 11475 | Acta2 | -2.459 | 11.247 | 7.02E-10 | adenocarcinoma |
| ILMN_2517566 | NA | NA | 1.651 | 8.151 | 7.04E-10 | adenocarcinoma |
| ILMN_1227209 | 68927 | Ptcd2 | -1.217 | 9.679 | 7.07E-10 | adenocarcinoma |
| ILMN_1228958 | 14313 | Fst | 1.127 | 8.254 | 7.15E-10 | adenocarcinoma |
| ILMN_2959976 | 21968 | Tom1 | -1.050 | 9.086 | 7.15E-10 | adenocarcinoma |
| ILMN_2702471 | 11534 | Adk | -1.410 | 11.825 | 7.20E-10 | adenocarcinoma |
| ILMN_1236757 | 67041 | Oxct1 | -1.754 | 12.900 | 7.23E-10 | adenocarcinoma |
| ILMN_2640883 | 67203 | Nde1 | 1.163 | 8.920 | 7.23E-10 | adenocarcinoma |
| ILMN_1217879 | 13877 | Erh | 1.384 | 11.356 | 7.37E-10 | adenocarcinoma |
| ILMN_2972855 | 78830 | Slc25a12 | -1.035 | 9.366 | 7.41E-10 | adenocarcinoma |
| ILMN_2670895 | 53817 | Ddx39b | 1.239 | 12.949 | 7.43E-10 | adenocarcinoma |
| ILMN_2698115 | 353258 | Ltv1 | 1.143 | 9.332 | 7.55E-10 | adenocarcinoma |
| ILMN_2698843 | 68087 | Dcakd | 1.256 | 11.363 | 7.75E-10 | adenocarcinoma |
| ILMN_2757092 | 66847 | Hint3 | -1.303 | 11.083 | 7.82E-10 | adenocarcinoma |
| ILMN_2621074 | 66071 | Ethe1 | -1.738 | 13.382 | 7.93E-10 | adenocarcinoma |
| ILMN_3160750 | 433470 | AA467197 | -1.631 | 14.550 | 7.95E-10 | adenocarcinoma |
| ILMN_2964185 | 14990 | H2-M2 | -1.662 | 9.179 | 7.95E-10 | adenocarcinoma |
| ILMN_1247553 | 20363 | Sepp1 | -1.866 | 9.634 | 7.95E-10 | adenocarcinoma |
| ILMN_2924419 | NA | NA | -1.211 | 9.355 | 8.04E-10 | adenocarcinoma |
| ILMN_2438516 | NA | NA | -1.085 | 9.733 | 8.07E-10 | adenocarcinoma |
| ILMN_2616309 | 108705 | Pttg1ip | -1.273 | 14.866 | 8.09E-10 | adenocarcinoma |
| ILMN_2790373 | 20621 | Snn | 1.831 | 9.821 | 8.13E-10 | adenocarcinoma |
| ILMN_3006911 | 170716 | Cyp4f13 | -1.480 | 11.699 | 8.18E-10 | adenocarcinoma |
| ILMN_1237586 | 69814 | Prss32 | -1.562 | 13.500 | 8.22E-10 | adenocarcinoma |
| ILMN_2615096 | 13482 | Dpp4 | -1.150 | 10.825 | 8.22E-10 | adenocarcinoma |
| ILMN_2622374 | 17984 | Ndn | -1.396 | 9.119 | 8.24E-10 | adenocarcinoma |
| ILMN_1260404 | 74356 | 4931428F04Rik | 1.027 | 8.632 | 8.24E-10 | adenocarcinoma |
| ILMN_2614351 | 68607 | Serhl | -1.275 | 10.334 | 8.30E-10 | adenocarcinoma |
| ILMN_2655015 | 17025 | Alad | 1.407 | 13.255 | 8.50E-10 | adenocarcinoma |
| ILMN_2753792 | 20897 | Stra6 | 2.809 | 8.616 | 8.66E-10 | adenocarcinoma |
| ILMN_1231062 | 195018 | Zzef1 | -1.083 | 9.411 | 8.66E-10 | adenocarcinoma |
| ILMN_2699923 | 67855 | Asprv1 | 2.546 | 8.713 | 8.66E-10 | adenocarcinoma |
| ILMN_1229315 | 21961 | Tns1 | -1.291 | 9.691 | 8.68E-10 | adenocarcinoma |
| ILMN_1215755 | 101612 | Grwd1 | 1.085 | 8.427 | 8.82E-10 | adenocarcinoma |
| ILMN_2648618 | 66251 | Arfgap3 | -1.010 | 11.216 | 8.82E-10 | adenocarcinoma |
| ILMN_2988849 | 228366 | Gyltl1b | 1.551 | 8.517 | 8.95E-10 | adenocarcinoma |
| ILMN_2444411 | 81877 | Tnxb | -1.123 | 9.135 | 9.07E-10 | adenocarcinoma |
| ILMN_2772380 | 69902 | Mrto4 | 1.052 | 9.098 | 9.15E-10 | adenocarcinoma |
| ILMN_2636666 | 19125 | Prodh | 1.675 | 9.248 | 9.20E-10 | adenocarcinoma |
| ILMN_3061070 | 57279 | Slc25a20 | -1.891 | 10.235 | 9.28E-10 | adenocarcinoma |
| ILMN_2741402 | 16651 | Sspn | -1.556 | 9.487 | 9.33E-10 | adenocarcinoma |
| ILMN_2868997 | 19387 | Rangap1 | 1.365 | 11.893 | 9.40E-10 | adenocarcinoma |
| ILMN_2826869 | 20208 | Saa1 | -2.618 | 11.156 | 9.48E-10 | adenocarcinoma |
| ILMN_1250837 | 19387 | Rangap1 | 1.451 | 12.059 | 9.62E-10 | adenocarcinoma |
| ILMN_2735308 | 72002 | Slc39a5 | -1.593 | 10.186 | 9.63E-10 | adenocarcinoma |
| ILMN_2774163 | 55944 | Eif3d | 1.113 | 13.271 | 9.70E-10 | adenocarcinoma |
| ILMN_2667352 | 109801 | Glo1 | 1.043 | 14.497 | 9.70E-10 | adenocarcinoma |
| ILMN_2957862 | 100608 | Noc4l | 1.447 | 9.694 | 9.99E-10 | adenocarcinoma |
| ILMN_2673233 | 56421 | Pfkp | -1.367 | 9.801 | 9.99E-10 | adenocarcinoma |
| ILMN_2847332 | 68041 | Mid1ip1 | 1.170 | 10.184 | 9.99E-10 | adenocarcinoma |
| ILMN_2772422 | 70337 | Iyd | -2.013 | 9.884 | 1.01E-09 | adenocarcinoma |
| ILMN_2777082 | 18452 | P4ha2 | 1.282 | 9.218 | 1.02E-09 | adenocarcinoma |
| ILMN_2757870 | 19171 | Psmb10 | -1.243 | 12.412 | 1.02E-09 | adenocarcinoma |
| ILMN_2777474 | 15040 | H2-T23 | -1.880 | 13.022 | 1.03E-09 | adenocarcinoma |
| ILMN_2610981 | 330962 | Slc51b | -2.334 | 10.067 | 1.04E-09 | adenocarcinoma |
| ILMN_2608043 | 55927 | Hes6 | 1.561 | 10.046 | 1.04E-09 | adenocarcinoma |
| ILMN_2686975 | 227737 | Fam129b | 1.193 | 11.861 | 1.04E-09 | adenocarcinoma |
| ILMN_1248381 | 108645 | Mat2b | -1.382 | 10.269 | 1.05E-09 | adenocarcinoma |
| ILMN_2565252 | 19205 | Ptbp1 | 1.047 | 9.098 | 1.06E-09 | adenocarcinoma |
| ILMN_3132361 | 27057 | Ncoa4 | -1.197 | 10.067 | 1.07E-09 | adenocarcinoma |
| ILMN_2502542 | 107652 | Uap1 | -1.294 | 11.432 | 1.07E-09 | adenocarcinoma |
| ILMN_2706205 | 52637 | Cisd1 | -1.595 | 10.336 | 1.07E-09 | adenocarcinoma |
| ILMN_2426853 | 24108 | Ubd | -1.727 | 9.338 | 1.08E-09 | adenocarcinoma |
| ILMN_1225995 | 74522 | Morc2a | 1.161 | 10.488 | 1.11E-09 | adenocarcinoma |
| ILMN_2896601 | 15894 | Icam1 | 1.403 | 8.817 | 1.11E-09 | adenocarcinoma |
| ILMN_2674122 | 18767 | Pkia | -1.047 | 9.203 | 1.11E-09 | adenocarcinoma |
| ILMN_2656645 | 100532 | Rell1 | 1.264 | 9.429 | 1.12E-09 | adenocarcinoma |
| ILMN_1225605 | 18703 | Pigr | -1.525 | 11.003 | 1.14E-09 | adenocarcinoma |
| ILMN_1230423 | 268396 | Sh3pxd2b | 1.403 | 9.090 | 1.14E-09 | adenocarcinoma |
| ILMN_3123473 | 56454 | Aldh18a1 | 1.161 | 8.892 | 1.14E-09 | adenocarcinoma |
| ILMN_1247530 | 16897 | Llgl1 | 1.515 | 11.670 | 1.15E-09 | adenocarcinoma |
| ILMN_2967750 | 72125 | Amer2 | 1.378 | 8.151 | 1.15E-09 | adenocarcinoma |
| ILMN_2529932 | 19167 | Psma3 | 1.326 | 12.099 | 1.15E-09 | adenocarcinoma |
| ILMN_1238736 | NA | NA | -2.069 | 14.335 | 1.16E-09 | adenocarcinoma |
| ILMN_2647172 | 68607 | Serhl | -1.320 | 10.863 | 1.16E-09 | adenocarcinoma |
| ILMN_2803399 | 17219 | Mcm6 | 1.426 | 8.679 | 1.18E-09 | adenocarcinoma |
| ILMN_1225615 | 83701 | Srrt | 1.250 | 9.122 | 1.19E-09 | adenocarcinoma |
| ILMN_1214126 | 55927 | Hes6 | 1.514 | 9.506 | 1.21E-09 | adenocarcinoma |
| ILMN_3114124 | 101502 | Hsd3b7 | 1.054 | 11.012 | 1.21E-09 | adenocarcinoma |
| ILMN_2855590 | 245877 | Map7d1 | 1.243 | 10.982 | 1.21E-09 | adenocarcinoma |
| ILMN_3108770 | 30841 | Kdm2b | 1.204 | 9.547 | 1.22E-09 | adenocarcinoma |
| ILMN_2596396 | 56149 | Grasp | 1.282 | 9.036 | 1.22E-09 | adenocarcinoma |
| ILMN_1246390 | 20516 | Slc20a2 | 1.371 | 9.945 | 1.24E-09 | adenocarcinoma |
| ILMN_2872698 | 70827 | Trak2 | 1.017 | 10.290 | 1.24E-09 | adenocarcinoma |
| ILMN_1224077 | 22324 | Vav1 | 1.045 | 9.504 | 1.25E-09 | adenocarcinoma |
| ILMN_2970532 | 70024 | Mcm10 | 1.465 | 8.949 | 1.28E-09 | adenocarcinoma |
| ILMN_2640330 | 12505 | Cd44 | 1.005 | 8.231 | 1.29E-09 | adenocarcinoma |
| ILMN_2674483 | 14776 | Gpx2 | 2.341 | 12.900 | 1.31E-09 | adenocarcinoma |
| ILMN_2675833 | 13393 | Dlx3 | 1.532 | 8.357 | 1.35E-09 | adenocarcinoma |
| ILMN_1221750 | 16918 | Mycl | 2.119 | 10.572 | 1.37E-09 | adenocarcinoma |
| ILMN_1227845 | 228802 | Bpifb5 | 1.958 | 8.338 | 1.38E-09 | adenocarcinoma |
| ILMN_2641647 | 21416 | Tcf7l2 | -1.060 | 11.644 | 1.38E-09 | adenocarcinoma |
| ILMN_1223949 | 12740 | Cldn4 | 2.756 | 12.451 | 1.38E-09 | adenocarcinoma |
| ILMN_2634777 | 207181 | Rbms3 | 2.050 | 8.870 | 1.38E-09 | adenocarcinoma |
| ILMN_2647628 | 19046 | Ppp1cb | -1.227 | 9.974 | 1.38E-09 | adenocarcinoma |
| ILMN_2908846 | 544963 | Iqgap2 | -1.525 | 11.356 | 1.39E-09 | adenocarcinoma |
| ILMN_2439044 | 67203 | Nde1 | 1.357 | 9.397 | 1.40E-09 | adenocarcinoma |
| ILMN_2770585 | 232491 | Pyroxd1 | -1.161 | 10.830 | 1.40E-09 | adenocarcinoma |
| ILMN_2594926 | 13088 | Cyp2b10 | 1.369 | 8.410 | 1.40E-09 | adenocarcinoma |
| ILMN_2647856 | 102871 | D330045A20Rik | -1.509 | 9.040 | 1.41E-09 | adenocarcinoma |
| ILMN_1236079 | 229599 | Ciart | -1.130 | 8.215 | 1.43E-09 | adenocarcinoma |
| ILMN_3004864 | 223697 | Sun2 | -1.367 | 11.204 | 1.43E-09 | adenocarcinoma |
| ILMN_2605645 | 227753 | Gsn | -1.594 | 10.471 | 1.43E-09 | adenocarcinoma |
| ILMN_1219447 | 56364 | Zmym3 | 1.030 | 10.144 | 1.43E-09 | adenocarcinoma |
| ILMN_2947526 | 13601 | Ecm1 | 1.853 | 10.454 | 1.44E-09 | adenocarcinoma |
| ILMN_3075043 | 20216 | Acsm3 | -1.307 | 9.154 | 1.45E-09 | adenocarcinoma |
| ILMN_2419998 | 20652 | Soat1 | 1.193 | 9.039 | 1.45E-09 | adenocarcinoma |
| ILMN_2655373 | 50935 | St6galnac6 | -1.536 | 10.673 | 1.47E-09 | adenocarcinoma |
| ILMN_1224635 | 66120 | Fkbp11 | 1.079 | 9.418 | 1.48E-09 | adenocarcinoma |
| ILMN_1244161 | 56520 | Nme4 | 1.287 | 8.513 | 1.48E-09 | adenocarcinoma |
| ILMN_2731509 | 13216 | Defa1 | 1.648 | 8.279 | 1.48E-09 | adenocarcinoma |
| ILMN_2972627 | 66847 | Hint3 | -1.262 | 11.251 | 1.49E-09 | adenocarcinoma |
| ILMN_1255745 | 67808 | Tprgl | -1.142 | 9.584 | 1.49E-09 | adenocarcinoma |
| ILMN_2609868 | 102502 | Pls1 | -1.331 | 11.676 | 1.50E-09 | adenocarcinoma |
| ILMN_2531881 | 64381 | Ms4a8a | -1.539 | 11.174 | 1.50E-09 | adenocarcinoma |
| ILMN_1233857 | 17219 | Mcm6 | 1.306 | 8.771 | 1.51E-09 | adenocarcinoma |
| ILMN_1254786 | 51798 | Ech1 | -1.188 | 13.477 | 1.52E-09 | adenocarcinoma |
| ILMN_2760800 | 57266 | Cxcl14 | 2.096 | 8.646 | 1.52E-09 | adenocarcinoma |
| ILMN_2666483 | 56505 | Ruvbl1 | 1.134 | 9.869 | 1.52E-09 | adenocarcinoma |
| ILMN_2621649 | 71862 | Gpr160 | -1.405 | 9.464 | 1.53E-09 | adenocarcinoma |
| ILMN_2862111 | 15331 | Hmgn2 | 1.403 | 12.575 | 1.54E-09 | adenocarcinoma |
| ILMN_2595815 | NA | NA | -1.057 | 8.959 | 1.55E-09 | adenocarcinoma |
| ILMN_2885990 | 53318 | Pdlim3 | -2.391 | 11.365 | 1.56E-09 | adenocarcinoma |
| ILMN_2826916 | 19267 | Ptpre | 1.225 | 10.037 | 1.56E-09 | adenocarcinoma |
| ILMN_2799590 | 223646 | Naprt1 | -1.456 | 10.653 | 1.61E-09 | adenocarcinoma |
| ILMN_1230479 | 13846 | Ephb4 | 1.215 | 9.169 | 1.62E-09 | adenocarcinoma |
| ILMN_3122961 | 14469 | Gbp2 | -2.301 | 10.911 | 1.63E-09 | adenocarcinoma |
| ILMN_2580947 | NA | NA | -1.102 | 9.095 | 1.63E-09 | adenocarcinoma |
| ILMN_1238179 | 69718 | Ipmk | -1.345 | 10.198 | 1.63E-09 | adenocarcinoma |
| ILMN_2513173 | 72655 | Snhg5 | 1.200 | 9.680 | 1.63E-09 | adenocarcinoma |
| ILMN_2999642 | 217431 | Nol10 | 1.006 | 9.553 | 1.64E-09 | adenocarcinoma |
| ILMN_2789900 | 68891 | Cd177 | 1.994 | 10.579 | 1.72E-09 | adenocarcinoma |
| ILMN_1227459 | 74105 | Gga2 | 1.027 | 9.415 | 1.74E-09 | adenocarcinoma |
| ILMN_2672113 | 72108 | Ddhd2 | 1.262 | 9.995 | 1.74E-09 | adenocarcinoma |
| ILMN_2706677 | 102871 | D330045A20Rik | -1.213 | 8.811 | 1.76E-09 | adenocarcinoma |
| ILMN_1217003 | 233038 | Nccrp1 | -1.339 | 9.001 | 1.76E-09 | adenocarcinoma |
| ILMN_1248959 | 26944 | Tinag | -1.581 | 10.452 | 1.77E-09 | adenocarcinoma |
| ILMN_1242239 | 83701 | Srrt | 1.369 | 9.891 | 1.79E-09 | adenocarcinoma |
| ILMN_2657141 | 20930 | Surf1 | -1.079 | 10.198 | 1.80E-09 | adenocarcinoma |
| ILMN_2617194 | 218772 | Rarb | 1.119 | 8.284 | 1.80E-09 | adenocarcinoma |
| ILMN_2535680 | NA | NA | -1.045 | 9.397 | 1.81E-09 | adenocarcinoma |
| ILMN_2827729 | 12308 | Calb2 | -1.516 | 9.049 | 1.81E-09 | adenocarcinoma |
| ILMN_1235230 | 53318 | Pdlim3 | -1.998 | 10.022 | 1.82E-09 | adenocarcinoma |
| ILMN_1243690 | 19205 | Ptbp1 | 1.070 | 10.153 | 1.82E-09 | adenocarcinoma |
| ILMN_2601833 | 234779 | Plcg2 | 1.222 | 9.176 | 1.83E-09 | adenocarcinoma |
| ILMN_2683560 | 26365 | Ceacam1 | -1.821 | 13.381 | 1.86E-09 | adenocarcinoma |
| ILMN_1252297 | 23954 | Nek3 | -1.464 | 11.264 | 1.89E-09 | adenocarcinoma |
| ILMN_2998658 | 170942 | Erdr1 | 1.893 | 10.947 | 1.92E-09 | adenocarcinoma |
| ILMN_2677056 | 56398 | Chp1 | -1.283 | 11.657 | 1.93E-09 | adenocarcinoma |
| ILMN_1224637 | 15220 | Foxq1 | 1.878 | 10.797 | 1.94E-09 | adenocarcinoma |
| ILMN_2604835 | 244886 | AI118078 | 1.073 | 8.281 | 1.95E-09 | adenocarcinoma |
| ILMN_2813830 | 23959 | Nt5e | -1.327 | 12.572 | 1.95E-09 | adenocarcinoma |
| ILMN_2774596 | 226519 | Lamc1 | 1.120 | 9.783 | 1.95E-09 | adenocarcinoma |
| ILMN_2601639 | 30877 | Gnl3 | 1.557 | 11.795 | 1.95E-09 | adenocarcinoma |
| ILMN_2550240 | NA | NA | -1.273 | 10.276 | 1.96E-09 | adenocarcinoma |
| ILMN_1253395 | NA | NA | 1.148 | 14.743 | 1.97E-09 | adenocarcinoma |
| ILMN_2712792 | 27373 | Csnk1e | 1.532 | 10.069 | 1.99E-09 | adenocarcinoma |
| ILMN_2689731 | 319520 | Dusp4 | 1.331 | 8.637 | 1.99E-09 | adenocarcinoma |
| ILMN_1214703 | NA | NA | -1.540 | 10.944 | 2.01E-09 | adenocarcinoma |
| ILMN_3123836 | 67528 | Nudt7 | -1.164 | 10.652 | 2.02E-09 | adenocarcinoma |
| ILMN_2679077 | 52530 | Nhp2 | 1.278 | 11.053 | 2.05E-09 | adenocarcinoma |
| ILMN_3001540 | 17022 | Lum | -2.810 | 10.431 | 2.05E-09 | adenocarcinoma |
| ILMN_1253237 | 18569 | Pdcd4 | -1.243 | 11.987 | 2.08E-09 | adenocarcinoma |
| ILMN_1213074 | NA | NA | -2.394 | 13.300 | 2.08E-09 | adenocarcinoma |
| ILMN_2612423 | 15384 | Hnrnpab | 1.302 | 13.664 | 2.10E-09 | adenocarcinoma |
| ILMN_2731191 | 16622 | Klk1b5 | -2.010 | 13.622 | 2.15E-09 | adenocarcinoma |
| ILMN_2698361 | 83701 | Srrt | 1.407 | 10.156 | 2.19E-09 | adenocarcinoma |
| ILMN_2966386 | 59028 | Rcl1 | 1.117 | 10.218 | 2.22E-09 | adenocarcinoma |
| ILMN_2686353 | 54200 | Sult2b1 | -1.189 | 9.213 | 2.23E-09 | adenocarcinoma |
| ILMN_1258770 | 69358 | Lrrc51 | -1.430 | 9.778 | 2.25E-09 | adenocarcinoma |
| ILMN_1234339 | 268663 | Cdhr2 | -1.274 | 10.616 | 2.25E-09 | adenocarcinoma |
| ILMN_2769680 | 67442 | Retsat | -1.970 | 10.679 | 2.27E-09 | adenocarcinoma |
| ILMN_1223179 | 15040 | H2-T23 | -1.988 | 10.537 | 2.28E-09 | adenocarcinoma |
| ILMN_1256614 | 64381 | Ms4a8a | -1.482 | 12.990 | 2.29E-09 | adenocarcinoma |
| ILMN_2851005 | 57741 | Noc2l | 1.460 | 9.851 | 2.29E-09 | adenocarcinoma |
| ILMN_2768755 | 56233 | Hdac7 | 1.332 | 8.405 | 2.30E-09 | adenocarcinoma |
| ILMN_2707227 | 71664 | Mettl7b | -1.710 | 10.794 | 2.31E-09 | adenocarcinoma |
| ILMN_2594971 | 67671 | Rpl38 | 1.239 | 9.716 | 2.32E-09 | adenocarcinoma |
| ILMN_2800256 | 217995 | Heatr1 | 1.079 | 9.403 | 2.34E-09 | adenocarcinoma |
| ILMN_1219704 | 625286 | Tmem236 | -1.545 | 9.210 | 2.34E-09 | adenocarcinoma |
| ILMN_2689333 | 27373 | Csnk1e | 1.542 | 10.319 | 2.36E-09 | adenocarcinoma |
| ILMN_2899787 | 19732 | Rgl2 | 1.096 | 8.825 | 2.37E-09 | adenocarcinoma |
| ILMN_2664155 | 209027 | Pycr1 | 1.286 | 8.165 | 2.37E-09 | adenocarcinoma |
| ILMN_2623951 | 11758 | Prdx6 | -1.125 | 8.453 | 2.42E-09 | adenocarcinoma |
| ILMN_2736838 | 77668 | 9130209A04Rik | -1.004 | 8.974 | 2.42E-09 | adenocarcinoma |
| ILMN_2784446 | 20671 | Sox17 | 1.229 | 8.274 | 2.44E-09 | adenocarcinoma |
| ILMN_1220418 | 23900 | Hcst | -1.398 | 9.947 | 2.45E-09 | adenocarcinoma |
| ILMN_2789762 | 52793 | Fam3b | -1.540 | 9.911 | 2.45E-09 | adenocarcinoma |
| ILMN_1255834 | 98267 | Stk17b | -1.877 | 11.312 | 2.45E-09 | adenocarcinoma |
| ILMN_2723965 | 13615 | Edn2 | -1.746 | 10.362 | 2.45E-09 | adenocarcinoma |
| ILMN_1247071 | 20517 | Slc22a1 | 1.639 | 10.424 | 2.46E-09 | adenocarcinoma |
| ILMN_2900557 | 209737 | Kif15 | 1.632 | 8.374 | 2.47E-09 | adenocarcinoma |
| ILMN_1221736 | 56045 | Samhd1 | -1.654 | 9.924 | 2.47E-09 | adenocarcinoma |
| ILMN_3097726 | 30877 | Gnl3 | 1.421 | 10.111 | 2.47E-09 | adenocarcinoma |
| ILMN_1223102 | 223646 | Naprt1 | -1.077 | 9.026 | 2.47E-09 | adenocarcinoma |
| ILMN_2626401 | 75723 | Amotl1 | 1.170 | 8.986 | 2.48E-09 | adenocarcinoma |
| ILMN_2690677 | 23844 | Clca3 | -1.923 | 15.074 | 2.49E-09 | adenocarcinoma |
| ILMN_1240264 | 53376 | Usp2 | -1.606 | 9.192 | 2.49E-09 | adenocarcinoma |
| ILMN_2757682 | 243813 | Leng9 | -1.946 | 11.964 | 2.49E-09 | adenocarcinoma |
| ILMN_3140913 | 381605 | Tbc1d2 | -1.292 | 11.732 | 2.49E-09 | adenocarcinoma |
| ILMN_1245307 | 14115 | Fbln2 | -1.099 | 9.028 | 2.50E-09 | adenocarcinoma |
| ILMN_2736347 | 214572 | Prmt7 | 1.396 | 10.729 | 2.50E-09 | adenocarcinoma |
| ILMN_2638509 | 73647 | Capn9 | -1.584 | 10.937 | 2.52E-09 | adenocarcinoma |
| ILMN_1241142 | 94040 | Clmn | -1.408 | 10.891 | 2.52E-09 | adenocarcinoma |
| ILMN_2605212 | 102857 | Slc6a8 | -1.370 | 12.433 | 2.55E-09 | adenocarcinoma |
| ILMN_2431150 | NA | NA | -1.108 | 9.577 | 2.55E-09 | adenocarcinoma |
| ILMN_1213191 | 215387 | Ncaph | 1.359 | 8.445 | 2.57E-09 | adenocarcinoma |
| ILMN_2497190 | 171543 | Bmf | 1.170 | 8.481 | 2.62E-09 | adenocarcinoma |
| ILMN_2420070 | 103733 | Tubg1 | 1.229 | 9.444 | 2.63E-09 | adenocarcinoma |
| ILMN_2449353 | 26931 | Ppp2r5c | -1.038 | 8.947 | 2.63E-09 | adenocarcinoma |
| ILMN_2658961 | 13139 | Dgka | -1.308 | 11.952 | 2.66E-09 | adenocarcinoma |
| ILMN_2858359 | 269582 | Clspn | 1.180 | 8.262 | 2.66E-09 | adenocarcinoma |
| ILMN_2642426 | 75986 | Agmat | 1.879 | 8.438 | 2.67E-09 | adenocarcinoma |
| ILMN_1236693 | 67118 | Bfar | -1.140 | 9.535 | 2.69E-09 | adenocarcinoma |
| ILMN_1232170 | 171508 | Creld1 | 1.296 | 9.504 | 2.71E-09 | adenocarcinoma |
| ILMN_2814865 | 70536 | Qpct | -1.985 | 10.197 | 2.73E-09 | adenocarcinoma |
| ILMN_3009010 | 52615 | Suz12 | 1.105 | 11.876 | 2.75E-09 | adenocarcinoma |
| ILMN_1224003 | 19205 | Ptbp1 | 1.383 | 12.510 | 2.81E-09 | adenocarcinoma |
| ILMN_1245496 | 224762 | Trim31 | -1.818 | 11.527 | 2.82E-09 | adenocarcinoma |
| ILMN_2700699 | 67867 | Lrrc28 | 1.109 | 9.534 | 2.83E-09 | adenocarcinoma |
| ILMN_2971744 | 11545 | Parp1 | 1.592 | 10.382 | 2.89E-09 | adenocarcinoma |
| ILMN_2666487 | 56505 | Ruvbl1 | 1.211 | 9.936 | 2.92E-09 | adenocarcinoma |
| ILMN_2534332 | 16898 | Rps2 | 1.003 | 13.871 | 3.13E-09 | adenocarcinoma |
| ILMN_2859978 | 23971 | Papss1 | -1.105 | 12.557 | 3.16E-09 | adenocarcinoma |
| ILMN_2418811 | 72275 | 2200002D01Rik | -1.437 | 12.052 | 3.16E-09 | adenocarcinoma |
| ILMN_2664686 | 110749 | Chaf1b | 1.717 | 9.103 | 3.17E-09 | adenocarcinoma |
| ILMN_1255736 | 269523 | Vcp | 1.115 | 13.803 | 3.20E-09 | adenocarcinoma |
| ILMN_3114585 | 12505 | Cd44 | 1.430 | 8.986 | 3.21E-09 | adenocarcinoma |
| ILMN_2612283 | 14461 | Gata2 | 1.668 | 8.612 | 3.21E-09 | adenocarcinoma |
| ILMN_1257631 | 11810 | Apobec1 | -1.531 | 12.004 | 3.24E-09 | adenocarcinoma |
| ILMN_2952661 | 27214 | Dbf4 | 1.225 | 8.638 | 3.30E-09 | adenocarcinoma |
| ILMN_1251000 | 21333 | Tac1 | -1.377 | 9.833 | 3.31E-09 | adenocarcinoma |
| ILMN_2905386 | 11740 | Slc25a5 | -1.370 | 13.845 | 3.31E-09 | adenocarcinoma |
| ILMN_2612513 | 229707 | Strip1 | 1.211 | 12.502 | 3.32E-09 | adenocarcinoma |
| ILMN_1256080 | 56734 | Tulp2 | 1.321 | 8.637 | 3.39E-09 | adenocarcinoma |
| ILMN_2838372 | 72061 | 2010111I01Rik | 1.241 | 9.818 | 3.40E-09 | adenocarcinoma |
| ILMN_2987709 | 65221 | Slc15a3 | 1.049 | 8.259 | 3.42E-09 | adenocarcinoma |
| ILMN_1242418 | 20459 | Ptk6 | -1.143 | 8.936 | 3.43E-09 | adenocarcinoma |
| ILMN_1230039 | 15587 | Hyal2 | 1.200 | 12.724 | 3.50E-09 | adenocarcinoma |
| ILMN_2617335 | 66046 | Ndufb5 | -1.010 | 9.780 | 3.58E-09 | adenocarcinoma |
| ILMN_2928320 | 11972 | Atp6v0d1 | -1.012 | 11.721 | 3.58E-09 | adenocarcinoma |
| ILMN_1230454 | 231086 | Hadhb | -1.559 | 11.117 | 3.58E-09 | adenocarcinoma |
| ILMN_1234959 | 107823 | Whsc1 | 1.056 | 9.133 | 3.58E-09 | adenocarcinoma |
| ILMN_2728118 | 107094 | Rrp12 | 1.220 | 9.190 | 3.60E-09 | adenocarcinoma |
| ILMN_1245650 | 66711 | Sbds | -1.000 | 13.383 | 3.65E-09 | adenocarcinoma |
| ILMN_2617858 | 18426 | Ovol1 | -1.458 | 9.684 | 3.66E-09 | adenocarcinoma |
| ILMN_1233220 | 68755 | Cgrrf1 | -1.089 | 9.448 | 3.72E-09 | adenocarcinoma |
| ILMN_2637165 | 76432 | 2310001H17Rik | -1.109 | 9.294 | 3.73E-09 | adenocarcinoma |
| ILMN_1232099 | 56434 | Tspan3 | -1.361 | 12.625 | 3.74E-09 | adenocarcinoma |
| ILMN_2866189 | 12226 | Btg1 | -1.391 | 13.713 | 3.75E-09 | adenocarcinoma |
| ILMN_1241857 | 53605 | Nap1l1 | 1.222 | 10.257 | 3.76E-09 | adenocarcinoma |
| ILMN_2710353 | 11475 | Acta2 | -2.117 | 10.622 | 3.77E-09 | adenocarcinoma |
| ILMN_2836137 | 242705 | E2f2 | 1.403 | 9.045 | 3.95E-09 | adenocarcinoma |
| ILMN_1213351 | 57911 | Gsdma | 1.578 | 8.581 | 3.95E-09 | adenocarcinoma |
| ILMN_2705166 | 20440 | St6gal1 | -1.851 | 12.435 | 4.00E-09 | adenocarcinoma |
| ILMN_2759914 | 20278 | Scnn1g | -1.217 | 9.383 | 4.05E-09 | adenocarcinoma |
| ILMN_1220996 | 15170 | Ptpn6 | 1.228 | 10.517 | 4.07E-09 | adenocarcinoma |
| ILMN_1246495 | 56786 | Tmem9b | -1.056 | 10.134 | 4.10E-09 | adenocarcinoma |
| ILMN_2998313 | 320405 | Cadps2 | -1.255 | 12.577 | 4.21E-09 | adenocarcinoma |
| ILMN_2672772 | 57742 | Abhd1 | -1.152 | 9.730 | 4.26E-09 | adenocarcinoma |
| ILMN_2724545 | 104175 | Sbk1 | 1.150 | 10.554 | 4.28E-09 | adenocarcinoma |
| ILMN_2710139 | 19017 | Ppargc1a | -2.160 | 10.877 | 4.28E-09 | adenocarcinoma |
| ILMN_2700166 | 12444 | Ccnd2 | 1.447 | 9.917 | 4.34E-09 | adenocarcinoma |
| ILMN_2798086 | 207278 | Fchsd2 | -1.141 | 12.018 | 4.42E-09 | adenocarcinoma |
| ILMN_2829330 | 15212 | Hexb | -1.425 | 10.651 | 4.43E-09 | adenocarcinoma |
| ILMN_2620145 | 72469 | Plcd3 | 1.029 | 8.130 | 4.50E-09 | adenocarcinoma |
| ILMN_1228937 | 54151 | Cyhr1 | -1.194 | 12.035 | 4.51E-09 | adenocarcinoma |
| ILMN_1227128 | 20667 | Sox12 | 1.118 | 8.443 | 4.51E-09 | adenocarcinoma |
| ILMN_1244291 | 14609 | Gja1 | 1.591 | 11.446 | 4.52E-09 | adenocarcinoma |
| ILMN_1227061 | 70163 | Lypd8 | -1.653 | 11.388 | 4.53E-09 | adenocarcinoma |
| ILMN_1222773 | 20336 | Exoc4 | 1.156 | 9.525 | 4.53E-09 | adenocarcinoma |
| ILMN_2687828 | 13496 | Arid3a | 1.060 | 8.323 | 4.53E-09 | adenocarcinoma |
| ILMN_1227357 | 14252 | Flot2 | 1.174 | 10.257 | 4.54E-09 | adenocarcinoma |
| ILMN_2597778 | 14675 | Gna14 | -1.695 | 11.530 | 4.58E-09 | adenocarcinoma |
| ILMN_1223285 | 15512 | Hspa2 | 1.104 | 10.164 | 4.63E-09 | adenocarcinoma |
| ILMN_2768154 | 20208 | Saa1 | -2.552 | 14.618 | 4.84E-09 | adenocarcinoma |
| ILMN_2729607 | 20276 | Scnn1a | -1.571 | 10.062 | 4.86E-09 | adenocarcinoma |
| ILMN_2519802 | NA | NA | -1.033 | 9.432 | 4.88E-09 | adenocarcinoma |
| ILMN_2669441 | 20624 | Eftud2 | 1.041 | 9.485 | 4.89E-09 | adenocarcinoma |
| ILMN_2429203 | 27801 | Zdhhc8 | 1.117 | 10.582 | 4.90E-09 | adenocarcinoma |
| ILMN_2904435 | 67133 | Gp2 | -2.438 | 10.004 | 4.93E-09 | adenocarcinoma |
| ILMN_2589318 | 19009 | Pou6f1 | 1.225 | 8.906 | 4.94E-09 | adenocarcinoma |
| ILMN_2713438 | 209601 | 4922501L14Rik | 1.347 | 8.317 | 4.95E-09 | adenocarcinoma |
| ILMN_2588882 | 338365 | Slc41a2 | -1.011 | 9.307 | 5.10E-09 | adenocarcinoma |
| ILMN_1235506 | NA | NA | -2.579 | 12.620 | 5.11E-09 | adenocarcinoma |
| ILMN_2690676 | 23844 | Clca3 | -1.949 | 15.253 | 5.17E-09 | adenocarcinoma |
| ILMN_2643377 | 170756 | Slc8b1 | -1.366 | 11.433 | 5.17E-09 | adenocarcinoma |
| ILMN_2417863 | 57783 | Tnip1 | -1.612 | 11.746 | 5.30E-09 | adenocarcinoma |
| ILMN_1247916 | 225341 | Lims2 | -1.147 | 8.899 | 5.35E-09 | adenocarcinoma |
| ILMN_1251109 | 107375 | Slc25a45 | 1.379 | 9.554 | 5.40E-09 | adenocarcinoma |
| ILMN_2893471 | 319582 | 6430573F11Rik | 1.020 | 8.418 | 5.41E-09 | adenocarcinoma |
| ILMN_2936427 | 17217 | Mcm4 | 1.640 | 10.977 | 5.46E-09 | adenocarcinoma |
| ILMN_2679770 | 78523 | Mrpl9 | -1.025 | 11.392 | 5.66E-09 | adenocarcinoma |
| ILMN_2732554 | 13216 | Defa1 | 1.496 | 8.055 | 5.66E-09 | adenocarcinoma |
| ILMN_1255053 | 18817 | Plk1 | 2.030 | 9.043 | 5.79E-09 | adenocarcinoma |
| ILMN_2821263 | 98845 | Eps8l2 | -1.067 | 11.612 | 5.79E-09 | adenocarcinoma |
| ILMN_2640441 | 15182 | Hdac2 | 1.036 | 11.445 | 5.81E-09 | adenocarcinoma |
| ILMN_2778872 | 56150 | Mad2l1 | 1.912 | 9.324 | 5.91E-09 | adenocarcinoma |
| ILMN_2693403 | 109901 | Cela1 | -1.412 | 12.957 | 6.01E-09 | adenocarcinoma |
| ILMN_1221568 | 66953 | Cdca7 | 1.422 | 10.561 | 6.04E-09 | adenocarcinoma |
| ILMN_1228330 | 224860 | Plcl2 | -1.288 | 10.324 | 6.07E-09 | adenocarcinoma |
| ILMN_2432508 | 68427 | Slc39a13 | 1.117 | 8.849 | 6.14E-09 | adenocarcinoma |
| ILMN_2764781 | 102866 | Pls3 | 1.003 | 8.728 | 6.28E-09 | adenocarcinoma |
| ILMN_2818396 | 67759 | Plgrkt | -1.593 | 12.719 | 6.35E-09 | adenocarcinoma |
| ILMN_2633148 | 72141 | Adpgk | 1.102 | 9.011 | 6.42E-09 | adenocarcinoma |
| ILMN_3153207 | 16784 | Lamp2 | 1.681 | 11.708 | 6.44E-09 | adenocarcinoma |
| ILMN_2646203 | 12567 | Cdk4 | 1.346 | 9.090 | 6.45E-09 | adenocarcinoma |
| ILMN_2772224 | 50776 | Polg2 | -1.039 | 9.248 | 6.49E-09 | adenocarcinoma |
| ILMN_2973288 | 16772 | Lama1 | 1.426 | 8.303 | 6.49E-09 | adenocarcinoma |
| ILMN_1213958 | 20334 | Sec23a | -1.127 | 9.262 | 6.60E-09 | adenocarcinoma |
| ILMN_1216474 | NA | NA | 1.031 | 8.195 | 6.71E-09 | adenocarcinoma |
| ILMN_2622255 | 13844 | Ephb2 | 1.616 | 9.252 | 6.74E-09 | adenocarcinoma |
| ILMN_2888902 | 18537 | Pcmt1 | -1.136 | 10.844 | 6.74E-09 | adenocarcinoma |
| ILMN_1216493 | 56451 | Suclg1 | -1.570 | 12.731 | 6.75E-09 | adenocarcinoma |
| ILMN_2762640 | 216456 | Gls2 | 1.477 | 8.398 | 6.79E-09 | adenocarcinoma |
| ILMN_2653284 | 83429 | Ctns | -1.156 | 11.291 | 6.80E-09 | adenocarcinoma |
| ILMN_2758695 | 71805 | Nup93 | 1.486 | 10.505 | 6.80E-09 | adenocarcinoma |
| ILMN_1233293 | 14468 | Gbp2b | -2.535 | 11.024 | 6.86E-09 | adenocarcinoma |
| ILMN_1221920 | 263406 | Plekhg3 | 1.054 | 12.025 | 6.89E-09 | adenocarcinoma |
| ILMN_2694917 | 60406 | Sap30 | 1.162 | 11.034 | 6.91E-09 | adenocarcinoma |
| ILMN_2776721 | 14933 | Gyk | -1.514 | 9.417 | 6.93E-09 | adenocarcinoma |
| ILMN_2623793 | 11906 | Zfhx3 | 1.424 | 9.498 | 6.96E-09 | adenocarcinoma |
| ILMN_3053655 | 207818 | Smagp | -1.047 | 9.441 | 6.96E-09 | adenocarcinoma |
| ILMN_1251504 | 102294 | Cyp4v3 | -1.053 | 10.143 | 6.98E-09 | adenocarcinoma |
| ILMN_1244343 | 223697 | Sun2 | -1.302 | 10.671 | 6.98E-09 | adenocarcinoma |
| ILMN_2755443 | 58194 | Sh3kbp1 | 1.476 | 9.688 | 6.98E-09 | adenocarcinoma |
| ILMN_2987995 | 67118 | Bfar | -1.219 | 10.890 | 7.04E-09 | adenocarcinoma |
| ILMN_2766105 | 243197 | Mfsd7a | -1.103 | 9.195 | 7.07E-09 | adenocarcinoma |
| ILMN_2947187 | 103554 | Psme4 | 1.009 | 12.285 | 7.10E-09 | adenocarcinoma |
| ILMN_2881857 | 54216 | Pcdh7 | 1.013 | 8.435 | 7.15E-09 | adenocarcinoma |
| ILMN_1247592 | 12362 | Casp1 | -1.529 | 13.850 | 7.30E-09 | adenocarcinoma |
| ILMN_2766279 | 68964 | Ctc1 | 1.300 | 10.248 | 7.35E-09 | adenocarcinoma |
| ILMN_2647170 | 68607 | Serhl | -1.180 | 10.149 | 7.51E-09 | adenocarcinoma |
| ILMN_1241322 | 12365 | Casp14 | -2.037 | 10.518 | 7.59E-09 | adenocarcinoma |
| ILMN_2999654 | 19183 | Psmc3ip | 1.005 | 8.458 | 7.59E-09 | adenocarcinoma |
| ILMN_2814484 | 68713 | Ifitm1 | 1.332 | 8.117 | 7.63E-09 | adenocarcinoma |
| ILMN_2537734 | NA | NA | -1.323 | 9.484 | 7.63E-09 | adenocarcinoma |
| ILMN_2846198 | 52430 | Echdc2 | 1.461 | 8.654 | 7.71E-09 | adenocarcinoma |
| ILMN_2740890 | 73847 | Fam110a | 1.116 | 8.854 | 7.78E-09 | adenocarcinoma |
| ILMN_2923599 | 74204 | Xpo6 | 1.023 | 9.808 | 7.80E-09 | adenocarcinoma |
| ILMN_2733082 | NA | NA | -1.306 | 8.806 | 7.92E-09 | adenocarcinoma |
| ILMN_3145484 | 76787 | Ppfia3 | -1.055 | 8.830 | 8.13E-09 | adenocarcinoma |
| ILMN_1221084 | 66451 | 2610528J11Rik | -1.337 | 10.177 | 8.20E-09 | adenocarcinoma |
| ILMN_2926198 | 69573 | Hilpda | 1.517 | 9.819 | 8.20E-09 | adenocarcinoma |
| ILMN_2700265 | 18631 | Pex11a | -1.326 | 10.881 | 8.27E-09 | adenocarcinoma |
| ILMN_1231138 | 13555 | E2f1 | 1.112 | 8.553 | 8.28E-09 | adenocarcinoma |
| ILMN_2451441 | 17532 | Mras | 1.248 | 9.093 | 8.38E-09 | adenocarcinoma |
| ILMN_2704257 | 19699 | Reln | -1.502 | 9.810 | 8.43E-09 | adenocarcinoma |
| ILMN_3007669 | 53817 | Ddx39b | 1.125 | 11.196 | 8.43E-09 | adenocarcinoma |
| ILMN_1260325 | 230721 | Pabpc4 | 1.193 | 11.427 | 8.45E-09 | adenocarcinoma |
| ILMN_2748941 | 106581 | Itfg3 | -1.107 | 11.663 | 8.45E-09 | adenocarcinoma |
| ILMN_2683528 | 66569 | Gdpd1 | -1.157 | 12.927 | 8.60E-09 | adenocarcinoma |
| ILMN_2930364 | 20624 | Eftud2 | 1.040 | 9.301 | 8.60E-09 | adenocarcinoma |
| ILMN_2704048 | 109552 | Sri | -2.241 | 12.036 | 8.63E-09 | adenocarcinoma |
| ILMN_2737713 | 13614 | Edn1 | -2.169 | 12.405 | 8.63E-09 | adenocarcinoma |
| ILMN_2619249 | 244962 | Snx14 | -1.005 | 9.370 | 8.65E-09 | adenocarcinoma |
| ILMN_2654013 | 234865 | Nup133 | 1.072 | 8.996 | 8.69E-09 | adenocarcinoma |
| ILMN_2750349 | 17173 | Ascl2 | 1.015 | 8.255 | 8.69E-09 | adenocarcinoma |
| ILMN_2664628 | 13135 | Dad1 | 1.040 | 13.010 | 8.81E-09 | adenocarcinoma |
| ILMN_2954098 | 16857 | Lgals6 | -1.853 | 11.231 | 8.84E-09 | adenocarcinoma |
| ILMN_1223598 | 20788 | Srebf2 | 1.294 | 9.961 | 8.85E-09 | adenocarcinoma |
| ILMN_2649067 | 16362 | Irf1 | -1.438 | 10.109 | 8.94E-09 | adenocarcinoma |
| ILMN_1229177 | 16618 | Klk1b26 | -1.980 | 13.544 | 8.94E-09 | adenocarcinoma |
| ILMN_1240332 | 224454 | Zdhhc14 | 1.276 | 9.802 | 9.01E-09 | adenocarcinoma |
| ILMN_2759088 | 70113 | Odf3b | -1.338 | 9.962 | 9.07E-09 | adenocarcinoma |
| ILMN_1259470 | 65963 | Tmem176b | 1.701 | 10.977 | 9.09E-09 | adenocarcinoma |
| ILMN_1252399 | 21380 | Tbx1 | 1.692 | 8.543 | 9.09E-09 | adenocarcinoma |
| ILMN_2451022 | 22352 | Vim | 1.414 | 10.685 | 9.11E-09 | adenocarcinoma |
| ILMN_2588249 | 57435 | Plin4 | -1.557 | 9.426 | 9.16E-09 | adenocarcinoma |
| ILMN_3144289 | 22031 | Traf3 | 1.084 | 10.164 | 9.20E-09 | adenocarcinoma |
| ILMN_2549426 | 54613 | St3gal6 | -1.276 | 9.848 | 9.25E-09 | adenocarcinoma |
| ILMN_2629030 | 75686 | Nudt16 | -1.127 | 11.860 | 9.27E-09 | adenocarcinoma |
| ILMN_2923864 | 12368 | Casp6 | 1.031 | 9.869 | 9.37E-09 | adenocarcinoma |
| ILMN_1222594 | 66665 | Msantd3 | 1.017 | 8.371 | 9.38E-09 | adenocarcinoma |
| ILMN_2661950 | 69270 | Gins1 | 1.202 | 8.364 | 9.40E-09 | adenocarcinoma |
| ILMN_2785353 | 171095 | Il17rc | -1.271 | 10.314 | 9.46E-09 | adenocarcinoma |
| ILMN_2857684 | 106347 | Ildr1 | -1.212 | 10.287 | 9.52E-09 | adenocarcinoma |
| ILMN_2661588 | 20743 | Sptbn2 | 1.314 | 9.437 | 9.58E-09 | adenocarcinoma |
| ILMN_1259473 | 14156 | Fen1 | 1.283 | 9.582 | 9.65E-09 | adenocarcinoma |
| ILMN_1233691 | NA | NA | -1.997 | 10.658 | 9.80E-09 | adenocarcinoma |
| ILMN_2660386 | 223723 | Ttll12 | 1.079 | 9.590 | 1.00E-08 | adenocarcinoma |
| ILMN_2534090 | 216443 | Mars | 1.307 | 9.707 | 1.02E-08 | adenocarcinoma |
| ILMN_1237631 | 93737 | Pard6g | 1.139 | 9.500 | 1.02E-08 | adenocarcinoma |
| ILMN_2742849 | 17218 | Mcm5 | 1.967 | 9.769 | 1.03E-08 | adenocarcinoma |
| ILMN_2727722 | 271844 | Pla2g4f | -1.522 | 10.831 | 1.04E-08 | adenocarcinoma |
| ILMN_1247047 | 77134 | Hnrnpa0 | 1.336 | 10.585 | 1.04E-08 | adenocarcinoma |
| ILMN_1218228 | 280408 | Rilp | -1.060 | 9.647 | 1.05E-08 | adenocarcinoma |
| ILMN_2616226 | 13170 | Dbp | -3.914 | 11.046 | 1.05E-08 | adenocarcinoma |
| ILMN_1239024 | 18099 | Nlk | 1.157 | 9.678 | 1.06E-08 | adenocarcinoma |
| ILMN_2782503 | 93679 | Trim8 | 1.023 | 11.471 | 1.06E-08 | adenocarcinoma |
| ILMN_1219746 | NA | NA | -1.509 | 10.304 | 1.07E-08 | adenocarcinoma |
| ILMN_2830661 | 21973 | Top2a | 1.843 | 8.982 | 1.07E-08 | adenocarcinoma |
| ILMN_2862706 | NA | NA | -1.394 | 12.945 | 1.08E-08 | adenocarcinoma |
| ILMN_2595714 | 14268 | Fn1 | 1.395 | 10.782 | 1.08E-08 | adenocarcinoma |
| ILMN_2733193 | 107869 | Cth | -1.034 | 8.917 | 1.10E-08 | adenocarcinoma |
| ILMN_2757232 | 11829 | Aqp4 | -2.320 | 10.964 | 1.13E-08 | adenocarcinoma |
| ILMN_2741726 | 27756 | Lsm2 | 1.050 | 9.150 | 1.13E-08 | adenocarcinoma |
| ILMN_2631093 | 12520 | Cd81 | 1.035 | 13.518 | 1.13E-08 | adenocarcinoma |
| ILMN_2746576 | 13219 | Defa-rs10 | 1.791 | 8.305 | 1.13E-08 | adenocarcinoma |
| ILMN_2589785 | 71994 | Cnn3 | 1.382 | 9.687 | 1.13E-08 | adenocarcinoma |
| ILMN_3046846 | 78294 | Rps27a | 1.137 | 10.978 | 1.13E-08 | adenocarcinoma |
| ILMN_3067259 | 76787 | Ppfia3 | -1.487 | 9.585 | 1.13E-08 | adenocarcinoma |
| ILMN_2782082 | 16432 | Itm2b | -1.375 | 11.696 | 1.14E-08 | adenocarcinoma |
| ILMN_2832979 | 14734 | Gpc3 | -1.155 | 8.940 | 1.14E-08 | adenocarcinoma |
| ILMN_2737319 | 67674 | Trmt112 | 1.078 | 9.987 | 1.15E-08 | adenocarcinoma |
| ILMN_2488000 | 77519 | Zfp266 | 1.048 | 9.381 | 1.17E-08 | adenocarcinoma |
| ILMN_2517041 | 18140 | Uhrf1 | 2.064 | 9.009 | 1.19E-08 | adenocarcinoma |
| ILMN_2458053 | 319616 | 5930412G12Rik | 1.093 | 8.074 | 1.20E-08 | adenocarcinoma |
| ILMN_2561342 | 215384 | Fcgbp | -1.432 | 12.671 | 1.20E-08 | adenocarcinoma |
| ILMN_2912410 | 77996 | Cutal | -1.661 | 11.162 | 1.21E-08 | adenocarcinoma |
| ILMN_2659503 | 12554 | Cdh13 | 1.238 | 8.472 | 1.21E-08 | adenocarcinoma |
| ILMN_2580803 | NA | NA | -1.169 | 8.724 | 1.21E-08 | adenocarcinoma |
| ILMN_2673776 | 242705 | E2f2 | 1.444 | 9.032 | 1.25E-08 | adenocarcinoma |
| ILMN_2955047 | 13511 | Dsg2 | -1.256 | 11.264 | 1.25E-08 | adenocarcinoma |
| ILMN_2812215 | 269608 | Plekhg5 | 1.028 | 8.891 | 1.25E-08 | adenocarcinoma |
| ILMN_1225009 | NA | NA | -1.088 | 9.328 | 1.25E-08 | adenocarcinoma |
| ILMN_1226979 | NA | NA | 1.050 | 8.292 | 1.27E-08 | adenocarcinoma |
| ILMN_2690556 | NA | NA | 1.110 | 8.232 | 1.29E-08 | adenocarcinoma |
| ILMN_2478429 | 432516 | Myo1a | -1.201 | 11.475 | 1.30E-08 | adenocarcinoma |
| ILMN_1256967 | 18750 | Prkca | -1.636 | 11.517 | 1.30E-08 | adenocarcinoma |
| ILMN_2543688 | 100616095 | 0610007N19Rik | -1.190 | 11.930 | 1.30E-08 | adenocarcinoma |
| ILMN_2723058 | 26433 | Plod3 | 1.076 | 11.412 | 1.30E-08 | adenocarcinoma |
| ILMN_2662264 | 76960 | Bcas1 | -1.345 | 13.205 | 1.30E-08 | adenocarcinoma |
| ILMN_2706730 | 19279 | Ptprr | -1.263 | 9.358 | 1.30E-08 | adenocarcinoma |
| ILMN_2728088 | 72269 | Cda | -1.375 | 9.741 | 1.32E-08 | adenocarcinoma |
| ILMN_2477540 | 18762 | Prkcz | -1.239 | 10.545 | 1.35E-08 | adenocarcinoma |
| ILMN_3002872 | 56451 | Suclg1 | -1.072 | 13.871 | 1.35E-08 | adenocarcinoma |
| ILMN_2986899 | 11534 | Adk | -1.096 | 13.314 | 1.35E-08 | adenocarcinoma |
| ILMN_2437470 | 14583 | Gfpt1 | -1.509 | 13.726 | 1.36E-08 | adenocarcinoma |
| ILMN_2512740 | 107770 | Tm6sf2 | -1.455 | 9.556 | 1.37E-08 | adenocarcinoma |
| ILMN_2689784 | 17207 | Mcf2l | -1.029 | 10.478 | 1.37E-08 | adenocarcinoma |
| ILMN_1237767 | 15378 | Hnf4a | -1.268 | 12.480 | 1.38E-08 | adenocarcinoma |
| ILMN_2826264 | 230779 | Serinc2 | 1.296 | 10.776 | 1.38E-08 | adenocarcinoma |
| ILMN_2695344 | 171095 | Il17rc | -1.244 | 10.671 | 1.39E-08 | adenocarcinoma |
| ILMN_2669804 | 66841 | Etfdh | -1.280 | 10.675 | 1.40E-08 | adenocarcinoma |
| ILMN_2762728 | 113868 | Acaa1a | -1.116 | 13.021 | 1.40E-08 | adenocarcinoma |
| ILMN_2632665 | 12389 | Cav1 | -1.711 | 10.697 | 1.40E-08 | adenocarcinoma |
| ILMN_2604282 | 20377 | Sfrp1 | -1.699 | 9.633 | 1.40E-08 | adenocarcinoma |
| ILMN_1218891 | 68636 | Fahd1 | -1.015 | 10.378 | 1.42E-08 | adenocarcinoma |
| ILMN_2774435 | 67204 | Eif2s2 | -1.197 | 9.526 | 1.45E-08 | adenocarcinoma |
| ILMN_1224023 | 99662 | Eps8l3 | -1.249 | 11.683 | 1.46E-08 | adenocarcinoma |
| ILMN_2769065 | 67680 | Sdhb | -1.214 | 11.446 | 1.46E-08 | adenocarcinoma |
| ILMN_2695199 | 54613 | St3gal6 | -1.143 | 13.133 | 1.48E-08 | adenocarcinoma |
| ILMN_1254358 | 16011 | Igfbp5 | 1.883 | 11.559 | 1.48E-08 | adenocarcinoma |
| ILMN_2762397 | 66822 | Fbxo25 | -1.432 | 10.979 | 1.50E-08 | adenocarcinoma |
| ILMN_2628603 | 77569 | Limch1 | 1.171 | 8.603 | 1.50E-08 | adenocarcinoma |
| ILMN_1246153 | 170942 | Erdr1 | 1.760 | 9.268 | 1.51E-08 | adenocarcinoma |
| ILMN_2792868 | 233016 | Blvrb | 1.078 | 11.801 | 1.51E-08 | adenocarcinoma |
| ILMN_3161959 | 623474 | Rad54b | 1.362 | 8.623 | 1.51E-08 | adenocarcinoma |
| ILMN_2550164 | 13521 | Slc26a2 | -1.367 | 8.843 | 1.51E-08 | adenocarcinoma |
| ILMN_2609614 | 71982 | Snx10 | 1.193 | 9.749 | 1.53E-08 | adenocarcinoma |
| ILMN_2758545 | 71908 | Cldn23 | -1.118 | 12.111 | 1.53E-08 | adenocarcinoma |
| ILMN_1219471 | 107589 | Mylk | -1.354 | 11.879 | 1.54E-08 | adenocarcinoma |
| ILMN_1254938 | 100434 | Slc44a1 | -1.047 | 9.225 | 1.54E-08 | adenocarcinoma |
| ILMN_3138499 | 57279 | Slc25a20 | -1.549 | 13.038 | 1.54E-08 | adenocarcinoma |
| ILMN_1242167 | NA | NA | -1.028 | 12.732 | 1.55E-08 | adenocarcinoma |
| ILMN_2439638 | 22031 | Traf3 | 1.091 | 9.897 | 1.55E-08 | adenocarcinoma |
| ILMN_2954474 | 18606 | Enpp2 | -1.244 | 9.012 | 1.56E-08 | adenocarcinoma |
| ILMN_2718314 | 26456 | Sema4g | -1.340 | 10.595 | 1.57E-08 | adenocarcinoma |
| ILMN_2645048 | 59079 | Erbb2ip | -1.076 | 9.602 | 1.57E-08 | adenocarcinoma |
| ILMN_1237216 | 15331 | Hmgn2 | 1.326 | 11.744 | 1.57E-08 | adenocarcinoma |
| ILMN_2703099 | 75751 | Ipo4 | 1.007 | 9.134 | 1.58E-08 | adenocarcinoma |
| ILMN_1238221 | 15516 | Hsp90ab1 | 1.674 | 12.919 | 1.59E-08 | adenocarcinoma |
| ILMN_2597868 | 19141 | Lgmn | 1.111 | 11.421 | 1.60E-08 | adenocarcinoma |
| ILMN_1216007 | 68202 | Ndufa5 | -1.255 | 12.753 | 1.60E-08 | adenocarcinoma |
| ILMN_2723639 | 107975 | Pacs1 | 1.123 | 8.722 | 1.61E-08 | adenocarcinoma |
| ILMN_2705689 | 74105 | Gga2 | 1.173 | 9.079 | 1.61E-08 | adenocarcinoma |
| ILMN_2624385 | 67023 | Use1 | -1.188 | 10.942 | 1.61E-08 | adenocarcinoma |
| ILMN_2659168 | 56506 | Cib2 | 1.247 | 8.628 | 1.61E-08 | adenocarcinoma |
| ILMN_2762326 | 110033 | Kif22 | 1.953 | 9.685 | 1.61E-08 | adenocarcinoma |
| ILMN_1226688 | 13850 | Ephx2 | -1.193 | 8.816 | 1.62E-08 | adenocarcinoma |
| ILMN_2427307 | 544963 | Iqgap2 | -1.035 | 9.064 | 1.64E-08 | adenocarcinoma |
| ILMN_2546272 | 14701 | Gng12 | -1.122 | 11.051 | 1.65E-08 | adenocarcinoma |
| ILMN_1224997 | 17119 | Mxd1 | -1.371 | 9.355 | 1.65E-08 | adenocarcinoma |
| ILMN_2665715 | 103140 | Gstt3 | -1.190 | 10.996 | 1.67E-08 | adenocarcinoma |
| ILMN_2645341 | 101772 | Ano1 | -1.173 | 9.503 | 1.69E-08 | adenocarcinoma |
| ILMN_2707043 | 13595 | Ebp | -1.238 | 11.147 | 1.73E-08 | adenocarcinoma |
| ILMN_2715802 | NA | NA | 1.410 | 10.747 | 1.75E-08 | adenocarcinoma |
| ILMN_2943523 | 212989 | Best2 | -1.240 | 10.429 | 1.76E-08 | adenocarcinoma |
| ILMN_2492170 | NA | NA | -1.363 | 11.292 | 1.76E-08 | adenocarcinoma |
| ILMN_2705849 | 26378 | Decr2 | -1.011 | 8.995 | 1.76E-08 | adenocarcinoma |
| ILMN_1236868 | NA | NA | 1.457 | 9.039 | 1.77E-08 | adenocarcinoma |
| ILMN_2649068 | 16362 | Irf1 | -1.340 | 10.009 | 1.77E-08 | adenocarcinoma |
| ILMN_1241279 | 100503565 | Gm9926 | -1.784 | 11.057 | 1.77E-08 | adenocarcinoma |
| ILMN_1246558 | 217138 | Prr15l | -1.008 | 13.374 | 1.78E-08 | adenocarcinoma |
| ILMN_1237644 | 18438 | P2rx4 | -1.178 | 11.292 | 1.79E-08 | adenocarcinoma |
| ILMN_3048492 | 27416 | Abcc5 | 1.024 | 8.662 | 1.80E-08 | adenocarcinoma |
| ILMN_1215120 | 20351 | Sema4a | -1.321 | 13.771 | 1.81E-08 | adenocarcinoma |
| ILMN_1236477 | 19713 | Ret | 1.688 | 8.515 | 1.86E-08 | adenocarcinoma |
| ILMN_2667463 | 77090 | Ocel1 | -1.068 | 9.891 | 1.87E-08 | adenocarcinoma |
| ILMN_1221011 | 67306 | Zc2hc1a | -1.028 | 10.042 | 1.88E-08 | adenocarcinoma |
| ILMN_2847269 | 67808 | Tprgl | -1.075 | 12.059 | 1.88E-08 | adenocarcinoma |
| ILMN_2940195 | 56615 | Mgst1 | -1.420 | 12.146 | 1.88E-08 | adenocarcinoma |
| ILMN_2758878 | 67887 | Tmem66 | -1.164 | 10.259 | 1.89E-08 | adenocarcinoma |
| ILMN_2774267 | 76192 | Abhd12 | 1.121 | 9.197 | 1.90E-08 | adenocarcinoma |
| ILMN_2423789 | 56459 | Sae1 | 1.013 | 12.542 | 1.90E-08 | adenocarcinoma |
| ILMN_2755803 | 230584 | Yipf1 | -1.186 | 10.756 | 1.91E-08 | adenocarcinoma |
| ILMN_1225994 | NA | NA | 1.079 | 8.737 | 1.92E-08 | adenocarcinoma |
| ILMN_3158919 | 18762 | Prkcz | -1.346 | 10.919 | 1.93E-08 | adenocarcinoma |
| ILMN_2938390 | 69718 | Ipmk | -1.438 | 11.299 | 1.96E-08 | adenocarcinoma |
| ILMN_2641467 | 21366 | Slc6a6 | 1.245 | 11.921 | 1.97E-08 | adenocarcinoma |
| ILMN_1240164 | 14570 | Arhgdig | 1.008 | 7.971 | 2.03E-08 | adenocarcinoma |
| ILMN_2717549 | 16898 | Rps2 | 1.014 | 15.104 | 2.04E-08 | adenocarcinoma |
| ILMN_2711075 | 17395 | Mmp9 | 2.137 | 8.801 | 2.06E-08 | adenocarcinoma |
| ILMN_2999439 | 16600 | Klf4 | -1.365 | 10.221 | 2.09E-08 | adenocarcinoma |
| ILMN_2622605 | 27364 | Srr | -1.310 | 9.632 | 2.11E-08 | adenocarcinoma |
| ILMN_2703585 | 14467 | Gbas | -1.020 | 10.455 | 2.11E-08 | adenocarcinoma |
| ILMN_1232766 | 109232 | Sccpdh | 1.095 | 10.123 | 2.11E-08 | adenocarcinoma |
| ILMN_2942276 | 269593 | Luzp1 | -1.180 | 12.562 | 2.12E-08 | adenocarcinoma |
| ILMN_2600720 | 76429 | Lhpp | 1.176 | 9.125 | 2.14E-08 | adenocarcinoma |
| ILMN_2651526 | 52864 | Slx4 | 1.055 | 9.883 | 2.15E-08 | adenocarcinoma |
| ILMN_3065373 | 223626 | Them6 | 1.042 | 8.965 | 2.16E-08 | adenocarcinoma |
| ILMN_2986315 | 56209 | Gde1 | -1.042 | 13.303 | 2.17E-08 | adenocarcinoma |
| ILMN_2544343 | 70021 | Nt5dc2 | 1.418 | 8.465 | 2.18E-08 | adenocarcinoma |
| ILMN_2454204 | NA | NA | 1.732 | 8.481 | 2.22E-08 | adenocarcinoma |
| ILMN_2638721 | 207785 | Csrnp2 | 1.136 | 9.825 | 2.24E-08 | adenocarcinoma |
| ILMN_2751046 | 18674 | Slc25a3 | -1.145 | 12.432 | 2.25E-08 | adenocarcinoma |
| ILMN_2612206 | 107995 | Cdc20 | 1.764 | 8.619 | 2.26E-08 | adenocarcinoma |
| ILMN_2971845 | 21915 | Dtymk | 1.020 | 10.082 | 2.27E-08 | adenocarcinoma |
| ILMN_1230799 | 117198 | Ivns1abp | -1.250 | 13.288 | 2.27E-08 | adenocarcinoma |
| ILMN_1241952 | NA | NA | 1.197 | 8.843 | 2.27E-08 | adenocarcinoma |
| ILMN_1234292 | 20463 | Cox7a2l | -1.128 | 10.750 | 2.30E-08 | adenocarcinoma |
| ILMN_2591425 | 60596 | Gucy1a3 | -1.146 | 9.310 | 2.32E-08 | adenocarcinoma |
| ILMN_2719687 | 70101 | Cyp4f16 | -1.557 | 9.724 | 2.34E-08 | adenocarcinoma |
| ILMN_2613641 | 13682 | Eif4a2 | -1.536 | 12.592 | 2.35E-08 | adenocarcinoma |
| ILMN_2616709 | 64652 | Nisch | 1.294 | 12.649 | 2.36E-08 | adenocarcinoma |
| ILMN_3009908 | 67920 | Mak16 | 1.068 | 11.557 | 2.37E-08 | adenocarcinoma |
| ILMN_2546510 | 434402 | Gm5617 | -1.122 | 11.753 | 2.38E-08 | adenocarcinoma |
| ILMN_1238838 | 80904 | Dtx3 | 1.371 | 9.118 | 2.39E-08 | adenocarcinoma |
| ILMN_2551741 | NA | NA | -1.521 | 12.006 | 2.41E-08 | adenocarcinoma |
| ILMN_1245858 | 269587 | Epb4.1 | 1.037 | 12.579 | 2.41E-08 | adenocarcinoma |
| ILMN_1228093 | 73167 | Arhgap8 | 1.263 | 9.536 | 2.41E-08 | adenocarcinoma |
| ILMN_2611728 | 67996 | Srsf6 | 1.594 | 10.503 | 2.42E-08 | adenocarcinoma |
| ILMN_2648695 | 66609 | Cryzl1 | -1.323 | 10.788 | 2.46E-08 | adenocarcinoma |
| ILMN_2595732 | 18030 | Nfil3 | 1.588 | 9.416 | 2.47E-08 | adenocarcinoma |
| ILMN_2632712 | 11799 | Birc5 | 1.955 | 9.139 | 2.47E-08 | adenocarcinoma |
| ILMN_2919860 | 74596 | Cds1 | -1.484 | 11.732 | 2.49E-08 | adenocarcinoma |
| ILMN_1255021 | 13240 | Defa6 | 1.602 | 8.351 | 2.52E-08 | adenocarcinoma |
| ILMN_1246173 | 17701 | Msx1 | 1.631 | 8.459 | 2.54E-08 | adenocarcinoma |
| ILMN_2621385 | 11545 | Parp1 | 1.480 | 9.861 | 2.55E-08 | adenocarcinoma |
| ILMN_2605890 | 21877 | Tk1 | 1.455 | 8.721 | 2.60E-08 | adenocarcinoma |
| ILMN_2819112 | NA | NA | 1.687 | 8.469 | 2.60E-08 | adenocarcinoma |
| ILMN_2429108 | NA | NA | 1.308 | 9.567 | 2.63E-08 | adenocarcinoma |
| ILMN_1214459 | 53617 | Krt35 | 1.284 | 8.229 | 2.70E-08 | adenocarcinoma |
| ILMN_2670398 | 13685 | Eif4ebp1 | 1.003 | 10.599 | 2.71E-08 | adenocarcinoma |
| ILMN_1234929 | NA | NA | 1.132 | 9.574 | 2.71E-08 | adenocarcinoma |
| ILMN_2630641 | 20810 | Srm | 1.029 | 8.804 | 2.71E-08 | adenocarcinoma |
| ILMN_2424268 | 101543 | Wtip | 1.117 | 10.391 | 2.74E-08 | adenocarcinoma |
| ILMN_1247074 | NA | NA | 1.273 | 8.854 | 2.74E-08 | adenocarcinoma |
| ILMN_3130719 | 319613 | Sybu | -1.257 | 9.680 | 2.79E-08 | adenocarcinoma |
| ILMN_1260450 | 13195 | Ddc | -1.283 | 10.533 | 2.81E-08 | adenocarcinoma |
| ILMN_2964324 | 16011 | Igfbp5 | 1.801 | 11.070 | 2.85E-08 | adenocarcinoma |
| ILMN_2701501 | 52683 | Ncaph2 | 1.072 | 9.529 | 2.85E-08 | adenocarcinoma |
| ILMN_2739965 | 104318 | Csnk1d | 1.566 | 11.204 | 2.86E-08 | adenocarcinoma |
| ILMN_1254902 | 103142 | Rdh9 | 1.788 | 10.562 | 2.86E-08 | adenocarcinoma |
| ILMN_2713285 | 14199 | Fhl1 | -2.049 | 14.329 | 2.87E-08 | adenocarcinoma |
| ILMN_2750402 | 110959 | Nudt19 | -1.123 | 10.419 | 2.89E-08 | adenocarcinoma |
| ILMN_1249480 | 219189 | Vwa8 | -1.070 | 9.775 | 2.91E-08 | adenocarcinoma |
| ILMN_2914843 | 19171 | Psmb10 | -1.312 | 12.907 | 2.95E-08 | adenocarcinoma |
| ILMN_1258691 | 170761 | Pdzd3 | -1.337 | 9.223 | 2.96E-08 | adenocarcinoma |
| ILMN_1225932 | NA | NA | 1.029 | 8.282 | 3.09E-08 | adenocarcinoma |
| ILMN_1235819 | 19384 | Ran | 1.114 | 13.170 | 3.13E-08 | adenocarcinoma |
| ILMN_1220612 | 230822 | Ncmap | 1.235 | 8.302 | 3.14E-08 | adenocarcinoma |
| ILMN_2672778 | 57742 | Abhd1 | -1.206 | 9.554 | 3.15E-08 | adenocarcinoma |
| ILMN_1216689 | 11804 | Aplp2 | 1.075 | 13.922 | 3.18E-08 | adenocarcinoma |
| ILMN_1231035 | 104318 | Csnk1d | 1.637 | 11.330 | 3.20E-08 | adenocarcinoma |
| ILMN_2633386 | NA | NA | -1.431 | 11.829 | 3.21E-08 | adenocarcinoma |
| ILMN_1215409 | 70274 | Ly6g6e | -1.296 | 10.667 | 3.23E-08 | adenocarcinoma |
| ILMN_1213448 | NA | NA | 1.479 | 8.898 | 3.30E-08 | adenocarcinoma |
| ILMN_1214036 | 217995 | Heatr1 | 1.223 | 11.423 | 3.30E-08 | adenocarcinoma |
| ILMN_2650603 | 54352 | Irx5 | 1.115 | 8.110 | 3.32E-08 | adenocarcinoma |
| ILMN_2625601 | 56018 | Stard10 | -1.289 | 11.042 | 3.33E-08 | adenocarcinoma |
| ILMN_2701271 | 22038 | Plscr1 | -1.172 | 12.162 | 3.33E-08 | adenocarcinoma |
| ILMN_2680329 | 69046 | Isca1 | -1.044 | 9.916 | 3.40E-08 | adenocarcinoma |
| ILMN_1226755 | NA | NA | 1.318 | 9.070 | 3.45E-08 | adenocarcinoma |
| ILMN_2741985 | 19366 | Rad54l | 1.457 | 8.351 | 3.45E-08 | adenocarcinoma |
| ILMN_2645275 | 192156 | Mvd | 1.115 | 9.250 | 3.46E-08 | adenocarcinoma |
| ILMN_2770609 | NA | NA | 1.199 | 8.201 | 3.49E-08 | adenocarcinoma |
| ILMN_2749958 | 223753 | Cerk | 1.225 | 10.834 | 3.55E-08 | adenocarcinoma |
| ILMN_2662602 | 107589 | Mylk | -1.327 | 11.469 | 3.58E-08 | adenocarcinoma |
| ILMN_2448651 | 11717 | Ampd3 | -1.332 | 11.046 | 3.60E-08 | adenocarcinoma |
| ILMN_2851671 | 50918 | Myadm | 1.227 | 10.845 | 3.61E-08 | adenocarcinoma |
| ILMN_2972829 | 56868 | Psg23 | 1.508 | 10.625 | 3.62E-08 | adenocarcinoma |
| ILMN_2692733 | 320709 | Tmem117 | -1.290 | 9.810 | 3.63E-08 | adenocarcinoma |
| ILMN_1240857 | 12865 | Cox7a1 | -1.552 | 11.438 | 3.67E-08 | adenocarcinoma |
| ILMN_1235070 | 18175 | Nrap | -1.241 | 9.516 | 3.69E-08 | adenocarcinoma |
| ILMN_3102736 | 229599 | Ciart | -1.441 | 8.757 | 3.76E-08 | adenocarcinoma |
| ILMN_2598374 | 215707 | Ccdc92 | 1.131 | 8.868 | 3.81E-08 | adenocarcinoma |
| ILMN_2626389 | 211548 | Nomo1 | 1.158 | 10.264 | 3.81E-08 | adenocarcinoma |
| ILMN_1257623 | NA | NA | 1.389 | 8.417 | 3.89E-08 | adenocarcinoma |
| ILMN_2711267 | 16668 | Krt18 | 1.042 | 11.956 | 3.91E-08 | adenocarcinoma |
| ILMN_1233122 | 12095 | Bglap3 | 1.134 | 9.139 | 3.91E-08 | adenocarcinoma |
| ILMN_2817892 | 18972 | Pold2 | 1.158 | 9.372 | 3.91E-08 | adenocarcinoma |
| ILMN_1242566 | 13511 | Dsg2 | -1.094 | 10.874 | 3.92E-08 | adenocarcinoma |
| ILMN_2686252 | 327655 | Ppip5k1 | -1.109 | 10.102 | 3.93E-08 | adenocarcinoma |
| ILMN_2418483 | 215627 | Zbtb8b | 1.027 | 8.155 | 3.95E-08 | adenocarcinoma |
| ILMN_2609753 | 26565 | Pla2g10 | -1.143 | 13.145 | 3.98E-08 | adenocarcinoma |
| ILMN_1246664 | NA | NA | 1.004 | 8.163 | 4.00E-08 | adenocarcinoma |
| ILMN_3071741 | 72002 | Slc39a5 | -1.209 | 9.001 | 4.00E-08 | adenocarcinoma |
| ILMN_2591713 | 215335 | Slc36a1 | -1.043 | 8.809 | 4.02E-08 | adenocarcinoma |
| ILMN_1228913 | 243771 | Parp12 | -1.107 | 9.264 | 4.15E-08 | adenocarcinoma |
| ILMN_2876775 | 72562 | Pcbd2 | -1.065 | 11.412 | 4.18E-08 | adenocarcinoma |
| ILMN_1252202 | 21929 | Tnfaip3 | -1.034 | 9.541 | 4.26E-08 | adenocarcinoma |
| ILMN_2776857 | 56150 | Mad2l1 | 1.053 | 8.262 | 4.29E-08 | adenocarcinoma |
| ILMN_2706061 | 71514 | Sfpq | 1.053 | 10.852 | 4.30E-08 | adenocarcinoma |
| ILMN_2734693 | 106347 | Ildr1 | -1.042 | 10.188 | 4.36E-08 | adenocarcinoma |
| ILMN_2507810 | 100042862 | Gm4076 | -1.326 | 11.788 | 4.37E-08 | adenocarcinoma |
| ILMN_2944601 | NA | NA | 1.446 | 11.134 | 4.38E-08 | adenocarcinoma |
| ILMN_2653972 | 320394 | Cenpt | 1.011 | 8.975 | 4.46E-08 | adenocarcinoma |
| ILMN_3117381 | 14199 | Fhl1 | -2.152 | 14.292 | 4.47E-08 | adenocarcinoma |
| ILMN_2979307 | 23954 | Nek3 | -1.057 | 9.475 | 4.47E-08 | adenocarcinoma |
| ILMN_2606210 | 56429 | Dpt | -1.200 | 9.160 | 4.50E-08 | adenocarcinoma |
| ILMN_1235050 | 170718 | Idh3b | -1.129 | 11.648 | 4.50E-08 | adenocarcinoma |
| ILMN_1218967 | 73804 | Kif2c | 1.725 | 8.701 | 4.54E-08 | adenocarcinoma |
| ILMN_2627690 | 15388 | Hnrnpl | 1.094 | 12.204 | 4.54E-08 | adenocarcinoma |
| ILMN_2700168 | 12444 | Ccnd2 | 1.224 | 9.307 | 4.60E-08 | adenocarcinoma |
| ILMN_2929572 | 381175 | Ccdc68 | -1.375 | 11.482 | 4.61E-08 | adenocarcinoma |
| ILMN_2601519 | 76507 | Aoc1 | -1.188 | 13.556 | 4.62E-08 | adenocarcinoma |
| ILMN_2667190 | NA | NA | -1.671 | 13.609 | 4.63E-08 | adenocarcinoma |
| ILMN_2722455 | 27374 | Prmt5 | 1.131 | 10.053 | 4.74E-08 | adenocarcinoma |
| ILMN_2880346 | 72462 | Rrp1b | 1.271 | 8.425 | 4.89E-08 | adenocarcinoma |
| ILMN_2699126 | 72562 | Pcbd2 | -1.029 | 10.189 | 4.89E-08 | adenocarcinoma |
| ILMN_3119014 | 13713 | Elk3 | 1.103 | 9.545 | 4.97E-08 | adenocarcinoma |
| ILMN_2989955 | 12153 | Bmp1 | 1.079 | 9.955 | 5.01E-08 | adenocarcinoma |
| ILMN_2672297 | 14537 | Gcnt1 | -1.142 | 10.031 | 5.05E-08 | adenocarcinoma |
| ILMN_2868838 | 240675 | Vwa2 | 1.026 | 8.352 | 5.09E-08 | adenocarcinoma |
| ILMN_1228752 | 71780 | Isyna1 | 1.128 | 8.850 | 5.15E-08 | adenocarcinoma |
| ILMN_3051608 | 67171 | Dram2 | -1.173 | 11.773 | 5.18E-08 | adenocarcinoma |
| ILMN_1254218 | NA | NA | 1.698 | 10.395 | 5.18E-08 | adenocarcinoma |
| ILMN_1245272 | 231070 | Insig1 | -1.246 | 9.630 | 5.19E-08 | adenocarcinoma |
| ILMN_1225029 | 218121 | Mboat1 | -1.457 | 12.737 | 5.21E-08 | adenocarcinoma |
| ILMN_2747923 | 53945 | Slc40a1 | -2.061 | 14.374 | 5.32E-08 | adenocarcinoma |
| ILMN_1231573 | 66222 | Serpinb1a | -1.787 | 12.075 | 5.37E-08 | adenocarcinoma |
| ILMN_1247636 | 76273 | Ndfip2 | -1.092 | 9.580 | 5.38E-08 | adenocarcinoma |
| ILMN_1213273 | 320398 | Lrig3 | -1.355 | 11.363 | 5.45E-08 | adenocarcinoma |
| ILMN_2452717 | NA | NA | 1.257 | 10.693 | 5.47E-08 | adenocarcinoma |
| ILMN_1233402 | 229521 | Syt11 | 1.011 | 10.263 | 5.51E-08 | adenocarcinoma |
| ILMN_2916705 | 22142 | Tuba1a | 1.032 | 9.494 | 5.55E-08 | adenocarcinoma |
| ILMN_2593774 | 66214 | Rgcc | 2.002 | 10.108 | 5.61E-08 | adenocarcinoma |
| ILMN_2636004 | 320878 | Mical2 | -1.477 | 10.928 | 5.88E-08 | adenocarcinoma |
| ILMN_2855315 | 50708 | Hist1h1c | -1.106 | 14.073 | 5.88E-08 | adenocarcinoma |
| ILMN_1238479 | 66447 | Mgst3 | -1.460 | 13.429 | 5.90E-08 | adenocarcinoma |
| ILMN_2460555 | NA | NA | -1.241 | 10.041 | 5.90E-08 | adenocarcinoma |
| ILMN_1242912 | 69693 | Pof1b | -1.578 | 11.645 | 5.91E-08 | adenocarcinoma |
| ILMN_2606693 | 231070 | Insig1 | -1.238 | 9.150 | 5.95E-08 | adenocarcinoma |
| ILMN_1243926 | 226654 | Tstd1 | -1.733 | 11.154 | 5.99E-08 | adenocarcinoma |
| ILMN_2609998 | 59095 | Fxyd6 | 1.739 | 12.270 | 6.02E-08 | adenocarcinoma |
| ILMN_2600537 | 109552 | Sri | -1.026 | 12.352 | 6.08E-08 | adenocarcinoma |
| ILMN_2954881 | 27060 | Tcirg1 | 1.091 | 9.393 | 6.13E-08 | adenocarcinoma |
| ILMN_1225602 | 20193 | S100a1 | -1.358 | 11.371 | 6.22E-08 | adenocarcinoma |
| ILMN_2778122 | 18569 | Pdcd4 | -1.274 | 12.945 | 6.31E-08 | adenocarcinoma |
| ILMN_2656871 | 69071 | Tmem97 | 1.297 | 10.565 | 6.32E-08 | adenocarcinoma |
| ILMN_1236175 | 22153 | Tubb4a | 1.329 | 8.372 | 6.35E-08 | adenocarcinoma |
| ILMN_2757569 | 13808 | Eno3 | -2.063 | 12.938 | 6.44E-08 | adenocarcinoma |
| ILMN_2754551 | 13479 | Dpep1 | -1.654 | 9.466 | 6.45E-08 | adenocarcinoma |
| ILMN_1242333 | 93757 | Immp2l | -1.145 | 9.946 | 6.48E-08 | adenocarcinoma |
| ILMN_2713586 | 68977 | Haghl | 1.066 | 10.567 | 6.50E-08 | adenocarcinoma |
| ILMN_2834379 | 21810 | Tgfbi | 1.447 | 10.213 | 6.51E-08 | adenocarcinoma |
| ILMN_2667346 | 76650 | Srxn1 | -1.097 | 9.885 | 6.64E-08 | adenocarcinoma |
| ILMN_2682613 | 16011 | Igfbp5 | 2.006 | 12.269 | 6.65E-08 | adenocarcinoma |
| ILMN_2635395 | 14915 | Guca2a | -1.722 | 15.277 | 6.65E-08 | adenocarcinoma |
| ILMN_3161120 | NA | NA | -1.427 | 10.852 | 6.68E-08 | adenocarcinoma |
| ILMN_1222071 | NA | NA | 1.627 | 9.987 | 6.76E-08 | adenocarcinoma |
| ILMN_1230162 | 14155 | Fem1b | 1.079 | 10.574 | 6.77E-08 | adenocarcinoma |
| ILMN_2950343 | 242505 | Rasef | -1.247 | 9.670 | 6.86E-08 | adenocarcinoma |
| ILMN_1259076 | 245945 | Rbm47 | -1.031 | 9.402 | 6.86E-08 | adenocarcinoma |
| ILMN_2742867 | 66902 | Mtap | 1.100 | 9.073 | 7.09E-08 | adenocarcinoma |
| ILMN_1237695 | 56421 | Pfkp | -1.448 | 11.844 | 7.10E-08 | adenocarcinoma |
| ILMN_2840818 | 66588 | Cmpk1 | -1.286 | 13.191 | 7.20E-08 | adenocarcinoma |
| ILMN_1226754 | 54383 | Phc2 | 1.053 | 11.366 | 7.21E-08 | adenocarcinoma |
| ILMN_2790486 | 11950 | Atp5f1 | -1.250 | 12.860 | 7.34E-08 | adenocarcinoma |
| ILMN_2773537 | 12716 | Ckmt1 | -1.358 | 15.437 | 7.43E-08 | adenocarcinoma |
| ILMN_2977624 | 20133 | Rrm1 | 1.112 | 9.683 | 7.51E-08 | adenocarcinoma |
| ILMN_3161036 | 17229 | Tpsb2 | 1.396 | 8.248 | 7.53E-08 | adenocarcinoma |
| ILMN_2832524 | 76282 | Gpt | -1.327 | 9.919 | 7.68E-08 | adenocarcinoma |
| ILMN_1248211 | 320007 | Sidt1 | 1.002 | 9.641 | 7.78E-08 | adenocarcinoma |
| ILMN_1245509 | 93961 | B3galt5 | -1.459 | 11.675 | 7.85E-08 | adenocarcinoma |
| ILMN_2688631 | 12946 | Cr1l | -1.108 | 9.701 | 7.92E-08 | adenocarcinoma |
| ILMN_1260372 | 27355 | Pald1 | 1.154 | 8.636 | 8.02E-08 | adenocarcinoma |
| ILMN_1239055 | 22436 | Xdh | -1.096 | 10.151 | 8.04E-08 | adenocarcinoma |
| ILMN_2987564 | 74868 | Tmem65 | 1.012 | 10.859 | 8.10E-08 | adenocarcinoma |
| ILMN_2763194 | 52793 | Fam3b | -1.787 | 11.335 | 8.15E-08 | adenocarcinoma |
| ILMN_1233008 | 72831 | Dhx30 | 1.128 | 11.227 | 8.38E-08 | adenocarcinoma |
| ILMN_2704822 | 52538 | Acaa2 | -1.518 | 11.198 | 8.41E-08 | adenocarcinoma |
| ILMN_2481117 | 140481 | Man2a2 | 1.179 | 9.767 | 8.45E-08 | adenocarcinoma |
| ILMN_2472444 | 22032 | Traf4 | 1.316 | 10.266 | 8.54E-08 | adenocarcinoma |
| ILMN_2695181 | 29856 | Smtn | -1.175 | 10.986 | 8.66E-08 | adenocarcinoma |
| ILMN_2702215 | 13682 | Eif4a2 | -2.202 | 11.603 | 8.96E-08 | adenocarcinoma |
| ILMN_2684145 | 228765 | Sdcbp2 | -1.299 | 11.789 | 8.97E-08 | adenocarcinoma |
| ILMN_3127335 | 83671 | Sytl2 | -1.312 | 12.060 | 9.05E-08 | adenocarcinoma |
| ILMN_1248388 | 50770 | Atp11a | 1.153 | 9.322 | 9.07E-08 | adenocarcinoma |
| ILMN_1221501 | 70564 | Fam213a | 1.443 | 10.820 | 9.12E-08 | adenocarcinoma |
| ILMN_2991389 | 70274 | Ly6g6e | -1.405 | 12.136 | 9.16E-08 | adenocarcinoma |
| ILMN_2785679 | 66106 | Smpx | 1.194 | 8.696 | 9.21E-08 | adenocarcinoma |
| ILMN_2899863 | 21926 | Tnf | 1.025 | 8.186 | 9.24E-08 | adenocarcinoma |
| ILMN_2661214 | 16667 | Krt17 | 1.997 | 9.865 | 9.50E-08 | adenocarcinoma |
| ILMN_3052390 | 72190 | 2510009E07Rik | 1.074 | 11.728 | 9.55E-08 | adenocarcinoma |
| ILMN_2907655 | 12028 | Bax | 1.087 | 9.550 | 9.84E-08 | adenocarcinoma |
| ILMN_2424866 | 98417 | Cnih4 | -1.084 | 12.024 | 9.86E-08 | adenocarcinoma |
| ILMN_1256795 | 67204 | Eif2s2 | -1.159 | 11.797 | 9.95E-08 | adenocarcinoma |
| ILMN_2766930 | 14073 | Faah | 1.007 | 9.427 | 9.97E-08 | adenocarcinoma |
| ILMN_2709997 | 52793 | Fam3b | -1.488 | 10.114 | 1.00E-07 | adenocarcinoma |
| ILMN_2636266 | 233335 | Synm | -1.040 | 9.079 | 1.01E-07 | adenocarcinoma |
| ILMN_2501719 | 72088 | Ush1c | -1.111 | 12.931 | 1.01E-07 | adenocarcinoma |
| ILMN_1260512 | 17319 | Mif | 1.007 | 11.036 | 1.03E-07 | adenocarcinoma |
| ILMN_1251696 | 16363 | Irf2 | -1.019 | 12.631 | 1.04E-07 | adenocarcinoma |
| ILMN_1245424 | 231327 | Ppat | 1.041 | 9.012 | 1.05E-07 | adenocarcinoma |
| ILMN_2615035 | 66447 | Mgst3 | -1.236 | 15.213 | 1.06E-07 | adenocarcinoma |
| ILMN_2591414 | 52440 | Tax1bp1 | -1.212 | 12.391 | 1.09E-07 | adenocarcinoma |
| ILMN_2598239 | 59041 | Stk25 | -1.057 | 10.345 | 1.10E-07 | adenocarcinoma |
| ILMN_2628745 | 234076 | Tmco3 | -1.091 | 10.336 | 1.10E-07 | adenocarcinoma |
| ILMN_1231689 | 20377 | Sfrp1 | -1.997 | 9.786 | 1.11E-07 | adenocarcinoma |
| ILMN_2867901 | 107995 | Cdc20 | 1.503 | 8.635 | 1.13E-07 | adenocarcinoma |
| ILMN_2973024 | 68948 | Fam216a | 1.079 | 9.093 | 1.13E-07 | adenocarcinoma |
| ILMN_2681013 | 50935 | St6galnac6 | -1.124 | 9.621 | 1.14E-07 | adenocarcinoma |
| ILMN_2631161 | 246256 | Fcgr4 | 1.443 | 9.444 | 1.14E-07 | adenocarcinoma |
| ILMN_2669062 | 74116 | Pi16 | -1.420 | 9.113 | 1.15E-07 | adenocarcinoma |
| ILMN_2759058 | 22644 | Rnf103 | -1.039 | 13.253 | 1.15E-07 | adenocarcinoma |
| ILMN_1240160 | 56183 | Nmu | -1.245 | 8.828 | 1.16E-07 | adenocarcinoma |
| ILMN_1257667 | 27221 | Chaf1a | 1.079 | 8.451 | 1.17E-07 | adenocarcinoma |
| ILMN_1257332 | 59030 | Mkks | -1.244 | 10.546 | 1.17E-07 | adenocarcinoma |
| ILMN_2739760 | 116847 | Prelp | -2.213 | 11.158 | 1.18E-07 | adenocarcinoma |
| ILMN_1215552 | 108017 | Fxyd4 | -1.857 | 9.428 | 1.18E-07 | adenocarcinoma |
| ILMN_1226035 | 67231 | Tbc1d20 | -1.016 | 10.714 | 1.18E-07 | adenocarcinoma |
| ILMN_2757916 | 67211 | Armc10 | 1.086 | 10.670 | 1.20E-07 | adenocarcinoma |
| ILMN_1222725 | 240675 | Vwa2 | 1.131 | 8.176 | 1.21E-07 | adenocarcinoma |
| ILMN_2826709 | 108888 | Atad3a | 1.207 | 11.503 | 1.22E-07 | adenocarcinoma |
| ILMN_2952098 | 319317 | Snhg11 | 1.019 | 8.327 | 1.23E-07 | adenocarcinoma |
| ILMN_2667551 | 109075 | Exosc4 | 1.020 | 10.222 | 1.23E-07 | adenocarcinoma |
| ILMN_2985836 | 232748 | Fam115c | 1.027 | 9.781 | 1.23E-07 | adenocarcinoma |
| ILMN_2763825 | 69718 | Ipmk | -1.499 | 12.261 | 1.26E-07 | adenocarcinoma |
| ILMN_2763674 | 26383 | Fto | 1.102 | 11.636 | 1.26E-07 | adenocarcinoma |
| ILMN_1238627 | 319317 | Snhg11 | 1.114 | 8.303 | 1.30E-07 | adenocarcinoma |
| ILMN_2676127 | 224024 | Scarf2 | 1.198 | 10.371 | 1.30E-07 | adenocarcinoma |
| ILMN_2812926 | 226781 | Slc30a10 | -2.341 | 9.131 | 1.30E-07 | adenocarcinoma |
| ILMN_2472451 | 22032 | Traf4 | 1.310 | 9.922 | 1.31E-07 | adenocarcinoma |
| ILMN_2870443 | 14287 | Fpgs | 1.333 | 10.500 | 1.33E-07 | adenocarcinoma |
| ILMN_2704576 | 17392 | Mmp3 | 2.020 | 8.731 | 1.41E-07 | adenocarcinoma |
| ILMN_1249753 | NA | NA | 1.059 | 8.440 | 1.41E-07 | adenocarcinoma |
| ILMN_2543393 | 52276 | Cdca8 | 2.005 | 9.344 | 1.45E-07 | adenocarcinoma |
| ILMN_3086192 | 53422 | Ybx2 | -1.153 | 9.401 | 1.50E-07 | adenocarcinoma |
| ILMN_2730651 | 30962 | Slc7a9 | -1.485 | 9.292 | 1.51E-07 | adenocarcinoma |
| ILMN_2909238 | 20741 | Sptb | 1.220 | 8.563 | 1.52E-07 | adenocarcinoma |
| ILMN_1260073 | 67102 | D16Ertd472e | 1.213 | 9.011 | 1.53E-07 | adenocarcinoma |
| ILMN_2727153 | 12759 | Clu | 1.944 | 11.796 | 1.54E-07 | adenocarcinoma |
| ILMN_3145331 | 19240 | Tmsb10 | 1.510 | 10.974 | 1.56E-07 | adenocarcinoma |
| ILMN_2700550 | 320209 | Ddx11 | 1.063 | 8.696 | 1.61E-07 | adenocarcinoma |
| ILMN_2630852 | 16196 | Il7 | -1.297 | 9.767 | 1.62E-07 | adenocarcinoma |
| ILMN_1250913 | 226245 | Plekhs1 | 1.670 | 8.588 | 1.68E-07 | adenocarcinoma |
| ILMN_1254031 | 16601 | Klf9 | -1.246 | 11.960 | 1.68E-07 | adenocarcinoma |
| ILMN_2652500 | 76905 | Lrg1 | 2.536 | 9.318 | 1.70E-07 | adenocarcinoma |
| ILMN_2730003 | 108673 | Ccdc86 | 1.135 | 9.662 | 1.70E-07 | adenocarcinoma |
| ILMN_2515982 | NA | NA | -1.040 | 8.909 | 1.74E-07 | adenocarcinoma |
| ILMN_2607880 | 21881 | Tkt | 1.063 | 11.881 | 1.75E-07 | adenocarcinoma |
| ILMN_1217408 | 18749 | Prkacb | -1.007 | 13.674 | 1.75E-07 | adenocarcinoma |
| ILMN_2788629 | 98417 | Cnih4 | -1.052 | 12.068 | 1.77E-07 | adenocarcinoma |
| ILMN_2505970 | 74424 | Tmc5 | -1.088 | 10.549 | 1.78E-07 | adenocarcinoma |
| ILMN_2881019 | 58194 | Sh3kbp1 | 1.200 | 9.563 | 1.82E-07 | adenocarcinoma |
| ILMN_2686841 | 228765 | Sdcbp2 | -1.388 | 10.675 | 1.85E-07 | adenocarcinoma |
| ILMN_1230143 | 74481 | Batf2 | -1.089 | 9.495 | 1.86E-07 | adenocarcinoma |
| ILMN_2683630 | 67118 | Bfar | -1.208 | 11.356 | 1.90E-07 | adenocarcinoma |
| ILMN_1248318 | 20517 | Slc22a1 | 1.118 | 8.829 | 1.90E-07 | adenocarcinoma |
| ILMN_2753809 | 17392 | Mmp3 | 2.432 | 9.096 | 1.90E-07 | adenocarcinoma |
| ILMN_2614966 | 11891 | Rab27a | -1.462 | 12.124 | 1.94E-07 | adenocarcinoma |
| ILMN_2817864 | 76722 | Ckmt2 | -1.202 | 8.797 | 1.95E-07 | adenocarcinoma |
| ILMN_2681232 | 52668 | Ifi27 | -1.251 | 11.672 | 1.96E-07 | adenocarcinoma |
| ILMN_2867899 | 107995 | Cdc20 | 2.165 | 9.344 | 1.98E-07 | adenocarcinoma |
| ILMN_2735350 | 23886 | Gdf15 | 1.427 | 8.332 | 1.98E-07 | adenocarcinoma |
| ILMN_2548379 | NA | NA | -1.006 | 12.463 | 2.04E-07 | adenocarcinoma |
| ILMN_2751072 | 14672 | Gna11 | -1.104 | 11.450 | 2.07E-07 | adenocarcinoma |
| ILMN_2512297 | 68667 | Trpm4 | -1.158 | 9.216 | 2.08E-07 | adenocarcinoma |
| ILMN_3161878 | 12122 | Bid | 1.046 | 9.163 | 2.09E-07 | adenocarcinoma |
| ILMN_2929896 | 52033 | Pbk | 2.108 | 9.097 | 2.10E-07 | adenocarcinoma |
| ILMN_2778151 | 20682 | Sox9 | 1.572 | 12.799 | 2.16E-07 | adenocarcinoma |
| ILMN_2704826 | 52538 | Acaa2 | -1.371 | 10.845 | 2.17E-07 | adenocarcinoma |
| ILMN_1214731 | 15039 | H2-T22 | -1.348 | 10.877 | 2.17E-07 | adenocarcinoma |
| ILMN_2513781 | NA | NA | 1.388 | 8.323 | 2.18E-07 | adenocarcinoma |
| ILMN_1252730 | 71207 | Nudt4 | -1.033 | 12.392 | 2.18E-07 | adenocarcinoma |
| ILMN_1214408 | 667846 | Gm8841 | 1.021 | 13.892 | 2.22E-07 | adenocarcinoma |
| ILMN_1239863 | 214763 | Mb21d1 | 1.056 | 9.234 | 2.23E-07 | adenocarcinoma |
| ILMN_2416764 | 218865 | Chdh | -1.242 | 10.417 | 2.30E-07 | adenocarcinoma |
| ILMN_2974798 | 226519 | Lamc1 | 1.005 | 9.080 | 2.32E-07 | adenocarcinoma |
| ILMN_2941714 | 100647 | Upk3b | -1.734 | 9.048 | 2.33E-07 | adenocarcinoma |
| ILMN_1228374 | 65970 | Lima1 | -1.019 | 10.746 | 2.34E-07 | adenocarcinoma |
| ILMN_1232495 | 22350 | Ezr | -1.034 | 11.976 | 2.34E-07 | adenocarcinoma |
| ILMN_2718570 | NA | NA | -1.953 | 14.919 | 2.35E-07 | adenocarcinoma |
| ILMN_1236574 | 12615 | Cenpa | 2.243 | 10.367 | 2.35E-07 | adenocarcinoma |
| ILMN_2476139 | 22146 | Tuba1c | 1.246 | 11.814 | 2.35E-07 | adenocarcinoma |
| ILMN_1251301 | 74764 | Klc4 | -1.143 | 11.086 | 2.36E-07 | adenocarcinoma |
| ILMN_3122845 | 243529 | H1fx | 1.224 | 8.115 | 2.40E-07 | adenocarcinoma |
| ILMN_2632153 | 108912 | Cdca2 | 1.667 | 8.801 | 2.47E-07 | adenocarcinoma |
| ILMN_3083163 | 12870 | Cp | 1.311 | 8.653 | 2.53E-07 | adenocarcinoma |
| ILMN_2765759 | 65256 | Asb2 | -1.416 | 9.993 | 2.55E-07 | adenocarcinoma |
| ILMN_1220121 | 71819 | Kif23 | 1.657 | 9.082 | 2.55E-07 | adenocarcinoma |
| ILMN_2681805 | 17063 | Muc13 | -1.227 | 14.145 | 2.55E-07 | adenocarcinoma |
| ILMN_1217102 | 252972 | Tpcn1 | -1.017 | 11.515 | 2.55E-07 | adenocarcinoma |
| ILMN_2625893 | 104158 | Ces1d | -2.341 | 11.210 | 2.55E-07 | adenocarcinoma |
| ILMN_2670751 | 108017 | Fxyd4 | -2.090 | 10.232 | 2.58E-07 | adenocarcinoma |
| ILMN_1246573 | 75600 | Calml4 | -1.178 | 10.850 | 2.60E-07 | adenocarcinoma |
| ILMN_2661299 | 18858 | Pmp22 | -2.154 | 12.415 | 2.61E-07 | adenocarcinoma |
| ILMN_2744217 | 54141 | Spag5 | 1.060 | 8.451 | 2.61E-07 | adenocarcinoma |
| ILMN_2429025 | 22381 | Wbp5 | 1.065 | 11.986 | 2.61E-07 | adenocarcinoma |
| ILMN_1254015 | NA | NA | -1.473 | 13.114 | 2.63E-07 | adenocarcinoma |
| ILMN_2718217 | 67951 | Tubb6 | 1.270 | 9.582 | 2.63E-07 | adenocarcinoma |
| ILMN_2595973 | 14824 | Grn | 1.020 | 11.994 | 2.68E-07 | adenocarcinoma |
| ILMN_1230893 | 223254 | Farp1 | 1.010 | 10.614 | 2.68E-07 | adenocarcinoma |
| ILMN_2476452 | 70574 | Cpm | -1.206 | 10.066 | 2.69E-07 | adenocarcinoma |
| ILMN_2757125 | 233406 | Prc1 | 2.182 | 9.733 | 2.77E-07 | adenocarcinoma |
| ILMN_2604556 | 18583 | Pde7a | -1.008 | 9.160 | 2.77E-07 | adenocarcinoma |
| ILMN_3061300 | NA | NA | -1.279 | 9.447 | 2.81E-07 | adenocarcinoma |
| ILMN_1216213 | 16319 | Incenp | 1.203 | 8.441 | 2.86E-07 | adenocarcinoma |
| ILMN_2615380 | 104816 | Aspg | -1.005 | 8.805 | 2.89E-07 | adenocarcinoma |
| ILMN_1214671 | 71963 | Cdca4 | 1.126 | 11.059 | 2.90E-07 | adenocarcinoma |
| ILMN_3002745 | 27973 | Vkorc1 | -1.153 | 10.726 | 2.96E-07 | adenocarcinoma |
| ILMN_1258347 | NA | NA | -1.021 | 10.057 | 2.98E-07 | adenocarcinoma |
| ILMN_2680601 | 231293 | Cwh43 | 1.483 | 9.476 | 3.01E-07 | adenocarcinoma |
| ILMN_1249817 | 11950 | Atp5f1 | -1.148 | 11.956 | 3.02E-07 | adenocarcinoma |
| ILMN_1228942 | 12509 | Cd59a | -1.601 | 11.234 | 3.09E-07 | adenocarcinoma |
| ILMN_1227240 | 12579 | Cdkn2b | -1.051 | 11.835 | 3.10E-07 | adenocarcinoma |
| ILMN_2826110 | 12359 | Cat | -1.031 | 9.747 | 3.16E-07 | adenocarcinoma |
| ILMN_2909105 | NA | NA | 1.000 | 9.872 | 3.18E-07 | adenocarcinoma |
| ILMN_2621643 | 12826 | Col4a1 | 1.331 | 11.332 | 3.20E-07 | adenocarcinoma |
| ILMN_2628178 | 216233 | Socs2 | -1.125 | 10.906 | 3.21E-07 | adenocarcinoma |
| ILMN_2955973 | 29856 | Smtn | -1.013 | 10.434 | 3.28E-07 | adenocarcinoma |
| ILMN_2678547 | 18777 | Lypla1 | -1.101 | 10.836 | 3.29E-07 | adenocarcinoma |
| ILMN_2601884 | 74182 | Gpcpd1 | 1.106 | 10.142 | 3.35E-07 | adenocarcinoma |
| ILMN_2635232 | 19347 | Dennd5a | 1.080 | 9.681 | 3.40E-07 | adenocarcinoma |
| ILMN_2774007 | 12236 | Bub1b | 1.096 | 8.474 | 3.41E-07 | adenocarcinoma |
| ILMN_2589039 | 12864 | Cox6c | -1.250 | 12.578 | 3.45E-07 | adenocarcinoma |
| ILMN_2623776 | 13860 | Eps8 | -1.084 | 11.111 | 3.48E-07 | adenocarcinoma |
| ILMN_2840533 | 230163 | Aldob | -1.868 | 9.493 | 3.49E-07 | adenocarcinoma |
| ILMN_2466845 | 22059 | Trp53 | 1.185 | 9.135 | 3.56E-07 | adenocarcinoma |
| ILMN_3141801 | 192201 | Wfdc15b | 1.123 | 8.190 | 3.68E-07 | adenocarcinoma |
| ILMN_2654276 | 71843 | R3hcc1 | 1.022 | 9.472 | 3.69E-07 | adenocarcinoma |
| ILMN_2695819 | 73284 | Ddit4l | 1.045 | 8.534 | 3.72E-07 | adenocarcinoma |
| ILMN_2923463 | 71819 | Kif23 | 1.276 | 8.705 | 3.74E-07 | adenocarcinoma |
| ILMN_2698052 | 76722 | Ckmt2 | -1.113 | 8.975 | 3.76E-07 | adenocarcinoma |
| ILMN_1236029 | 217169 | Tns4 | -1.099 | 12.839 | 3.76E-07 | adenocarcinoma |
| ILMN_2746283 | 17540 | Mrvi1 | -1.146 | 9.719 | 3.79E-07 | adenocarcinoma |
| ILMN_2625920 | 11754 | Aoc3 | -1.326 | 9.869 | 3.81E-07 | adenocarcinoma |
| ILMN_2435302 | 24017 | Rnf13 | -1.097 | 9.859 | 3.82E-07 | adenocarcinoma |
| ILMN_2847276 | 17207 | Mcf2l | -1.087 | 10.224 | 3.83E-07 | adenocarcinoma |
| ILMN_1229197 | 667373 | Gm14446 | -2.183 | 11.197 | 3.92E-07 | adenocarcinoma |
| ILMN_1227001 | 19205 | Ptbp1 | 1.202 | 10.940 | 3.93E-07 | adenocarcinoma |
| ILMN_1214944 | 230806 | Aim1l | -1.250 | 11.159 | 4.06E-07 | adenocarcinoma |
| ILMN_1228948 | 394436 | Ugt1a1 | -1.211 | 8.454 | 4.10E-07 | adenocarcinoma |
| ILMN_2423282 | 668253 | Dleu2 | -1.007 | 9.112 | 4.11E-07 | adenocarcinoma |
| ILMN_1258688 | 494448 | Cbx6 | 1.080 | 8.854 | 4.17E-07 | adenocarcinoma |
| ILMN_1238360 | 15331 | Hmgn2 | 1.166 | 11.747 | 4.17E-07 | adenocarcinoma |
| ILMN_3150519 | 72002 | Slc39a5 | -1.233 | 9.349 | 4.19E-07 | adenocarcinoma |
| ILMN_2970023 | 70129 | Slc44a4 | -1.255 | 12.016 | 4.21E-07 | adenocarcinoma |
| ILMN_2822579 | 12827 | Col4a2 | 1.280 | 10.076 | 4.27E-07 | adenocarcinoma |
| ILMN_2815752 | 30962 | Slc7a9 | -1.341 | 9.196 | 4.31E-07 | adenocarcinoma |
| ILMN_1240256 | 26941 | Slc9a3r1 | -1.327 | 12.218 | 4.34E-07 | adenocarcinoma |
| ILMN_1228613 | 66194 | Pycrl | -1.085 | 10.414 | 4.38E-07 | adenocarcinoma |
| ILMN_1224012 | 230163 | Aldob | -1.399 | 9.230 | 4.46E-07 | adenocarcinoma |
| ILMN_2839313 | 11468 | Actg2 | -2.759 | 12.963 | 4.49E-07 | adenocarcinoma |
| ILMN_1224237 | NA | NA | 1.163 | 8.141 | 4.63E-07 | adenocarcinoma |
| ILMN_2694339 | 15433 | Hoxd13 | -1.446 | 9.498 | 4.66E-07 | adenocarcinoma |
| ILMN_2523316 | 107449 | Unc5b | -1.135 | 9.788 | 4.67E-07 | adenocarcinoma |
| ILMN_2449120 | 52398 | Sep-11 | 1.038 | 11.745 | 4.75E-07 | adenocarcinoma |
| ILMN_3089725 | 13052 | Cxadr | -1.202 | 11.307 | 4.76E-07 | adenocarcinoma |
| ILMN_2958076 | 66117 | 1110001J03Rik | -1.160 | 12.119 | 5.00E-07 | adenocarcinoma |
| ILMN_2879102 | 14620 | Gjb3 | -1.415 | 11.108 | 5.02E-07 | adenocarcinoma |
| ILMN_2536518 | 67972 | Atp2b1 | -1.017 | 10.688 | 5.08E-07 | adenocarcinoma |
| ILMN_2933112 | 18242 | Oat | -1.144 | 13.516 | 5.10E-07 | adenocarcinoma |
| ILMN_1235494 | 66241 | Tmem9 | 1.009 | 9.738 | 5.19E-07 | adenocarcinoma |
| ILMN_2735276 | 231999 | Plekha8 | -1.119 | 11.165 | 5.25E-07 | adenocarcinoma |
| ILMN_1214866 | 68519 | Eml1 | 1.322 | 10.562 | 5.26E-07 | adenocarcinoma |
| ILMN_2643513 | 27053 | Asns | 1.120 | 10.675 | 5.37E-07 | adenocarcinoma |
| ILMN_2666070 | 21337 | Tacr2 | -1.017 | 8.938 | 5.39E-07 | adenocarcinoma |
| ILMN_2425569 | 22635 | Zan | -1.396 | 9.386 | 5.45E-07 | adenocarcinoma |
| ILMN_1234319 | 17151 | Ccndbp1 | -1.108 | 10.792 | 5.46E-07 | adenocarcinoma |
| ILMN_2704823 | 52538 | Acaa2 | -1.318 | 10.780 | 5.50E-07 | adenocarcinoma |
| ILMN_2703748 | 66978 | Luc7l | 1.278 | 10.394 | 5.63E-07 | adenocarcinoma |
| ILMN_2790097 | 11853 | Rhoc | -1.281 | 11.873 | 5.68E-07 | adenocarcinoma |
| ILMN_2892507 | 14345 | Fut4 | -1.438 | 10.992 | 5.69E-07 | adenocarcinoma |
| ILMN_3029489 | 229599 | Ciart | -1.416 | 8.929 | 5.86E-07 | adenocarcinoma |
| ILMN_2449985 | 69878 | Snrpf | 1.008 | 11.022 | 6.20E-07 | adenocarcinoma |
| ILMN_2617228 | 20878 | Aurka | 1.375 | 8.993 | 6.23E-07 | adenocarcinoma |
| ILMN_2834777 | 16362 | Irf1 | -1.169 | 13.446 | 6.26E-07 | adenocarcinoma |
| ILMN_2639972 | 244416 | Ppp1r3b | -1.395 | 10.985 | 6.28E-07 | adenocarcinoma |
| ILMN_2631948 | 268756 | Gulo | 1.557 | 8.220 | 6.29E-07 | adenocarcinoma |
| ILMN_2758198 | 20322 | Sord | -1.005 | 9.342 | 6.63E-07 | adenocarcinoma |
| ILMN_1214573 | NA | NA | 1.023 | 8.223 | 6.82E-07 | adenocarcinoma |
| ILMN_1244460 | 13636 | Efna1 | -1.371 | 11.300 | 7.07E-07 | adenocarcinoma |
| ILMN_2952275 | 11846 | Arg1 | 2.776 | 8.589 | 7.10E-07 | adenocarcinoma |
| ILMN_2795412 | NA | NA | 1.626 | 9.938 | 7.14E-07 | adenocarcinoma |
| ILMN_2672528 | 54196 | Pabpn1 | 1.129 | 12.228 | 7.26E-07 | adenocarcinoma |
| ILMN_2657844 | 12534 | Cdk1 | 1.155 | 8.926 | 7.29E-07 | adenocarcinoma |
| ILMN_2644632 | 20910 | Stxbp1 | 1.084 | 11.776 | 7.66E-07 | adenocarcinoma |
| ILMN_2879008 | 13079 | Cyp21a1 | 1.092 | 8.118 | 7.75E-07 | adenocarcinoma |
| ILMN_2522693 | 27973 | Vkorc1 | -1.173 | 11.300 | 8.00E-07 | adenocarcinoma |
| ILMN_1214902 | 18458 | Pabpc1 | 1.267 | 10.139 | 8.15E-07 | adenocarcinoma |
| ILMN_1259202 | 17829 | Muc1 | -1.007 | 8.745 | 8.23E-07 | adenocarcinoma |
| ILMN_2743139 | NA | NA | -1.044 | 10.165 | 8.28E-07 | adenocarcinoma |
| ILMN_2645321 | 20672 | Sox18 | 1.228 | 8.536 | 8.97E-07 | adenocarcinoma |
| ILMN_2618714 | 18591 | Pdgfb | 1.237 | 8.554 | 9.00E-07 | adenocarcinoma |
| ILMN_3144164 | 384783 | Irs2 | -1.480 | 11.054 | 9.08E-07 | adenocarcinoma |
| ILMN_2493175 | 22117 | Tst | -1.306 | 11.956 | 9.27E-07 | adenocarcinoma |
| ILMN_2646625 | 16476 | Jun | 1.053 | 10.229 | 9.34E-07 | adenocarcinoma |
| ILMN_2736867 | 17110 | Lyz1 | 1.880 | 9.065 | 9.38E-07 | adenocarcinoma |
| ILMN_2907878 | 15270 | H2afx | 1.163 | 9.265 | 9.41E-07 | adenocarcinoma |
| ILMN_2694998 | 68953 | Chmp2a | -1.087 | 11.538 | 9.43E-07 | adenocarcinoma |
| ILMN_2497999 | 27973 | Vkorc1 | -1.026 | 10.180 | 9.44E-07 | adenocarcinoma |
| ILMN_3067068 | 19240 | Tmsb10 | 1.221 | 12.781 | 9.54E-07 | adenocarcinoma |
| ILMN_3133352 | 22256 | Ung | 1.065 | 8.768 | 9.81E-07 | adenocarcinoma |
| ILMN_2965641 | 668661 | 2410002F23Rik | 1.317 | 9.822 | 1.01E-06 | adenocarcinoma |
| ILMN_1250338 | 230259 | E130308A19Rik | 1.273 | 9.662 | 1.01E-06 | adenocarcinoma |
| ILMN_1249336 | 17386 | Mmp13 | 1.156 | 8.218 | 1.03E-06 | adenocarcinoma |
| ILMN_1229210 | 70466 | Ckap2l | 1.450 | 8.888 | 1.05E-06 | adenocarcinoma |
| ILMN_2900431 | 19885 | Rorc | -1.029 | 9.161 | 1.06E-06 | adenocarcinoma |
| ILMN_1258158 | 104776 | Aldh6a1 | -1.062 | 9.105 | 1.09E-06 | adenocarcinoma |
| ILMN_2941712 | 100647 | Upk3b | -1.442 | 8.634 | 1.10E-06 | adenocarcinoma |
| ILMN_1214290 | NA | NA | 1.519 | 8.762 | 1.10E-06 | adenocarcinoma |
| ILMN_2749556 | 72040 | Cdhr5 | -1.180 | 14.886 | 1.11E-06 | adenocarcinoma |
| ILMN_2669361 | 75578 | Fggy | 1.005 | 8.656 | 1.13E-06 | adenocarcinoma |
| ILMN_1247947 | 59010 | Sqrdl | -1.441 | 10.372 | 1.15E-06 | adenocarcinoma |
| ILMN_3040543 | 331063 | Gsdmc2 | -1.929 | 11.314 | 1.20E-06 | adenocarcinoma |
| ILMN_1257107 | 100503536 | Gm19757 | 2.443 | 13.498 | 1.22E-06 | adenocarcinoma |
| ILMN_2497616 | 67530 | Uqcrb | -1.041 | 10.248 | 1.22E-06 | adenocarcinoma |
| ILMN_2777498 | 16176 | Il1b | 1.779 | 8.650 | 1.23E-06 | adenocarcinoma |
| ILMN_2789904 | 67470 | Abcg8 | -1.835 | 10.283 | 1.25E-06 | adenocarcinoma |
| ILMN_2618148 | 77619 | Prelid2 | -1.192 | 11.433 | 1.28E-06 | adenocarcinoma |
| ILMN_1256676 | 69219 | Ddah1 | 2.138 | 9.884 | 1.28E-06 | adenocarcinoma |
| ILMN_1237886 | 13803 | Enc1 | 1.160 | 9.416 | 1.29E-06 | adenocarcinoma |
| ILMN_3157568 | 12125 | Bcl2l11 | 1.226 | 10.558 | 1.32E-06 | adenocarcinoma |
| ILMN_1225730 | 110196 | Fdps | 1.383 | 11.400 | 1.33E-06 | adenocarcinoma |
| ILMN_1253317 | 170750 | Xpnpep1 | 1.009 | 13.174 | 1.37E-06 | adenocarcinoma |
| ILMN_2718266 | 14229 | Fkbp5 | -1.100 | 9.818 | 1.39E-06 | adenocarcinoma |
| ILMN_1258526 | 19039 | Lgals3bp | 1.416 | 11.837 | 1.40E-06 | adenocarcinoma |
| ILMN_1219017 | 72349 | Dusp3 | -1.030 | 11.298 | 1.41E-06 | adenocarcinoma |
| ILMN_2480140 | NA | NA | 1.431 | 8.474 | 1.44E-06 | adenocarcinoma |
| ILMN_2701664 | 14605 | Tsc22d3 | -1.078 | 9.781 | 1.47E-06 | adenocarcinoma |
| ILMN_2489564 | NA | NA | 1.018 | 8.160 | 1.47E-06 | adenocarcinoma |
| ILMN_1220846 | 240514 | Ccdc85b | 1.035 | 10.246 | 1.48E-06 | adenocarcinoma |
| ILMN_2766455 | 231549 | Lrrc8d | 1.041 | 10.808 | 1.50E-06 | adenocarcinoma |
| ILMN_2698728 | 68792 | Srpx2 | 1.267 | 9.126 | 1.52E-06 | adenocarcinoma |
| ILMN_2760199 | 16612 | Klk1 | -1.501 | 13.861 | 1.58E-06 | adenocarcinoma |
| ILMN_2960982 | 18631 | Pex11a | -1.120 | 9.871 | 1.62E-06 | adenocarcinoma |
| ILMN_2543929 | 66311 | Cenpw | 1.073 | 9.162 | 1.65E-06 | adenocarcinoma |
| ILMN_2923445 | 11475 | Acta2 | -1.674 | 12.631 | 1.66E-06 | adenocarcinoma |
| ILMN_3033922 | 381629 | Atraid | -1.069 | 11.304 | 1.66E-06 | adenocarcinoma |
| ILMN_2684205 | 74645 | Fam46c | 1.093 | 8.764 | 1.71E-06 | adenocarcinoma |
| ILMN_2590884 | 18793 | Plaur | 1.306 | 8.685 | 1.73E-06 | adenocarcinoma |
| ILMN_2810882 | 19038 | Ppic | 1.087 | 11.018 | 1.74E-06 | adenocarcinoma |
| ILMN_2681241 | 11799 | Birc5 | 1.606 | 8.872 | 1.78E-06 | adenocarcinoma |
| ILMN_2628822 | 23960 | Oas1g | -1.465 | 11.363 | 1.79E-06 | adenocarcinoma |
| ILMN_2595597 | 68743 | Anln | 1.459 | 9.383 | 1.80E-06 | adenocarcinoma |
| ILMN_2792351 | 70101 | Cyp4f16 | -1.511 | 10.629 | 1.81E-06 | adenocarcinoma |
| ILMN_2511913 | 22418 | Wnt5a | 1.098 | 8.618 | 1.82E-06 | adenocarcinoma |
| ILMN_3002181 | 105675 | Ppif | -1.181 | 13.364 | 1.83E-06 | adenocarcinoma |
| ILMN_2426079 | 83924 | Gpr137b | -1.086 | 9.661 | 1.84E-06 | adenocarcinoma |
| ILMN_2987863 | 18627 | Per2 | -1.102 | 9.989 | 1.90E-06 | adenocarcinoma |
| ILMN_2679386 | 227753 | Gsn | -1.237 | 8.912 | 1.92E-06 | adenocarcinoma |
| ILMN_2655571 | 227327 | B3gnt7 | -1.593 | 10.324 | 1.95E-06 | adenocarcinoma |
| ILMN_2903511 | 50783 | Lsm4 | 1.159 | 12.713 | 1.95E-06 | adenocarcinoma |
| ILMN_2619330 | 12795 | Plk3 | -2.260 | 11.233 | 1.98E-06 | adenocarcinoma |
| ILMN_3125606 | 52668 | Ifi27 | -1.214 | 12.528 | 1.99E-06 | adenocarcinoma |
| ILMN_2515816 | 58187 | Cldn10 | -1.154 | 9.185 | 2.02E-06 | adenocarcinoma |
| ILMN_3112873 | 56338 | Txnip | -1.273 | 13.964 | 2.08E-06 | adenocarcinoma |
| ILMN_1235179 | 229593 | Golph3l | -1.025 | 9.809 | 2.13E-06 | adenocarcinoma |
| ILMN_1246816 | 69097 | Trim15 | 1.552 | 9.441 | 2.15E-06 | adenocarcinoma |
| ILMN_2740902 | 17345 | Mki67 | 1.670 | 9.069 | 2.16E-06 | adenocarcinoma |
| ILMN_2603699 | 11799 | Birc5 | 1.508 | 8.898 | 2.23E-06 | adenocarcinoma |
| ILMN_1231795 | 215951 | Lace1 | -1.161 | 9.726 | 2.23E-06 | adenocarcinoma |
| ILMN_2852624 | 66438 | Hamp2 | -1.764 | 9.922 | 2.26E-06 | adenocarcinoma |
| ILMN_1244991 | 231670 | Fbxo21 | 1.065 | 10.052 | 2.34E-06 | adenocarcinoma |
| ILMN_1243862 | 16156 | Il11 | 1.830 | 8.321 | 2.40E-06 | adenocarcinoma |
| ILMN_1256135 | NA | NA | -1.421 | 10.084 | 2.44E-06 | adenocarcinoma |
| ILMN_1250116 | NA | NA | 1.279 | 8.613 | 2.46E-06 | adenocarcinoma |
| ILMN_1233733 | 15516 | Hsp90ab1 | 1.023 | 10.637 | 2.47E-06 | adenocarcinoma |
| ILMN_2482821 | NA | NA | -1.317 | 8.534 | 2.56E-06 | adenocarcinoma |
| ILMN_2463181 | 21923 | Tnc | 1.288 | 9.822 | 2.57E-06 | adenocarcinoma |
| ILMN_1259482 | 18458 | Pabpc1 | 1.281 | 10.858 | 2.63E-06 | adenocarcinoma |
| ILMN_2959247 | 22210 | Ube2b | -1.037 | 12.692 | 2.64E-06 | adenocarcinoma |
| ILMN_1226683 | 16913 | Psmb8 | -1.381 | 11.087 | 2.81E-06 | adenocarcinoma |
| ILMN_1220340 | 268417 | Zkscan17 | 1.348 | 10.210 | 2.87E-06 | adenocarcinoma |
| ILMN_2731989 | 14042 | Ext1 | 1.176 | 10.701 | 2.96E-06 | adenocarcinoma |
| ILMN_1237832 | NA | NA | 1.089 | 8.859 | 3.00E-06 | adenocarcinoma |
| ILMN_2669416 | 56615 | Mgst1 | -1.162 | 10.274 | 3.00E-06 | adenocarcinoma |
| ILMN_2844820 | 654812 | Angptl7 | -1.340 | 9.543 | 3.05E-06 | adenocarcinoma |
| ILMN_3043469 | 19713 | Ret | 1.176 | 8.306 | 3.07E-06 | adenocarcinoma |
| ILMN_2813859 | 19660 | Rbp2 | -1.198 | 8.839 | 3.12E-06 | adenocarcinoma |
| ILMN_2774537 | 50708 | Hist1h1c | -1.030 | 13.627 | 3.31E-06 | adenocarcinoma |
| ILMN_2679704 | 232409 | Clec2e | -1.896 | 11.296 | 3.32E-06 | adenocarcinoma |
| ILMN_2879534 | 56219 | Extl1 | 1.248 | 8.236 | 3.51E-06 | adenocarcinoma |
| ILMN_2463180 | 21923 | Tnc | 1.226 | 9.553 | 3.69E-06 | adenocarcinoma |
| ILMN_1221684 | 20280 | Scp2 | 1.238 | 10.779 | 3.71E-06 | adenocarcinoma |
| ILMN_2987862 | 18627 | Per2 | -1.284 | 10.859 | 3.81E-06 | adenocarcinoma |
| ILMN_1215722 | 243983 | Zdhhc13 | -1.190 | 9.812 | 3.82E-06 | adenocarcinoma |
| ILMN_2627193 | 15478 | Hs3st3a1 | 1.090 | 8.727 | 3.85E-06 | adenocarcinoma |
| ILMN_2693895 | 11475 | Acta2 | -1.849 | 14.726 | 4.03E-06 | adenocarcinoma |
| ILMN_2732163 | 18300 | Oit1 | -1.200 | 11.883 | 4.32E-06 | adenocarcinoma |
| ILMN_2826881 | 17865 | Mybl2 | 1.111 | 8.460 | 4.36E-06 | adenocarcinoma |
| ILMN_2599782 | 16362 | Irf1 | -1.093 | 11.748 | 4.54E-06 | adenocarcinoma |
| ILMN_2754364 | 17002 | Ltf | 1.716 | 8.711 | 4.63E-06 | adenocarcinoma |
| ILMN_2635229 | 21826 | Thbs2 | 1.320 | 9.718 | 4.67E-06 | adenocarcinoma |
| ILMN_1250469 | 80288 | Bcl9l | 1.308 | 9.004 | 4.73E-06 | adenocarcinoma |
| ILMN_2838317 | 217430 | Pqlc3 | -1.139 | 10.152 | 4.91E-06 | adenocarcinoma |
| ILMN_2899041 | 17392 | Mmp3 | 1.577 | 8.591 | 4.93E-06 | adenocarcinoma |
| ILMN_2946520 | 109648 | Npy | 1.448 | 8.505 | 4.96E-06 | adenocarcinoma |
| ILMN_3163255 | 231293 | Cwh43 | 1.425 | 10.492 | 5.00E-06 | adenocarcinoma |
| ILMN_2797928 | 66175 | Mustn1 | -1.290 | 10.161 | 5.07E-06 | adenocarcinoma |
| ILMN_1250011 | 22057 | Tob1 | -1.028 | 11.202 | 5.07E-06 | adenocarcinoma |
| ILMN_2477221 | 17684 | Cited2 | -1.046 | 11.018 | 5.13E-06 | adenocarcinoma |
| ILMN_2696110 | 72948 | Tppp | -1.181 | 9.650 | 5.54E-06 | adenocarcinoma |
| ILMN_3143577 | 11783 | Apaf1 | 1.043 | 11.061 | 6.19E-06 | adenocarcinoma |
| ILMN_1226767 | 78354 | 2210407C18Rik | 1.329 | 9.118 | 6.25E-06 | adenocarcinoma |
| ILMN_2548010 | 74318 | Hopx | 1.065 | 11.055 | 6.85E-06 | adenocarcinoma |
| ILMN_1225712 | 100038734 | Gm10845 | 1.357 | 14.264 | 7.09E-06 | adenocarcinoma |
| ILMN_2733524 | 106582 | Nrm | 1.607 | 9.778 | 7.49E-06 | adenocarcinoma |
| ILMN_2756435 | 12608 | Cebpb | 1.081 | 10.715 | 7.58E-06 | adenocarcinoma |
| ILMN_2425029 | 22113 | Phlda2 | 1.497 | 8.803 | 8.36E-06 | adenocarcinoma |
| ILMN_2640239 | 217430 | Pqlc3 | -1.037 | 9.697 | 8.47E-06 | adenocarcinoma |
| ILMN_1247933 | 17110 | Lyz1 | 1.639 | 9.534 | 8.50E-06 | adenocarcinoma |
| ILMN_2466335 | 11783 | Apaf1 | 1.003 | 10.967 | 8.55E-06 | adenocarcinoma |
| ILMN_2789239 | 21814 | Tgfbr3 | -1.048 | 9.557 | 8.64E-06 | adenocarcinoma |
| ILMN_2629648 | 668661 | 2410002F23Rik | 1.278 | 10.129 | 8.81E-06 | adenocarcinoma |
| ILMN_1228535 | 18858 | Pmp22 | -1.371 | 12.197 | 8.82E-06 | adenocarcinoma |
| ILMN_3141106 | 15891 | Ibsp | 1.165 | 8.180 | 8.83E-06 | adenocarcinoma |
| ILMN_1238276 | 319191 | Hist1h2ai | 1.966 | 11.818 | 9.62E-06 | adenocarcinoma |
| ILMN_2664739 | 13522 | Adam28 | 1.019 | 8.020 | 9.69E-06 | adenocarcinoma |
| ILMN_2578147 | NA | NA | -1.008 | 9.883 | 9.80E-06 | adenocarcinoma |
| ILMN_2775157 | 11846 | Arg1 | 1.960 | 8.550 | 9.88E-06 | adenocarcinoma |
| ILMN_1241864 | 69864 | 1810065E05Rik | -1.522 | 13.086 | 1.08E-05 | adenocarcinoma |
| ILMN_1223257 | 20303 | Ccl4 | 1.052 | 8.314 | 1.08E-05 | adenocarcinoma |
| ILMN_1258961 | NA | NA | 1.351 | 10.675 | 1.15E-05 | adenocarcinoma |
| ILMN_1226839 | NA | NA | 2.016 | 13.001 | 1.18E-05 | adenocarcinoma |
| ILMN_2566477 | NA | NA | 1.045 | 9.274 | 1.20E-05 | adenocarcinoma |
| ILMN_1213609 | 56338 | Txnip | -1.103 | 15.411 | 1.25E-05 | adenocarcinoma |
| ILMN_3009225 | 11425 | Apoc4 | 2.907 | 8.922 | 1.27E-05 | adenocarcinoma |
| ILMN_2825848 | 211798 | Mfsd9 | -1.029 | 9.877 | 1.30E-05 | adenocarcinoma |
| ILMN_1236588 | 12346 | Car1 | -1.612 | 14.018 | 1.39E-05 | adenocarcinoma |
| ILMN_1222219 | 70045 | 2610528A11Rik | -1.529 | 12.549 | 1.47E-05 | adenocarcinoma |
| ILMN_2997494 | NA | NA | 1.051 | 8.906 | 1.54E-05 | adenocarcinoma |
| ILMN_1221168 | 66965 | Ctu2 | 1.053 | 10.961 | 1.62E-05 | adenocarcinoma |
| ILMN_2649478 | 216134 | Pdxk | 1.012 | 9.185 | 1.63E-05 | adenocarcinoma |
| ILMN_2666312 | 213989 | Tmem82 | -1.101 | 10.679 | 1.65E-05 | adenocarcinoma |
| ILMN_1234453 | 19652 | Rbm3 | 1.154 | 12.540 | 1.67E-05 | adenocarcinoma |
| ILMN_2836654 | NA | NA | 1.954 | 13.353 | 1.74E-05 | adenocarcinoma |
| ILMN_3131679 | 24110 | Usp18 | 2.239 | 9.794 | 1.89E-05 | adenocarcinoma |
| ILMN_1217849 | 16792 | Laptm5 | 1.312 | 11.341 | 2.04E-05 | adenocarcinoma |
| ILMN_2795040 | NA | NA | 2.059 | 11.418 | 2.06E-05 | adenocarcinoma |
| ILMN_1225182 | 14793 | Cdca3 | 1.736 | 9.660 | 2.08E-05 | adenocarcinoma |
| ILMN_1257159 | 229927 | Clca4 | 1.645 | 10.218 | 2.08E-05 | adenocarcinoma |
| ILMN_1219574 | 319173 | Hist1h2af | 2.037 | 11.121 | 2.09E-05 | adenocarcinoma |
| ILMN_1236387 | 72103 | Aplf | -1.060 | 10.174 | 2.24E-05 | adenocarcinoma |
| ILMN_2811154 | 20310 | Cxcl2 | 1.124 | 8.175 | 2.27E-05 | adenocarcinoma |
| ILMN_2775307 | 16782 | Lamc2 | 1.180 | 10.400 | 2.60E-05 | adenocarcinoma |
| ILMN_2888834 | 171504 | Apobr | 1.005 | 8.486 | 2.67E-05 | adenocarcinoma |
| ILMN_2498108 | 195018 | Zzef1 | -1.009 | 11.843 | 2.70E-05 | adenocarcinoma |
| ILMN_2629591 | 11886 | Asah1 | -1.017 | 10.125 | 2.73E-05 | adenocarcinoma |
| ILMN_2589859 | 66811 | Duoxa2 | 1.228 | 9.910 | 2.76E-05 | adenocarcinoma |
| ILMN_2648669 | 93695 | Gpnmb | 1.229 | 10.007 | 2.95E-05 | adenocarcinoma |
| ILMN_1222246 | 11520 | Plin2 | -1.028 | 9.997 | 2.96E-05 | adenocarcinoma |
| ILMN_2776265 | 19660 | Rbp2 | -1.190 | 8.728 | 3.02E-05 | adenocarcinoma |
| ILMN_2574754 | NA | NA | 1.049 | 8.992 | 3.09E-05 | adenocarcinoma |
| ILMN_1254630 | 19267 | Ptpre | 1.093 | 11.109 | 3.10E-05 | adenocarcinoma |
| ILMN_2653765 | 108907 | Nusap1 | 1.135 | 8.855 | 3.21E-05 | adenocarcinoma |
| ILMN_2761205 | 80888 | Hspb8 | 1.007 | 11.168 | 3.22E-05 | adenocarcinoma |
| ILMN_3159435 | 17318 | Mid1 | 1.040 | 8.499 | 3.43E-05 | adenocarcinoma |
| ILMN_2761645 | 59083 | Fetub | 1.099 | 8.232 | 3.49E-05 | adenocarcinoma |
| ILMN_2603647 | 93694 | Clec2d | -1.555 | 11.528 | 3.54E-05 | adenocarcinoma |
| ILMN_1244689 | 627860 | Cyp2d37-ps | -1.010 | 9.185 | 3.58E-05 | adenocarcinoma |
| ILMN_1248830 | 319170 | Hist1h2an | 1.741 | 10.180 | 3.71E-05 | adenocarcinoma |
| ILMN_2772077 | 51800 | Bok | 1.322 | 10.833 | 3.99E-05 | adenocarcinoma |
| ILMN_2977558 | 13143 | Dapk2 | -1.012 | 11.948 | 4.08E-05 | adenocarcinoma |
| ILMN_1218380 | 319176 | Hist2h2ac | 1.400 | 13.753 | 4.36E-05 | adenocarcinoma |
| ILMN_2730329 | 319168 | Hist1h2ah | 1.963 | 11.199 | 4.37E-05 | adenocarcinoma |
| ILMN_2658461 | 66175 | Mustn1 | -1.072 | 10.355 | 4.53E-05 | adenocarcinoma |
| ILMN_2434853 | NA | NA | -1.017 | 10.573 | 4.55E-05 | adenocarcinoma |
| ILMN_2745212 | NA | NA | 1.301 | 13.853 | 4.62E-05 | adenocarcinoma |
| ILMN_2718401 | 78558 | Htra3 | -1.035 | 9.373 | 4.84E-05 | adenocarcinoma |
| ILMN_2835423 | 11537 | Cfd | -4.198 | 11.495 | 5.17E-05 | adenocarcinoma |
| ILMN_2662926 | 13653 | Egr1 | 1.698 | 10.359 | 5.38E-05 | adenocarcinoma |
| ILMN_2593736 | 20731 | Spink4 | 1.026 | 14.049 | 5.40E-05 | adenocarcinoma |
| ILMN_2878060 | 11749 | Anxa6 | 1.267 | 9.773 | 5.66E-05 | adenocarcinoma |
| ILMN_2749669 | 217166 | Nr1d1 | -1.304 | 9.078 | 5.69E-05 | adenocarcinoma |
| ILMN_2740852 | 14062 | F2r | 1.068 | 11.875 | 5.93E-05 | adenocarcinoma |
| ILMN_1246108 | 319168 | Hist1h2ah | 1.816 | 10.874 | 5.98E-05 | adenocarcinoma |
| ILMN_2759484 | 12266 | C3 | -1.592 | 11.663 | 6.09E-05 | adenocarcinoma |
| ILMN_2847144 | 319169 | Hist1h2ak | 1.952 | 11.170 | 6.12E-05 | adenocarcinoma |
| ILMN_2753714 | 331063 | Gsdmc2 | -1.342 | 12.342 | 6.18E-05 | adenocarcinoma |
| ILMN_2544003 | NA | NA | 1.034 | 9.307 | 6.38E-05 | adenocarcinoma |
| ILMN_1253808 | 23960 | Oas1g | -1.046 | 11.821 | 6.48E-05 | adenocarcinoma |
| ILMN_2629191 | 70574 | Cpm | -1.032 | 11.894 | 7.16E-05 | adenocarcinoma |
| ILMN_2655929 | 67971 | Tppp3 | -1.326 | 9.615 | 7.30E-05 | adenocarcinoma |
| ILMN_1250138 | 17829 | Muc1 | -1.024 | 9.568 | 7.59E-05 | adenocarcinoma |
| ILMN_1255287 | NA | NA | 3.037 | 9.927 | 8.55E-05 | adenocarcinoma |
| ILMN_2433990 | 24110 | Usp18 | 1.674 | 9.271 | 8.61E-05 | adenocarcinoma |
| ILMN_1229091 | 217166 | Nr1d1 | -1.488 | 9.998 | 9.39E-05 | adenocarcinoma |
| ILMN_1216368 | 108105 | B3gnt5 | -1.059 | 11.647 | 1.00E-04 | adenocarcinoma |
| ILMN_1216092 | 77397 | 9530003J23Rik | 1.107 | 8.111 | 1.04E-04 | adenocarcinoma |
| ILMN_2676052 | 21685 | Tef | -1.220 | 9.947 | 1.05E-04 | adenocarcinoma |
| ILMN_2706819 | 68939 | Rasl11b | 1.181 | 9.948 | 1.18E-04 | adenocarcinoma |
| ILMN_2544056 | 100503605 | Hbb-bs | 1.156 | 9.217 | 1.25E-04 | adenocarcinoma |
| ILMN_1236718 | NA | NA | 1.894 | 13.152 | 1.29E-04 | adenocarcinoma |
| ILMN_1244316 | NA | NA | 1.766 | 13.425 | 1.34E-04 | adenocarcinoma |
| ILMN_2428301 | 12747 | Clk1 | -1.075 | 11.563 | 1.38E-04 | adenocarcinoma |
| ILMN_3139875 | 26897 | Acot1 | 1.323 | 10.264 | 1.49E-04 | adenocarcinoma |
| ILMN_1231445 | 21743 | Inmt | -2.366 | 11.337 | 1.50E-04 | adenocarcinoma |
| ILMN_1241437 | 14859 | Gsta3 | 1.438 | 10.337 | 1.68E-04 | adenocarcinoma |
| ILMN_2723108 | 21807 | Tsc22d1 | -1.186 | 11.488 | 1.89E-04 | adenocarcinoma |
| ILMN_2769884 | 16000 | Igf1 | 1.003 | 8.747 | 1.95E-04 | adenocarcinoma |
| ILMN_2887421 | 18489 | Reg3b | 1.707 | 8.416 | 1.97E-04 | adenocarcinoma |
| ILMN_2612895 | 12263 | C2 | -1.114 | 10.274 | 2.23E-04 | adenocarcinoma |
| ILMN_1216452 | NA | NA | 1.709 | 11.648 | 2.44E-04 | adenocarcinoma |
| ILMN_2705860 | 100503895 | Tpsab1 | 1.699 | 8.491 | 2.45E-04 | adenocarcinoma |
| ILMN_2606295 | 14528 | Gch1 | 1.013 | 11.027 | 2.54E-04 | adenocarcinoma |
| ILMN_1223244 | NA | NA | 1.778 | 12.237 | 2.69E-04 | adenocarcinoma |
| ILMN_2722616 | 16664 | Krt14 | 1.572 | 10.009 | 2.78E-04 | adenocarcinoma |
| ILMN_2772274 | 20384 | Srsf5 | -1.073 | 12.198 | 2.90E-04 | adenocarcinoma |
| ILMN_2981801 | NA | NA | 1.318 | 9.307 | 2.91E-04 | adenocarcinoma |
| ILMN_1259344 | 22264 | Prap1 | 1.084 | 8.230 | 2.91E-04 | adenocarcinoma |
| ILMN_3054914 | 24110 | Usp18 | 1.230 | 8.736 | 3.17E-04 | adenocarcinoma |
| ILMN_1225061 | NA | NA | -2.128 | 8.804 | 3.67E-04 | adenocarcinoma |
| ILMN_2450155 | NA | NA | -1.515 | 10.284 | 3.69E-04 | adenocarcinoma |
| ILMN_2793062 | 68939 | Rasl11b | 1.155 | 10.174 | 4.34E-04 | adenocarcinoma |
| ILMN_1251725 | 68468 | Ly6g6c | -1.157 | 10.507 | 4.54E-04 | adenocarcinoma |
| ILMN_1235361 | 75697 | C2cd4b | 1.537 | 9.311 | 4.63E-04 | adenocarcinoma |
| ILMN_2803249 | 21743 | Inmt | -1.883 | 10.046 | 4.65E-04 | adenocarcinoma |
| ILMN_2548974 | NA | NA | 1.054 | 9.796 | 5.46E-04 | adenocarcinoma |
| ILMN_2545963 | NA | NA | 1.569 | 11.546 | 5.49E-04 | adenocarcinoma |
| ILMN_3160292 | 432720 | Akr1c19 | -1.097 | 12.054 | 5.70E-04 | adenocarcinoma |
| ILMN_3115255 | 331063 | Gsdmc2 | -1.065 | 11.713 | 6.22E-04 | adenocarcinoma |
| ILMN_1212702 | NA | NA | 1.725 | 13.043 | 6.73E-04 | adenocarcinoma |
| ILMN_2762944 | 76933 | Ifi27l2a | -1.209 | 9.830 | 6.87E-04 | adenocarcinoma |
| ILMN_2702687 | 15430 | Hoxd10 | -1.279 | 9.743 | 7.15E-04 | adenocarcinoma |
| ILMN_1229203 | NA | NA | 1.544 | 12.237 | 8.16E-04 | adenocarcinoma |
| ILMN_2630047 | 12350 | Car3 | -2.368 | 9.580 | 1.27E-03 | adenocarcinoma |
| ILMN_1252121 | 66660 | Sltm | 2.431 | 8.815 | 1.32E-03 | adenocarcinoma |
| ILMN_1239117 | NA | NA | 1.310 | 10.490 | 1.55E-03 | adenocarcinoma |
| ILMN_2738082 | 11450 | Adipoq | -2.156 | 9.665 | 1.65E-03 | adenocarcinoma |
| ILMN_2621328 | 75646 | Rai14 | 1.280 | 7.320 | 2.02E-03 | adenocarcinoma |
| ILMN_2499056 | NA | NA | -1.224 | 9.060 | 2.06E-03 | adenocarcinoma |
| ILMN_1229263 | NA | NA | -1.260 | 8.989 | 2.28E-03 | adenocarcinoma |
| ILMN_2730208 | NA | NA | -2.752 | 9.374 | 3.29E-03 | adenocarcinoma |
| ILMN_1246353 | NA | NA | -1.049 | 8.854 | 3.81E-03 | adenocarcinoma |
| ILMN_1256817 | 20568 | Slpi | 1.303 | 11.497 | 4.14E-03 | adenocarcinoma |
| ILMN_2944824 | 15439 | Hp | 1.734 | 9.051 | 4.82E-03 | adenocarcinoma |
| ILMN_1216042 | 11816 | Apoe | 1.120 | 10.484 | 5.01E-03 | adenocarcinoma |
| ILMN_2467429 | NA | NA | -1.219 | 8.999 | 6.44E-03 | adenocarcinoma |
| ILMN_1212878 | 70956 | Tex19.2 | 1.556 | 8.147 | 7.55E-03 | adenocarcinoma |
| ILMN_1251419 | NA | NA | 1.489 | 8.313 | 2.57E-02 | adenocarcinoma |
| ILMN_2687014 | 13106 | Cyp2e1 | -1.619 | 9.831 | 3.57E-02 | adenocarcinoma |
| ILMN_2599794 | 11812 | Apoc1 | -1.193 | 8.928 | 4.35E-02 | adenocarcinoma |
| ILMN_1247199 | 26914 | H2afy | -2.607 | 8.712 | 3.08E-15 | AOM |
| ILMN_1248727 | NA | NA | 2.580 | 9.673 | 1.05E-14 | AOM |
| ILMN_2737090 | 66184 | Rps4y2 | -1.516 | 8.432 | 1.55E-09 | AOM |
| ILMN_1242842 | 26914 | H2afy | -1.287 | 10.028 | 1.98E-08 | AOM |
| ILMN_1236079 | 229599 | Gm129 | -1.146 | 8.215 | 1.75E-07 | AOM |
| ILMN_2875737 | 380997 | Cyp2d12 | -1.518 | 10.822 | 1.75E-07 | AOM |
| ILMN_2999670 | 225742 | St8sia5 | -2.299 | 9.065 | 1.75E-07 | AOM |
| ILMN_2485639 | 21990 | Tph1 | 1.118 | 8.877 | 2.82E-07 | AOM |
| ILMN_1212989 | 26914 | H2afy | -1.414 | 11.165 | 6.73E-07 | AOM |
| ILMN_2455390 | 207952 | Klhl25 | -1.058 | 8.242 | 6.88E-07 | AOM |
| ILMN_3102736 | 229599 | Gm129 | -1.573 | 8.757 | 1.21E-06 | AOM |
| ILMN_2679377 | 12653 | Chgb | 1.565 | 10.462 | 2.95E-06 | AOM |
| ILMN_2596522 | 17748 | Mt1 | -1.596 | 14.813 | 3.45E-06 | AOM |
| ILMN_3029489 | 229599 | Gm129 | -1.646 | 8.929 | 7.08E-06 | AOM |
| ILMN_1256795 | 67204 | Eif2s2 | -1.191 | 11.797 | 7.47E-06 | AOM |
| ILMN_1215552 | 108017 | Fxyd4 | -1.921 | 9.428 | 7.47E-06 | AOM |
| ILMN_2670751 | 108017 | Fxyd4 | -2.225 | 10.232 | 1.04E-05 | AOM |
| ILMN_2648669 | 93695 | Gpnmb | 1.814 | 10.007 | 1.26E-05 | AOM |
| ILMN_1240264 | 53376 | Usp2 | -1.139 | 9.192 | 6.67E-05 | AOM |
| ILMN_2482821 | NA | NA | -1.440 | 8.534 | 7.90E-05 | AOM |
| ILMN_2928489 | 17829 | Muc1 | -1.363 | 9.125 | 8.70E-05 | AOM |
| ILMN_1231229 | 17829 | Muc1 | -1.101 | 9.080 | 8.93E-05 | AOM |
| ILMN_1250138 | 17829 | Muc1 | -1.399 | 9.568 | 1.31E-04 | AOM |
| ILMN_2991019 | 393082 | Mettl7a2 | -1.831 | 9.626 | 1.93E-04 | AOM |
| ILMN_2616226 | 13170 | Dbp | -2.562 | 11.046 | 5.15E-04 | AOM |
| ILMN_1251713 | 76459 | Car12 | 1.277 | 10.376 | 7.15E-04 | AOM |
| ILMN_2960325 | 13034 | Ctse | -1.151 | 11.539 | 8.49E-04 | AOM |
| ILMN_1228582 | 192113 | Atp12a | -1.664 | 12.634 | 8.85E-04 | AOM |
| ILMN_2687507 | 13105 | Cyp2d9 | -1.199 | 11.045 | 1.29E-03 | AOM |
| ILMN_2646640 | 67080 | 1700019D03Rik | -1.004 | 11.376 | 1.40E-03 | AOM |
| ILMN_1257444 | 56857 | Slc37a2 | -1.213 | 10.937 | 2.07E-03 | AOM |
| ILMN_2904435 | 67133 | Gp2 | -1.265 | 10.004 | 3.27E-03 | AOM |
| ILMN_2826869 | 20208 | Saa1 | -1.221 | 11.156 | 3.60E-03 | AOM |
| ILMN_2725781 | 27409 | Abcg5 | -1.170 | 11.491 | 4.01E-03 | AOM |
| ILMN_1229263 | NA | NA | -1.675 | 8.989 | 5.43E-03 | AOM |
| ILMN_2654952 | 208715 | Hmgcs1 | -1.034 | 14.246 | 6.52E-03 | AOM |
| ILMN_3136561 | 20692 | Sparc | -1.013 | 10.578 | 7.42E-03 | AOM |
| ILMN_2467151 | NA | NA | -1.393 | 9.140 | 9.55E-03 | AOM |
| ILMN_1236588 | 12346 | Car1 | -1.215 | 14.018 | 1.05E-02 | AOM |
| ILMN_2467429 | NA | NA | -1.617 | 8.999 | 1.55E-02 | AOM |
| ILMN_2499056 | NA | NA | -1.393 | 9.060 | 1.60E-02 | AOM |
| ILMN_2621328 | 75646 | Rai14 | 1.366 | 7.320 | 2.45E-02 | AOM |
| ILMN_1225061 | NA | NA | -1.843 | 8.804 | 2.81E-02 | AOM |
| ILMN_1216322 | 15360 | Hmgcs2 | 1.495 | 12.650 | 3.87E-02 | AOM |
| ILMN_1247199 | 26914 | H2afy | -2.570 | 8.712 | 3.93E-15 | DSS |
| ILMN_1248727 | NA | NA | 2.691 | 9.673 | 3.93E-15 | DSS |
| ILMN_2737090 | 66184 | Rps4y2 | -1.442 | 8.432 | 4.32E-09 | DSS |
| ILMN_1242842 | 26914 | H2afy | -1.383 | 10.028 | 4.32E-09 | DSS |
| ILMN_2485639 | 21990 | Tph1 | 1.243 | 8.877 | 5.26E-08 | DSS |
| ILMN_1212989 | 26914 | H2afy | -1.617 | 11.165 | 6.77E-08 | DSS |
| ILMN_1236079 | 229599 | Gm129 | -1.197 | 8.215 | 6.77E-08 | DSS |
| ILMN_3102736 | 229599 | Gm129 | -1.760 | 8.757 | 1.84E-07 | DSS |
| ILMN_3029489 | 229599 | Gm129 | -1.867 | 8.929 | 1.18E-06 | DSS |
| ILMN_2679377 | 12653 | Chgb | 1.596 | 10.462 | 2.62E-06 | DSS |
| ILMN_2616226 | 13170 | Dbp | -3.683 | 11.046 | 4.09E-06 | DSS |
| ILMN_2999670 | 225742 | St8sia5 | -1.649 | 9.065 | 4.46E-05 | DSS |
| ILMN_1240264 | 53376 | Usp2 | -1.166 | 9.192 | 6.03E-05 | DSS |
| ILMN_2836137 | 242705 | E2f2 | 1.040 | 9.045 | 6.36E-05 | DSS |
| ILMN_2875737 | 380997 | Cyp2d12 | -1.043 | 10.822 | 6.48E-05 | DSS |
| ILMN_2595732 | 18030 | Nfil3 | 1.288 | 9.416 | 8.39E-05 | DSS |
| ILMN_2482821 | NA | NA | -1.407 | 8.534 | 1.32E-04 | DSS |
| ILMN_2991019 | 393082 | Mettl7a2 | -1.644 | 9.626 | 9.21E-04 | DSS |
| ILMN_2960325 | 13034 | Ctse | -1.079 | 11.539 | 1.77E-03 | DSS |
| ILMN_1215552 | 108017 | Fxyd4 | -1.267 | 9.428 | 1.88E-03 | DSS |
| ILMN_1251713 | 76459 | Car12 | 1.165 | 10.376 | 1.94E-03 | DSS |
| ILMN_1250138 | 17829 | Muc1 | -1.104 | 9.568 | 1.94E-03 | DSS |
| ILMN_2676052 | 21685 | Tef | -1.338 | 9.947 | 2.17E-03 | DSS |
| ILMN_2928489 | 17829 | Muc1 | -1.009 | 9.125 | 2.81E-03 | DSS |
| ILMN_2670751 | 108017 | Fxyd4 | -1.424 | 10.232 | 2.94E-03 | DSS |
| ILMN_3136561 | 20692 | Sparc | -1.074 | 10.578 | 5.73E-03 | DSS |
| ILMN_1242170 | 11551 | Adra2a | 1.230 | 10.687 | 7.68E-03 | DSS |
| ILMN_1257444 | 56857 | Slc37a2 | -1.037 | 10.937 | 8.74E-03 | DSS |
| ILMN_2589859 | 66811 | Duoxa2 | 1.004 | 9.910 | 1.01E-02 | DSS |
| ILMN_1236588 | 12346 | Car1 | -1.115 | 14.018 | 2.45E-02 | DSS |
| ILMN_1229091 | 217166 | Nr1d1 | -1.202 | 9.998 | 2.45E-02 | DSS |
| ILMN_2621328 | 75646 | Rai14 | 1.395 | 7.320 | 2.57E-02 | DSS |
| ILMN_1252121 | 66660 | Sltm | 2.365 | 8.815 | 3.75E-02 | DSS |
| ILMN_2625893 | 104158 | Ces1d | 1.127 | 11.210 | 3.84E-02 | DSS |
| ILMN_1228582 | 192113 | Atp12a | -1.046 | 12.634 | 3.84E-02 | DSS |
| ILMN_1229263 | NA | NA | -1.249 | 8.989 | 4.76E-02 | DSS |

The fourth column shows the Log2FC of adenocarcinoma, AOM-treated mucosa and DSS-treated mucosa, respectively, vs normal mucosa. The significance level is indicated by a corrected p-value.
